# Metagenomic Analysis of Plant Virus Occurrence in Common Bean (*Phaseolus vulgaris*) in Central Kenya

**DOI:** 10.3389/fmicb.2018.02939

**Published:** 2018-12-07

**Authors:** J. Musembi Mutuku, Francis O. Wamonje, Gerardine Mukeshimana, Joyce Njuguna, Mark Wamalwa, Seung-Kook Choi, Trisna Tungadi, Appolinaire Djikeng, Krys Kelly, Jean-Baka Domelevo Entfellner, Sita R. Ghimire, Hodeba D. Mignouna, John P. Carr, Jagger J. W. Harvey

**Affiliations:** ^1^Biosciences Eastern and Central Africa, International Livestock Research Institute, Nairobi, Kenya; ^2^Department of Plant Sciences, University of Cambridge, Cambridge, United Kingdom; ^3^Biotechnology Department, Kenyatta University, Nairobi, Kenya; ^4^Department of Vegetable Research, National Institute of Horticultural and Herbal Science, Rural Development Agency, Wanju County, South Korea

**Keywords:** viral metagenomics, plant-virus interactions, insect vectors, vertical transmission, *endornavirus*, *cucumovirus*, *potyvirus*, maize lethal necrosis

## Abstract

Two closely related potyviruses, bean common mosaic virus (BCMV) and bean common mosaic necrosis virus (BCMNV), are regarded as major constraints on production of common bean (*Phaseolus vulgaris* L.) in Eastern and Central Africa, where this crop provides a high proportion of dietary protein as well as other nutritional, agronomic, and economic benefits. Previous studies using antibody-based assays and indicator plants indicated that BCMV and BCMNV are both prevalent in bean fields in the region but these approaches cannot distinguish between these potyviruses or detect other viruses that may threaten the crop. In this study, we utilized next generation shotgun sequencing for a metagenomic examination of viruses present in bean plants growing at two locations in Kenya: the University of Nairobi Research Farm in Nairobi's Kabete district and at sites in Kirinyaga County. RNA was extracted from leaves of bean plants exhibiting apparent viral symptoms and sequenced on the Illumina MiSeq platform. We detected BCMNV, cucumber mosaic virus (CMV), and *Phaseolus vulgaris* alphaendornaviruses 1 and 2 (PvEV1 and 2), with CMV present in the Kirinyaga samples. The CMV strain detected in this study was most closely related to Asian strains, which suggests that it may be a recent introduction to the region. Surprisingly, and in contrast to previous surveys, BCMV was not detected in plants at either location. Some plants were infected with PvEV1 and 2. The detection of PvEV1 and 2 suggests these seed transmitted viruses may be more prevalent in Eastern African bean germplasm than previously thought.

## Introduction

In Eastern and Central Africa, common bean (*Phaseolus vulgaris* L.) is a vital crop that naturally enriches the soil with nitrogen, providing natural fertilizer for other important crops such as maize and cassava (Allen, 1995; Broughton et al., 2003; Mucheru-Muna et al., 2010). Common bean is also an important part of the regional diet because it is rich in protein and micronutrients and its storability makes it an important element of food security (CIAT, 2009). In Rwanda, for example, average daily common bean consumption is 200 g per capita per day, which provides up to 60% of dietary protein intake (Jansa et al., 2011). Trading of common bean provides important economic benefits both to farmers and national economies in the region (CIAT, 2009). Indeed, common bean contributes around US$ 400 million annually to the national economy of Kenya, which is the seventh highest producer after Brazil, India, China, Myanmar, Mexico, and the USA (Kenya Ministry of Agriculture, 2015).

Pests and diseases, and viruses in particular, are major constraints on bean production in East and Central Africa (Morales, 2006; Worrall et al., 2015; Mwaipopo et al., 2017). Among the most important viral pathogens limiting common bean production are two closely related potyviruses, bean common mosaic virus (BCMV), and bean common mosaic necrosis virus (BCMNV) (Morales, 2006; Worrall et al., 2015; Mwaipopo et al., 2017). In a study carried out two decades ago, both potyviruses were found to be prevalent in seven districts within Uganda (Sengooba et al., 1997). In a more recent study, they were detected in western Kenyan counties immediately adjacent to the Ugandan border and Lake Victoria (Mangeni et al., 2014). These studies, however, were dependent upon immunological methods to identify BCMV and BCMNV based on their coat protein (CP) antigenic properties and the use of differential indicator hosts. These methods do not allow detailed analysis of BCMV or BCMNV RNA sequence variation or the presence of novel or recombinant strains, which may potentially include resistance-breaking strains (Larsen et al., 2005; Feng et al., 2014). Additionally, these approaches cannot detect other viruses present. For example, the cucumovirus, cucumber mosaic virus is (like BCMV and BCMNV) vectored non-persistently by aphids (horizontal transmission) and transmitted vertically through seed (Morales, 2006), and can cause serious disease epidemics in common bean (Gildow et al., 2008; Thompson et al., 2015). Perhaps more importantly, such focused methods will not reveal the presence of unsuspected or novel viruses infecting the crop or the presence of co-infecting viruses in the same plant (Syller, 2012).

Metagenomics is not limited by such factors and has dramatically extended our knowledge of plant virus biodiversity, (Roossinck et al., 2015). Metagenomics is the analysis of microbial and viral populations in environmental samples through nucleic acid sequencing, in particular, by using “next-generation” sequencing methods (reviewed by Roossinck et al., 2015). Motivations for performing plant virus metagenomics include: identifying causes of viral diseases in crops; screening for specific viruses when their presence is suspected; detection of asymptomatic or cryptic viruses, and the discovery of novel viruses and other microorganisms (MacDiarmid et al., 2013; Roossinck et al., 2015).

In this study, we used metagenomics to investigate viruses in samples of common bean leaves obtained from farmers' fields in two regions of Kenya to identify the currently occurring bean-infecting viruses. In contrast to previous surveys, we found that BCMV may be less common, with BCMNV being the only bean-infecting potyvirus detected. Additionally, we detected CMV, which has not been considered to be a particularly common virus of bean in Eastern Africa. We also detected the *Phaseolus vulgaris* alphaendornaviruses (PvEV) 1 and 2. These viruses have nicked dsRNA genomes and infect plants, fungi and oomycetes, and are transmitted vertically but not horizontally (Okada et al., 2011). We believe this to be the first application of viral metagenomics to assess virus occurrence in this important crop in East Africa (Mwaipopo et al., 2017).

## Methods

### Sample Collection, Nucleic Acid Extraction, and Next-Generation Sequencing

**Figure 1:**
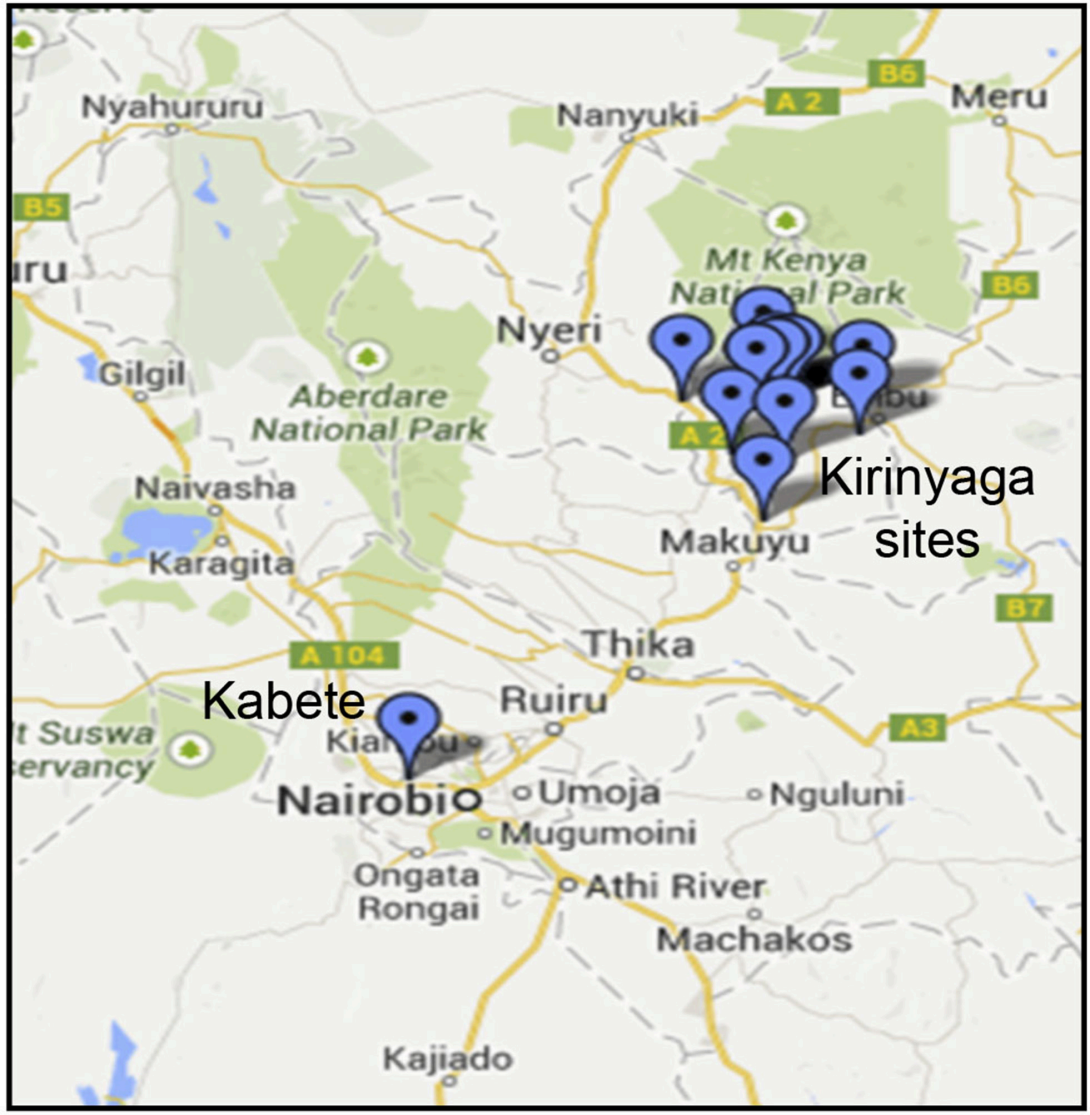
Sampling sites in Kenya showing locations in Kirinyaga County and the location of the University of Nairobi Research Farm in Kabete.

Common bean (*Phaseolus vulgaris* L.) leaf samples showing virus-like disease symptoms (Supplemental Figure S1) were collected from two sites: the University of Nairobi Research Farm in Nairobi's Kabete district, and from fields on a total of six farms in Kirinyaga County in Central Kenya (Figure 1; Table 1). Sampled leaf material (the youngest trifoliate leaves) sampled from 21 plants at each location was flash frozen in liquid N_2_ and transported to the laboratory at the BecA-ILRI Hub, Nairobi on dry ice for processing. Total RNA was extracted using TriZol reagent (Thermo Fisher, Waltham MA). RNA from three or four plants was pooled to produce each RNA sample used for library preparation. Libraries were prepared using TruSeq RNA Sample Preparation kit (Illumina, San Diego CA, USA) and sequenced using the Illumina MiSeq system following the manufacturer's instructions. RNA extracted from composite plant samples (*n* = 12) was used for construction of sequencing libraries using the Illumina TruSeq RNA Library Prep kit. Briefly, RNA samples (0.5 μg) were fragmented using the Illumina “Elute, Prime, Fragment High Mix” followed by first strand and second strand cDNA synthesis. The double stranded cDNA was purified using Agencourt AMPure XP magnetic beads (Beckman Coulter, Inc. Indianapolis, IN) followed by end-repair and adapter ligation. Ligated ds cDNA molecules were amplified by PCR using universal adapters AGAT CGG AAG AGC ACA CGT CTG AAC TCC AGT CA and AGA TCG GAA GAG CGT CGT GTA GGG AAA GAG TGT. The index primers were as shown in Supplementary Table S1. The resulting 12 libraries were purified using Agencourt AMPure XP magnetic beads. Library quality and quantity were assessed with the Agilent Tape Station 2200 system (Agilent Technologies, Santa Clara, CA) and Qubit™ fluorometer (Thermo Fisher Scientific Inc., Waltham, MA) respectively. The 12 libraries were normalized, pooled and diluted to a final concentration of 6.5 pM. Pooled libraries were run together on the Illumina MiSeq System using 12 pM of 1% PhiX as control. Paired-end sequencing was performed (2 × 300 bp). Sequencing was conducted at the BecA-ILRI Hub Nairobi Kenya.

**Table 1:** Sample information.

**Sample**	**Location**
S1	^*^Nairobi
S2	Nairobi
S3	Nairobi
S4	Nairobi
S5	Nairobi
S6	Nairobi
S7	^**^Kirinyaga
S8	Kirinyaga
S9	Kirinyaga
S10	Kirinyaga
S11	Kirinyaga
S12	Kirinyaga

* Nairobi: University of Nairobi research farm.

** *Kirinyaga: farmers' fields in the Kirinyaga district of Kenya*.

### Sequence Analysis

Sequences obtained from the 12 samples were trimmed using Trimmomatic V 0.33 (Bolger et al., 2014) to remove low quality bases and adapter sequences. Reads were mapped to the *Phaseolus vulgaris* genome to remove the host sequences using Bowtie2 V 2.2.8 (Langmead and Steven, 2012). The remaining reads of each sample were assembled *de novo* using metaSPAdes V 3.10.1 (Nurk et al., 2017) with default settings. The resulting contigs were submitted to BLAST for comparison against a local download of NCBI GenBank nucleotide database of plant viruses using BLAST+ (Camacho et al., 2009). BLAST results were visualized in Krona (Ondov et al., 2011).

Reference assemblies to construct partial viral genomes from the new sequences were carried out using CLC Genomics Workbench (https://www.qiagenbioinformatics.com/) and the reads were mapped against the most similar viral genomes. The resulting viral genomes were compared to the *de novo* assembled contigs to ensure consistency in the two approaches. The *CP* gene alignments and phylogenetic trees were constructed using MEGA v6 by with the maximum likelihood model at 1,000 bootstrap replicates (Tamura et al., 2013).

### RT-PCR and Automated Sanger Sequencing

The cDNA obtained from previous steps was subjected to PCR using virus-specific primers. For CMV the following *CP*-specific primers were used. The forward primer was 5′-ATGGACAAATCTGAATCAACCAGTGCT-3′ and the reverse primer was 5′-TCAGACTGGGAGCACTCCAGATGTGGG-3′ (Kwon et al., 2016). Primers to confirm the presence of BCMNV were designed as follows. Three full-length sequences were sourced from the NCBI nucleotide database and manually aligned in a text editor. The primer design software PriFi (Fredslund et al., 2005) was used to select primers. The primers were designed using an alignment of the following sequences from GenBank; NL5-HQ229993, NL8-HQ229994, TN1-HQ229995. Primers were designed from regions ~850 nucleotides apart. The following parameters were considered when designing the primers: amplicon length of 900–1,000 bases, amplicons to overlap by 50–100 base pairs, primer length 16–20 bases and near similar melting points for the primer pairs. The sequences for the primers used to detect BCMNV coat protein were 5′-AGA GAA TAT TCA TAC CCGC-3′ as BCMNV reverse primer and 5′-ACACAAGAGCTACCAAG-3′ as BCMNV forward primer. The presence of PvEV1 and PvEV2 were confirmed by RT-PCR using the following primers sets, PvEV1 reverse primer 5′-GATTGATTGGGCTGTATAGTG-3′ and PvEV1 forward primer 5′- GTA AACCAGGGAATTGGTGG-3′, and PvEV2 reverse primer 5′-GTTGCTGTATTGCTCGTGTC−3′ and PvEV2 forward primer 5′-TGTTAGGCGTGTGTCCCCA−3′. The PvEV1 and PvEV2 primer sequences were as previously reported (Okada et al., 2013).

Amplified DNA bands were excised using a razor blade and purified from agarose using QIAquick PCR Purification Kit (Qiagen GmbH, Mainz, Germany) following the manufacturer's instructions with slight modifications. Briefly, the samples were applied to the QIAquick column and centrifuged for 60 s at 12,000 g to bind DNA. To wash, 750 μl of buffer PE was applied to the column and centrifuged at 12,000 g for 60 s. DNA was eluted in a two-step process that involved twice adding 30 μl of water and centrifuging the column for 1 min to obtain a total volume of 60 μl. To concentrate the eluate, we evaporated the water to about 30 μl using a SpeedVac (Eppendorf, Hamburg, Germany). These concentrated samples were quantified and then diluted using water to between 50 and 70 ng.μl^−1^ before submitting for automated Sanger sequencing (Sanger et al., 1977; Smith et al., 1986) (Bioneer Corporation, Daejeon, Republic of Korea).

## Results

### Virus Diversity in Symptomatic Common Bean Leaf Samples

We obtained leaf samples from common bean plants that showed virus disease-like symptoms, including mosaic patterns (Supplemental Figure S1) from two areas in Kenya (Figure 1; Table 1). The samples were handled as described in the Methods section in preparation for Illumina sequencing. After sequencing, the data were trimmed to remove low quality reads and the resulting lengths after quality control were between 60 and 279 base pairs (Table 2). Individual sequences were assembled *de novo* and the resulting contigs were submitted to BLAST analysis.

**Table 2:** NGS statistics for each sample before and after quality control, showing the number of reads, and the range of read lengths in base pairs (bp).

**Sample**	**Reads before QC**	**Length (bp) before QC**	**Number of reads after QC**	**Length (bp) after QC**
S1	4549244	35–301	3816266	60–279
S2	8199970	35–301	6855674	60–279
S3	3886246	35–301	3257070	60–279
S4	5805282	35–301	4911542	60–279
S5	4179420	35–301	3513096	60–279
S6	4304686	35–301	3233698	60–279
S7	4078864	35–301	3355110	60–279
S8	4864772	35–301	3726700	60–279
S9	5761348	35–301	4783452	60–279
S10	3315664	35–301	2751358	60–279
S11	4241896	35–301	3470878	60–278
S12	3965366	35–301	3162536	60–279

Our results show that BCMNV, PvEV1 and 2, and CMV sequences were present (Table 3). Plant-infecting endornaviruses such as PvEV1 and 2 are nicked, double-stranded RNA viruses that are vertically transmitted via seed but in general cause no apparent disease (Roossinck et al., 2011; Okada et al., 2013). The presence of CMV was also surprising since, although it has been detected previously in eastern Africa, it is not frequently found in common bean (see Mwaipopo et al., 2017). Surprisingly, given the findings of previous studies (Sengooba et al., 1997; Mangeni et al., 2014; Worrall et al., 2015; Mwaipopo et al., 2017), no hits corresponding to BCMV sequences were generated by BLASTn searches with an e-value threshold of 1e-20 in these samples. Sequence signatures of other viruses including *Grapevine leafroll-associated virus 1, Citrus exocortis viroid, Hardenbergia mosaic virus*, and *Rice ragged stunt virus*, were detected when the sequences were applied to a general BLAST database but not followed up in detail because these signatures disappeared when an e-value threshold of 1e-20 was applied to the virus database. Subsequent analysis focused on BCMNV, CMV, and PvEV1 and 2.

**Table 3:** Virus sequences identified in each sample and identity with database sequences.

**Sample**	**Virus**	**Accession**	**Strain**	**%Identity**	**Identities**	**Length**	**Coverage**
S1	PvEV 1	AB719397.1	–	99	1062/1068	1068	2.8
	PvEV 2	AB719398.1	–	97	1560/1609	1609	3.8
S2	BCMNV	KY659305.1	1755b	99	832/842	842	4.8
S3	BCMNV	KY659305.1	1755b	99	9509/9608	9619	466.2
	PvEV 1	AB719397.1	–	99	786/790	790	2.3
	PvEV 2	AB719398.1	–	97	2292/2355	2355	3.8
S4	BCMNV	KY659304.1	NL8-CA	99	7775/7831	7846	988.8
S5	BCMNV	KY659305.1	1755b	99	9494/9609	9617	381.6
S6	BCMNV	AY282577.1	NL3	97	8962/9243	9259	1714.1
S7	BCMNV	KY659305.1	1755b	99	9517/9624	9670	737.2
S8	BCMNV	KY659304.1	NL8-CA	99	9483/9609	9629	863.6
S9	BCMNV	KX302007.1	TM70	99	9560/9622	9640	961.7
S10	BCMNV	KY659305.1	1755b	99	1044/1059	1059	2.1
	PvEV1	KT456287.1	PvEV-1	99	842/849	849	3.1
	CMV_RNA1	KJ400002.1	209	94	3004/3193	3193	28.2
	CMV_RNA2	KJ400003.1	209	92	2748/2995	2991	19.8
	CMV_RNA3	AY429437.1	–	97	2124/2199	3324	75.2
S11	BCMNV	KY659305.1	1755b	97	446/459	459	1.3
	PvEV	KT456287.1	PvEV-1	98	2008/2055	2055	5.4
	CMV_RNA1	KJ400002.1	209	94	2995/3188	3187	67.04
	CMV_RNA2	KJ400003.1	209	92	2750/2997	2992	28.03
	CMV_RNA3	AY429437.1	–	97	2134/2193	2191	124.73
S12	BCMNV	KY659304.1	NL8-CA	99	996/1004	1004	5.2
	PvEV	KT456287.1	PvEV-1	97	825/847	847	3.5
	CMV_RNA1	KJ400002.1	209	94	2990/3183	3182	28.5
	CMV_RNA2	KJ400003.1	209	92	2728/2974	3019	11.08
	CMV_RNA3	AY429437.1	–	97	2153/2212	2269	52.4

*BCMNV, Bean common mosaic necrosis virus; PvEV, Phaseolus vulgaris Endornavirus; CMV, Cucumber mosaic virus RNAs 1, 2, and 3; Length, nucleotides*.

### Bean Common Mosaic Necrosis Virus: Identification and Phylogenetics

We detected BCMNV in five samples out of the 12 obtained from the Nairobi and Kirinyaga regions of Central Kenya; specifically, in samples 3, and 5 to 9 (S3, S5-S9) (Table 3). The BCMNV sequence length was consistent with what has been reported previously (Larsen et al., 2011; Feng et al., 2017). The deduced amino acid sequences encoded by the BCMNV sequences revealed a putative polyprotein of 3,071 amino acids (aa) with an average predicted molecular mass of 350.8 kDa, predicted to yield proteolysis products typical for potyviruses (Ivanov et al., 2014): P1 (317 aa); HC-Pro (457 aa); P3 (347 aa); 6K1 (52 aa); CI (634 aa); 6K2 (53 aa); VPg (190 aa); NIa-Pro (243 aa); Nlb (517 aa); and CP (261 aa). The P3N-PIPO movement protein is translated from a viral RNA template generated by transcriptional slippage (Chung et al., 2008; Olspert et al., 2015, 2016). The PIPO read-through sequence encoded an amino acid sequence of *c*. 9 kDa with the predicted P3N-PIPO product having an estimated size of ~26 kDa. The 5′ untranslated region comprised 149–190 bases and the 3′ untranslated region consisted of 242 bases excluding the poly(A) tail. The 3′ untranslated regions were more conserved whereas the 5′ untranslated regions were more variable. Samples S2, S3, S5, S10, and S11 shared a BCMNV sequence that was similar to that of BCMNV strain 1755b (GenBank accession KY659305.1) whereas, S4, S8, and S12 all contained BCMNV sequence most similar to that of BCMNV strain NL8-CA99 (GenBank accession KY659304.1) (Table 3). The sample S6 contained a BCMNV sequence that was similar to the BCMNV strain NL3 (GenBank accession AY282577.1), whereas S9 contained a BCMNV sequence that was similar to the BCMNV strain TM70 (GenBank accession KX302007.1). The nucleotide and predicted amino acid sequences of the novel BCMNV strains found in the 11 samples (S2 to S12) were highly similar to those of BCMNV strains 1755b, NL8-CA99, NL3, and TM70 in the NCBI database, with percentage nucleotide similarities ranging from 97 to 99% (Table 3). The genome sequences of the BCMNV strains detected in our samples were all consistent with the lengths of the genomes of BCMNV strains reported by Larsen et al. (2011).

After comparison with the BCMNV strain TN-1 (HQ229995.1), we found that the N-termini of the CPs encoded by the identified near-full-length BCMNV strains that show similarity to BCMNV strain TN-1 and TM-70 detected in S3, S4, S5, S6, S8, and S9 had the aspartate-alanine-glycine (DAG) motif located in the CP position 2,819–2,821 of the polyprotein, which plays a role in conditioning potyvirus transmission by aphids (Atreya et al., 1991; Blanc et al., 1997). Additionally, the proline-threonine-lysine (PTK) motif, which is also required for aphid-mediated transmission (Atreya et al., 1991; Blanc et al., 1997; Peng et al., 1998; Larsen et al., 2011), of the HC-Pro proteins encoded by the identified BCMNV sequences was conserved in all our samples. The PTK motif was located at residues 626–628 of the polyprotein.

**Figure 2:**
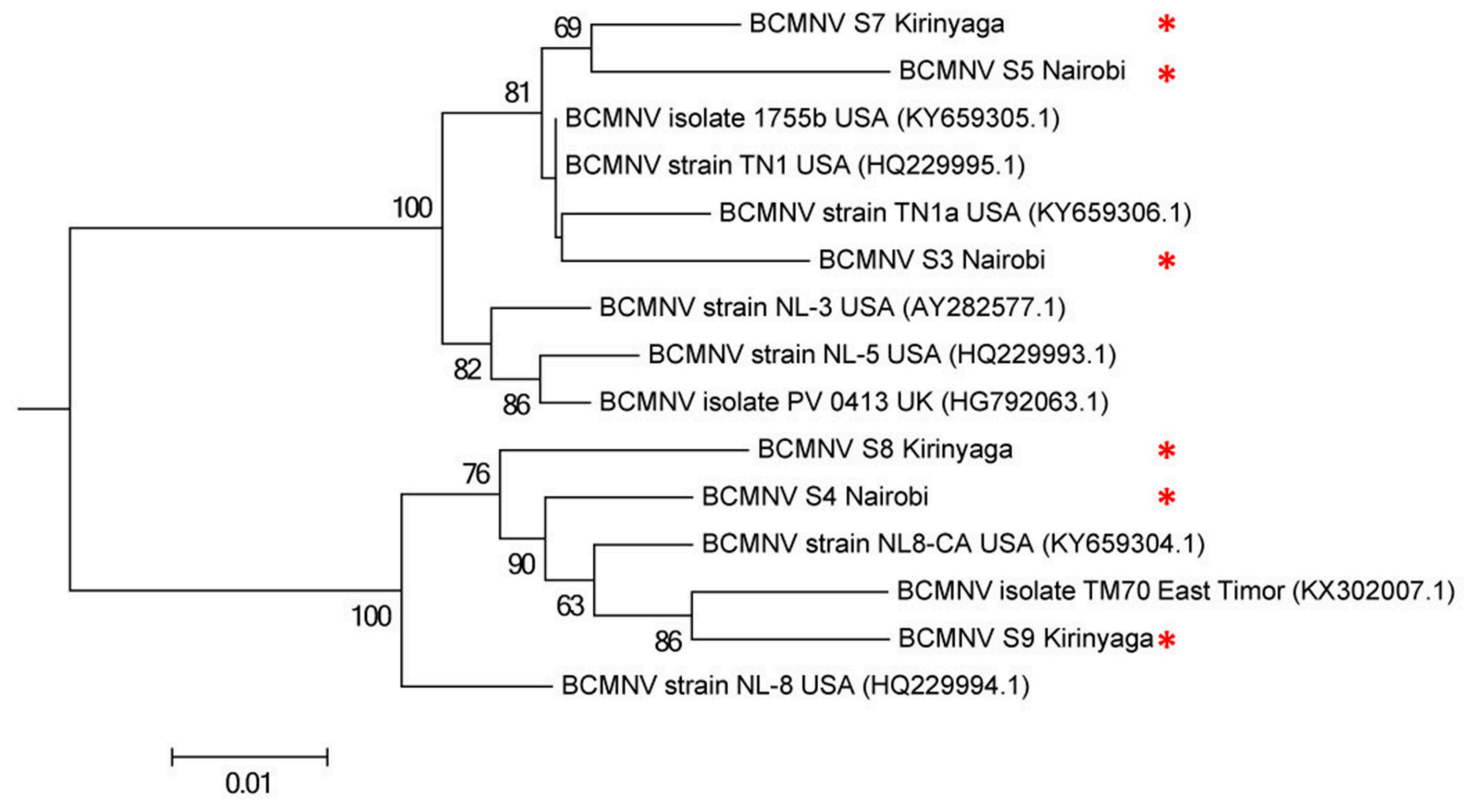
Phylogenetic analysis of six bean common mosaic necrosis virus (BCMNV) isolates from Kabete (Nairobi University Research Farm) and farms in Kirinyaga County with other BCMNV isolates and strains. The phylogeny was based on aligning 312 amino acid bases of the P1 protein. The evolutionary history was inferred by using the Maximum likelihood method based on the Jones-Taylor-Thornton matrix based model with 1,000 bootstraps in MEGA version 6. Viral isolates identified in this study are indicated by ^*^.

We compared the predicted amino acid sequences of the viral proteins P1, HC-Pro, P3, 6K1, CI, and CP in our samples with those of the BCMNV strains TN-1 and TM-70 and found differences in some samples. The P1 and P3 proteins of S3, S5, and S6 were the least conserved at the amino acid level, whereas 6K1 and CP were most conserved (Tables 4, 5). As the P1 protein may be involved in BCMNV host specificity (Larsen et al., 2011) we conducted phylogenetic analysis using P1 amino acid sequences of BCMNV strains that were similar to BCMNV strains TN-1 and TM-70 isolated from samples S3, S4, S5, S6, S8, and S9, and the P1 proteins of BCMNV strains TN-1 (U37076), NL-5 (HQ229993), NL-3 (Z17203), NL-8 (HQ229994), and TM-70 (KX302007.1) based on the Jones-Taylor-Thornton matrix-based model at 1,000 bootstrap (Jones et al., 1992) (Figure 2). The P1 proteins of S3, S5, and S7 were closely related and in the same clade as BCMNV strain TN-1 (HQ229995.1) and another USA isolate (KY659305.1) (Figure 2). BCMNV strains NL-5 and NL-3 formed a separate clade and were not closely related to any of the BCMNV strains we isolated from bean leaves (Figure 2). BCMNV strains isolated from S8, S4, to S9 were in the same clade as BCMNV strain TM-70 although S9 was more closely related to BCMNV strain TM-70 than S8 and S4 (Figure 2). This is further supported by the differences in amino acid sequences in these BCMNV strains (Tables 4, 5). We have designated these isolates as BCMNV_*Beca1*, BCMNV_*Beca2*, BCMNV_*Beca3*, BCMNV_*Beca4*, BCMNV_*Beca5*, and BCMNV_*Beca6* for S3, S4, S5, S6, S8, and S9, with GenBank accession numbers MH169564, MH169565, MH169566, MH169567, MH169568, MH169569, and MH169563, respectively.

**Table 4:** Summary of amino acid sequence comparisons between BCMNV strain TN1 and (S3, S4, S5, S6, S8, S9).

**Gene**	**% Identity of amino acids**						**Substitution positions**					
	**S3**	**S4**	**S5**	**S6**	**S8**	**S9**	**S3**	**S4**	**S5**	**S6**	**S8**	**S9**
P1	98	93	97	99	92	92	21,62,67,199,251	16,19,26,27,45,52,65,109,115,131,136,139,153,183,187,227,238,240,249,251,260	33,138,175,209,243,254,265	243	11,26,27,37,45,65,109,115,120,131,136,139,153,183,187,199,227,238,240,244,249,259,260	26,27,45,65,109,115,116,131,132,134,136,139,147149,153,183,184,187,204,227,238,240,249,259,260
HC-Pro	99	98	99	99	97	97	**149**,**204**	20,24,57,72,98,112,**149**,151,**204**	89,98,**149**,**204**	98,**149**,**204**	20,24,57,72,98,112,**149**,151,**204**,236	20,24,57,72,98,103,112,**149**,151,**204**,236
P3	99	95	99	99	94	94	**106**	22,92,**106**,130,217,219,220,221,230,234,240,253,279	**106**,193	**106**	22,**106**,130,143,164,198,217,219,220,221,230,234,240,253,279,282,285,292,302,307	22,**106**,130,143,164,198,217,219,220,221,230,234,240,253,279,282,285,292,307
6K1	96	96	98	98	96	96	23,48	23,41	23	23	41,45	23,41
CI	99	99	99	99	99	99	**475**,**556**,**616**	416,458,467,**475**,**556**,582,**616**	**475**,**556**,**616**	458,467,**475**,521,528,**556**,571,**616**	416,458,467,**475**,**556**,**616**	416,458,467,**475**,**556**,**616**
CP	99	99	99	99	99	99	**122**	35,**122**	**122**	**122**	78,**122**	35,**122**

*Amino acid positions in bold indicate that the substitution position was consistent in isolates in each sample compared to BCMNV strain TN1*.

**Table 5:** Summary of amino acid sequence comparisons between BCMNV strain TM-70 and S3, S4, S5, S6, S8, S9.

**Gene**	**% Identity of amino acids**						**Substitution positions**					
	**S3**	**S4**	**S5**	**S6**	**S8**	**S9**	**S3**	**S4**	**S5**	**S6**	**S8**	**S9**
P1	91	96	89	91	95	97	**10**,21,26,27,45,65,**68**,110, 115,131,134, 136,139,147, 149,153,183,187,199,**225**,227,238,240,249,251,260,**292**	**10**,16,18,52,**68**,134,147,149,**225**,**292**	**10**,26,27,33,45,65,**68**,110, 115,131,134,136,138,139,147,149,153,175,183,187,209,225,227,238,240,243,249,251,254,260,265,**292**	**10**,26,27,45,62,65,67,**68**,110,115,131,134,136,139,147,149,153,183,187,**225**,227,238,240,243,249,251,260,**292**	**10**,11,37,**68**,120,134,147,149,199,204,**225**,240,2244,**292**	**10,68**,116,132,184,**292**,**292**
HC-Pro	97	99	97	98	99	99	20,24,57,72,98,103,**111**,112,151,237	103,**111**,237	20,24,57,72,89,103,**111**,112,151,237	20,24,57,72,103,**111**,112	103,**111**	103,**111**
P3	94	98	94	94	99	99	**11**,32,130,133,164,198,217,219,220,221,230,234,240,263,279,282,285,292,307	**11**,92,284285,292,307	**11**,32,130,133,164,193,198,217,219,220,221,230,234,240,263,279,282,285,292,307	**11**,32,130,133,164,198,217,219,220,221,230,234,240,263,279,282,285,292,307	**11**,279,302	**11**,292
6K1	96	100	98	98	96	100	44,18		41	41	23,45	
CI	98	98	98	97	98	98	**6,9,54,282**,**289,399**,458,467,**533**,582	**6**,**9**,**54**,68,**282**, **289**,**399**,416,**533**	**6**,**9**,**54**,**282,289,399**,458,467,**533**,582	**6,9,54**,254,267,274,**282**,**289,399**,521,528,533,571,583	**6,9,54**,68,214,**282,289,399**,329,416,**533**,582	**6**,**9**,**54**,68, **282,289,399**, 416,**533**,582
CP	99	99	99	99	99	99	35		35	35	35,78	

*Amino acid positions in bold indicate that the substitution position was consistent in isolates in each sample compared to BCMNV strain TM-70*.

### *Phaseolus vulgaris Alphaendornavirus* (PvEV): Identification and Phylogenetics

Plant-infecting endornavirus genomes contain a single open reading frame that encodes a protein containing conserved domains for RNA helicase (Hel-1), UDP-glycosyltransferase (UGT), methyltransferase (MTR), and RNA-dependent RNA polymerase (RdRp) active sites (Okada et al., 2011). *De novo* assembly generated short PvEV1 contigs from samples S10, S11, and S12. To obtain longer sequences for PvEV1, we pooled the reads obtained from the five contigs obtained from the Kirinyaga samples i.e., S10, S11, and S12 (Table 6) and assembled a consensus sequence which encodes a putative 4,318 aa polyprotein. This polyprotein represented 96% aa sequence coverage when compared to the sequence of PvEV1 reported by Okada et al. (2013) (Genbank ID AB719398). The PvEV2 contig was 4,532 aa, which represented 93.4% sequence coverage when compared to the polyprotein of AB719398.1 reported by Okada et al. (2013) (Table 6). As previously reported (Okada et al., 2013; Nordenstedt et al., 2017), a capsular polysaccharide synthase (CPS)-like domain was found only in the PvEV1-encoded polyprotein, whereas an MTR domain was found only in the PvEV2-encoded polyprotein sequence (Supplemental Figures S2, S3). The UGT domain was located between the Hel-1 and RdRp domains. In PvEV2 found in Kenya, the UGT domain was 25 amino acids shorter than in the sequence of the isolate reported by Okada et al. (2013). We compared the percentage identity of conserved domains between the PvEV1 in the Kenyan samples and isolates reported by Okada et al. (2013). With reference to similar domains reported by Okada et al. (2013), the sequences of the Hel-1, CPS-like, UGT, and RdRp domains in the Kenyan PvEV1 isolate shared 98.8, 98.9, and 98.6%, identity, respectively, with those reported previously (Okada et al., 2013). In the Kenyan PvEV1 isolate the RdRp domain, for which we obtained 66.8% of the domain sequence, shared 98.7% identity with the sequences of the RdRp domain of the PvEV1 polyprotein reported by Okada et al. (2013) (GenBank accession number AB719397.1). Similar analysis using sequences of the Kenyan PvEV2 isolate showed that the MTR, Hel-1, and the UGT domains shared 99.5, 98.1, and 97.8% identity respectively, with those reported previously (Okada et al., 2013) (GenBank accession number AB719398.1). The sequences of the RdRp domain in the Kenyan PvEV2 isolate, for which we obtained 55.3% of the domain, shared 99.5% identity with the RdRp domain in the PvEV2 isolate reported by Okada et al. (2013). Notably, analysis of the Hel-1 sequence in the Kenyan isolate of PvEV1 revealed amino acid substitutions when compared to their corresponding sequences in Okada et al. (2013) (GenBank accession number AB719397). The Kenyan PvEV1 Hel-1, which lies at 1,368–1,618 aa had several substitutions, a G at position 1,454 was substituted for an S, a V at position 1,477 was substituted for an M, an A at position 1,571 was substituted for a T, a G at position 1,454 was substituted by S, whereas, an A at position 1,571 was substituted by T.

**Table 6:** The coverage and average read depth of *Phaseolus vulgaris* Endornavirus (PvEV) 1 and 2 before sample pooling.

**Sample pool**	**Reference**	**Virus**	**Coverage**	**Average depth**	**No. of contigs**	**Length of contigs (bp)**
Kirinyaga	AB719397.1	PvEV1	59.9%	8.1	5	184–8339
Nairobi	AB719397.1	PvEV1	13.7%	3.7	13	362–1915
Nairobi	MF281671.1	PvEV2	35.8%	5.2	4	1253–5305

**Figure 3:**
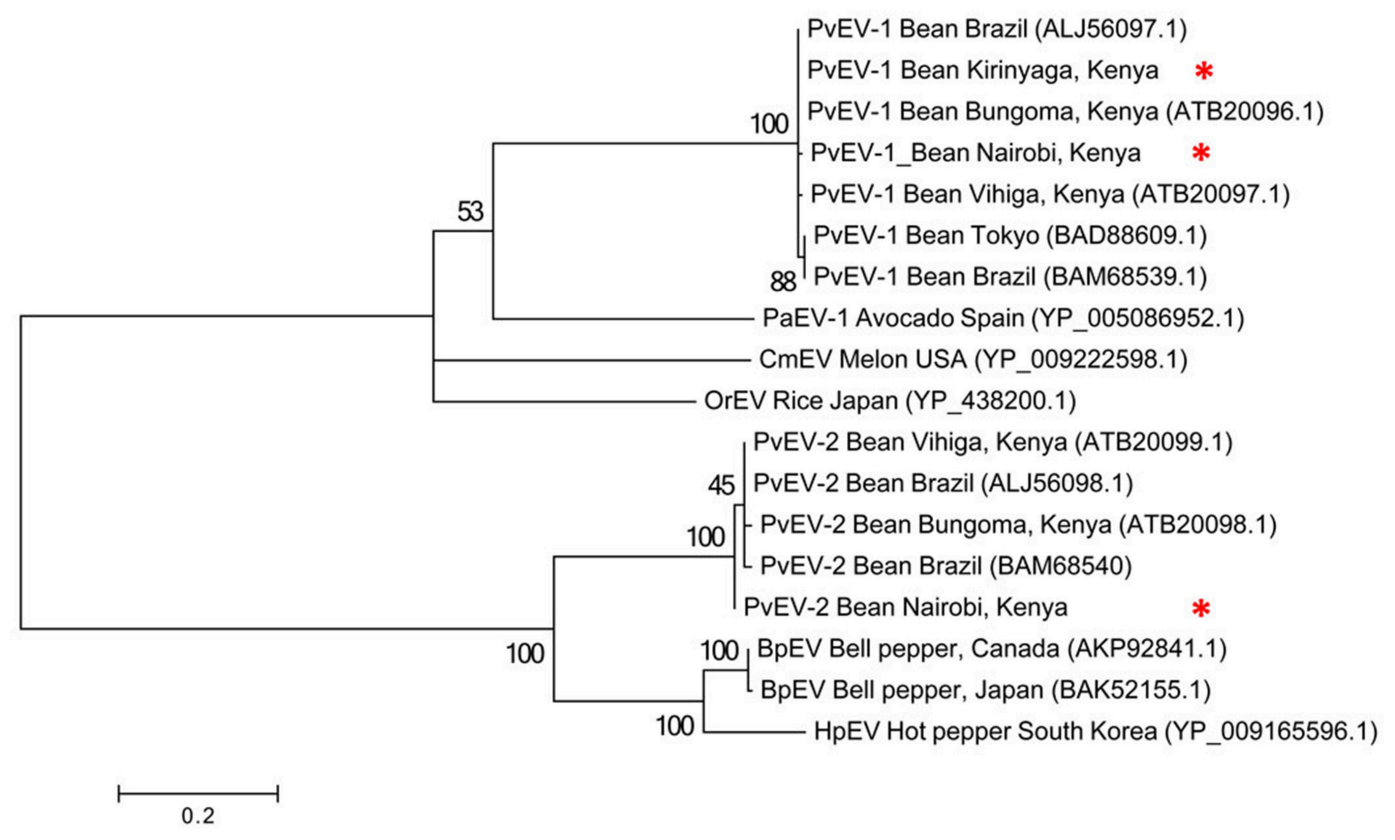
Phylogenetic analysis of *Phaseolus vulgaris* alphaendornavirus 1 (PvEV1) and PvEV2 isolates from farms in Kirinyaga County and Nairobi University Research Farm, Kabete with other endornavirus strains using the viral helicase (Hel-1) protein. The phylogeny was based on aligning 235 amino acids of the viral Hel-1 helicase sequence. The evolutionary history was inferred by the Maximum Likelihood Method based on the WAG substitution model (Whelan and Goldman, 2001) with 1,000 bootstraps in MEGA version 6. PaEv, *Persea americana* alphaendornavirus 1; CmEV, *Cucumis melo* alphaendornavirus; OrEV, *Oryza sativa* alphaendornavirus; BpEV, bell pepper alphaendornavirus; HpEV, hot pepper alphaendornavirus. Endornavirus sequences identified in this study are indicated by ^*^.

To determine the evolutionary relationships among the Kenyan PvEV1 and PvEV2 isolates (GenBank Accession Numbers MH567335 and MH567336, for PvEV1 and PvEV2 isolates detected in S1; MH567338 and MH567339 for PvEV1 and PvEV2 isolates detected in S3; MH567346 for PvEV1 isolate detected in S11; and MH567351 for PvEV1 isolate detected in S12) we constructed a phylogenetic tree using the Hel-1 domain of the 4,318 aa PvEV1 polyprotein obtained by pooling contigs obtained from the Kirinyaga samples i.e., samples S10, S11, S12. The analysis included isolates obtained from Brazil, Japan, Spain, USA, and Kenya. The host ranges associated with these sequences were diverse and included common bean, avocado, melon, rice, bell pepper, and hot pepper (Figure 3). The analysis divided the PvEV1 and PvEV2 into two distantly related groups (Figure 3). The first group contained four sub-groups, one of which included two isolates from Brazil, one from Tokyo and four isolates from Kenya i.e., Bungoma, Vihiga, and our isolates from Kirinyaga and Kabete, Nairobi. Subgroups two to four contained one isolate each, *Persea americana* alphaendornavirus 1 (PaEV1) of avocado from Spain, *Cucumis melo* alphaendornavirus (CmEV) of melon from the USA, *Oryza sativa* alphaendornavirus (OrEV) of rice from Japan respectively. The second group, which included the PvEV2 isolates contained four subgroups. The first subgroup contained two isolates each from Kenya and Brazil, the second sub group contained the PvEV2 isolates detected in Nairobi in this study, the third sub group contained Bell pepper alphaendornavirus isolates from Canada and Japan, and the last sub group contained hot pepper alphaendornavirus isolates from South Korea (Figure 3).

### CMV Infecting Beans in Central Kenya Is Most Likely of Asian Origin

CMV was found in samples S10, S11, and S12 all of which were collected in Kirinyaga. The CMV genome consists of three segments: RNA1, RNA2, and RNA3 (Palukaitis and García-Arenal, 2003; Jacquemond, 2012). RNA1 encodes the 1a helicase/methyltransferase protein, RNA2 has two ORFs encoding the 2a RNA-dependent RNA polymerase and the 2b RNA silencing suppressor, and RNA3 encodes the 3a cell-to-cell movement protein and CP (Palukaitis and García-Arenal, 2003; Takeshita et al., 2009; Jacquemond, 2012). Reads for the three genomic RNAs were assembled with reference genomes to obtain the full-length sequences of the Kenyan CMV isolates. RNA1 was 3,296 nt, RNA2 was 2,924 nt, and RNA3 was 2,139 nt. The GenBank Accession Numbers of CMV RNA1, RNA2, and RNA3 of S10 were MH567342, MH567343, and MH567344. Those for the three genomic RNAs in S11 were MH567347, MH567348, and MH567349, and those for S12 were MH567350, MH567351, and MH567354. These sizes are consistent with those reported elsewhere (Thompson et al., 2015).

**Figure 4:**
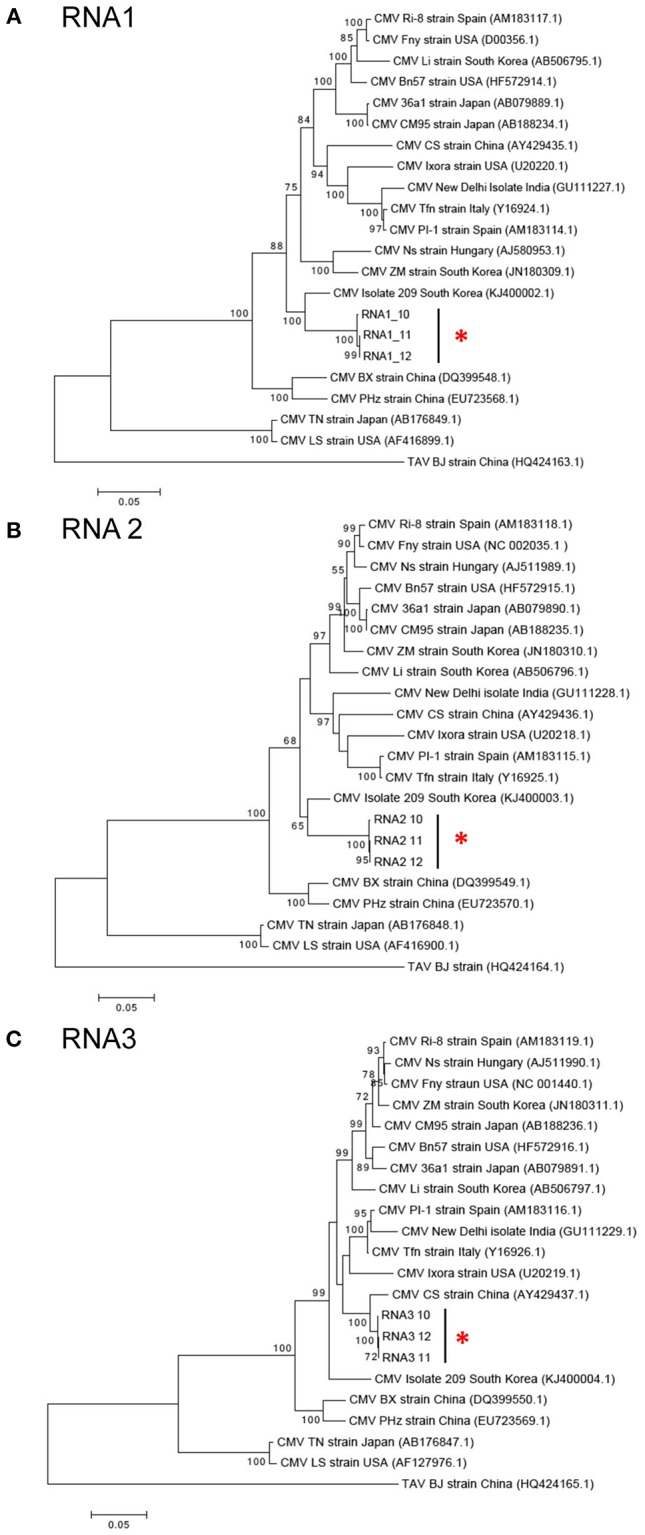
**(A–C)** Phylogenetic analysis of cucumber mosaic virus (CMV) RNAs 1, 2, and 3 of the Kenyan strain with other CMV strains and isolates from diverse plant hosts. The phylogeny was based on aligning 2,994 nucleotide bases of the RNA1, 2,079 nucleotide bases of the RNA2 and for RNA3, an alignment of 657 nucleotide bases of the coat protein gene. The evolutionary history was inferred by the Maximum likelihood method based on the Tamura Nei model with 1,000 bootstraps in MEGA version 6. Placement of the three isolates from samples obtained at Kirinyaga is indicated by asterisks.

The sequences for RNAs 1–3 of the Kirinyaga isolates all cluster together with each other, indicating that they belong to the same strain of CMV (Figure 4). CMV can be subdivided into two main phylogenetic Subgroups I and II, sharing 70–75% nucleotide sequence identity making them taxonomically separate species. Subgroup I can be further subdivided into IA and IB, with IB being predominantly Asian in distribution (Palukaitis and García-Arenal, 2003; Jacquemond, 2012; Kim et al., 2014; Thompson et al., 2015). BLAST search and neighbor-joining phylogenetic analysis showed that the genomic sequences of RNAs 1, 2 of the CMV isolated in central Kenya most closely associate with corresponding CMV RNA segments of South Korean origin, sharing up to 94 and 92% nucleotide sequence identity respectively, that belong to CMV Subgroup IA. Curiously, however, the RNA 3 sequences of the Kirinyaga isolates shared 97% nucleotide sequence identity with the RNA 3 sequence of a Subgroup IB CMV strain of Chinese origin (Table 3; Figure 4). We conclude that the Kirinyaga CMV isolates represent a strain that has Asian origins, and which has arisen from reassortment between genomic RNA segments of South Korean and Chinese CMV strains.

### Verification of the Presence of Viruses by RT-PCR

The presence of virus sequences present in samples is listed in Supplemental Table S2. We authenticated the presence of BCMNV, CMV, and PvEV in the samples (Supplemental Figure S4). BCMNV and CMV are both transmitted non-persistently by aphids (with *Aphis fabae* being the mostly likely vector in this case due to its prevalence in bean plots: Wamonje et al., 2017) whilst PvEV1 and 2 are vertically transmitted (Okada et al., 2011). Out of the twelve samples analyzed, samples S1 and S3 contained sequence signatures for both BCMNV and PvEV1 and 2. Samples S10, S11, and S12 contained sequence signatures for BCMNV, CMV and PvEV1 (Table 3). Samples S1 and S3 were obtained from the Kirinyaga region while samples S10, S11, and S12 were obtained from the Nairobi region.

To verify our findings that the CMV strain infecting common bean (*Phaseolus vulgaris*) in central Kenya was a Subgroup IB strain likely to be of Asian origin, we conducted RT-PCR using primers designed to amplify the coat protein sequence of CMV (Kwon et al., 2016). The RT-PCR reactions produced a single amplification product from virus samples obtained from Kirinyaga but not from those obtained from Nairobi. RT-PCR product bands were excised from an agarose gel, purified and sequenced using the Sanger method. BLAST results confirmed that the CMV strain (tentatively named CMV-Kirinyaga) isolated from symptomatic common beans in central Kenya was most likely of Asian origin. Similarly, by using RT-PCR, sequencing and BLAST analysis, we confirmed the presence of BCMNV and PvEV1 (Supplemental Figure S4).

## Discussion

Plant virus metagenomics has the capacity to detect viruses either as single agents or as components in complex infections and can reveal the presence of novel or unsuspected agents (MacDiarmid et al., 2013: Roossinck et al., 2015). In our study, we detected BCMNV, CMV, and PvEV in symptomatic common bean leaves obtained from farms in central Kenya. The discovery of BCMNV in bean-growing regions of Kenya concurs with earlier studies reporting the presence of this virus in eastern Africa (Sengooba et al., 1997; Mangeni et al., 2014; Worrall et al., 2015; Mwaipopo et al., 2017). The BCMNV isolates we detected in Kenya have the DAG motif. This motif in association with the highly conserved PTK motif of the potyviral HC-Pro protein are required for aphid transmission. Similarly, the BCMNV strain TN1 reported by Larsen et al. (2011) has the DAG motif unlike the case in BCMNV strain NL-5 and BCMNV strain PV 0413 (GenBank accession number HG792063) in which the DAG motif is substituted with a NAG motif.

Interestingly, we did not detect BCMV in any samples. In recent years, there have been efforts to deploy bean varieties with the dominant *I* gene due to their resistance to many strains of BCMV (Worrall et al., 2015). However, the presence of BCMNV limits the usefulness of this genetic resistance to BCMV since in these plants, BCMNV induces “black root” disease, characterized by spreading necrosis caused by an abortive hypersensitive response conditioned by the *I* gene (Worrall et al., 2015). It is conceivable that use of lines possessing the *I* gene may have decreased the incidence of BCMV while allowing BCMNV to become the dominant bean-infecting potyvirus. As many bean-growing smallholder farmers store seed and plant mixtures of bean with varying genotypes, it was not clear if farmers were growing plants harboring the *I* gene.

CMV is the type species of the genus *Cucumovirus* and is one of the most common plant viruses of major agricultural significance (Palukaitis and García-Arenal, 2003; Jacquemond, 2012). CMV infects over 1,200 species of plants, including legumes such as common bean, cowpea, and soybean (Kim and Palukaitis, 1997; Morales, 2006; Gildow et al., 2008; Jacquemond, 2012). Unlike other viruses that infect bean, such as BCMV where resistance is well-characterized (Worrall et al., 2015), obtaining effective genetic resistance to CMV in bean has proved to be problematic (Griffiths, 2004; Jacquemond, 2012). For example, only one dominant resistance gene conditioning a response to CMV has been isolated from common bean (*RT4-4*), which was discovered due to its up-regulation by a geminivirus (Seo et al., 2006). Although this putative resistance gene conditioned systemic necrosis in response to infection by pepper, cucurbit, and tomato isolates of CMV in *RT4-4*-transgenic *Nicotiana benthamiana*, which might be considered to be an abortive hypersensitive resistance reaction, the gene conferred no such response to systemic infection either in these transgenic plants or in non-transgenic common bean against a bean-specific CMV isolate (Seo et al., 2006; Jacquemond, 2012). Hence, in many parts of the world CMV continues to be an important pathogen of common bean (Morales, 2006) and can cause serious epidemics (for example, see Thompson et al., 2015). However, up to now CMV has not been viewed as a major threat in East or Central Africa, whereas BCMV or BCMNV have been thought to be the main viral challenges to common bean cultivation (Worrall et al., 2015; Mwaipopo et al., 2017).

PvEV1 and 2 are transmitted vertically with no evidence for horizontal transmission (Roossinck et al., 2015). In contrast, both BCMNV and CMV are non-persistently transmitted by aphids but are also seed transmissible in some bean varieties (Morales, 2006; Worrall et al., 2015). A number of studies in plant disease systems have reported parasite-induced changes in host phenotypes that appear conducive to vector transmission (Mauck et al., 2012; Palukaitis et al., 2013; Salvaudon et al., 2013; Groen et al., 2017; Tungadi et al., 2017). However, further study will be required to determine how these virus-induced phenotypes might affect transmission under field conditions, or if the presence of endornaviruses affects vector-borne or seed transmission of CMV or BCMNV in bean.

Vertical transmission of endornaviruses occurs through seeds, pollen or (in the case of fungus- or oomycte-infecting endornaviruses) fungal spores whereas horizontal transmission by contact or by insect or other vectors has not been reported (Fukuhara et al., 2006). Although effects on male sterility were documented in *Vicia faba* in an early report (Grill and Garger, 1981), endornaviruses in general do not appear to be pathogenic. However, some subtle and apparently non-harmful effects on common bean physiology and development have been reported for PvE1 and 2, which can affect pigment composition, seed size and germination characteristics (Okada et al., 2013; Khankhum and Valverde, 2018). It will be interesting in future work to determine how the presence of endornaviruses affects the far less subtle effects of BCMNV and CMV on host physiology, development, and on host-insect interactions. Certainly, the apparent tendency of endornaviruses to co-evolve with their hosts (Safari and Roossinck, 2018) is suggestive that these viruses and their hosts might derive some mutual benefit so that their association is maintained over many generations.

Kenyan common bean genotypes are predominantly of Andean origin (Asfaw et al., 2009) and the detection of endornaviruses in these genotypes was consistent with recent findings showing that both Mesoamerican and Andean bean lineages harbor PvEV sequences (Nordenstedt et al., 2017). This is in contrast with an earlier report suggesting genotypes of Andean origin do not harbor PvEV (Khankhum et al., 2015). Our findings that PvEV1 and 2 occur in Kenya, combined with the findings that these viruses also occur in common bean in Tanzania (Nordenstedt et al., 2017), indicate that these endornaviruses are more widely distributed in the region's bean germplasm than previously thought.

The detection in Kenya of a reassortant bean-infecting CMV strain (CMV-Kirinyaga) comprising an RNA 3 belonging to Subgroup IB (strains of which are considered to be predominantly Asian in their distribution) and Subgroup IA RNAs 1 and 2 of likely Asian origin (Figure 4) is to the best of our knowledge the first time this has been reported in East and Central Africa. The recent report of isolates of CMV Subgroup IB strains infecting tomato in Egypt (Rabie et al., 2017) suggests that CMV Subgroup IB strains are spreading from Asia and may be a problem likely to emerge more widely in Africa. The appearance of exotic CMV strains in Africa may represent a serious threat to a number of crops. This is because of difficulties in conventional breeding of resistance to CMV in common bean and also because the wide host range of CMV includes important Eastern and Central African staples such as maize (*Zea mays*) (Palukaitis and García-Arenal, 2003) and banana (*Musa* spp.) (Singh et al., 1995). An additional risk stems from the propensity of CMV to exhibit synergy in mixed virus infections (Palukaitis and García-Arenal, 2003; Jacquemond, 2012), Interestingly, it was recently shown that the East African strains of maize chlorotic mottle virus (MCMV) that synergize with the potyvirus sugarcane mosaic virus (or a number of other viruses) to cause maize lethal necrosis disease are extremely similar to Chinese strains (Braidwood et al., 2018; Wamaitha et al., 2018). This suggests that the recent outbreak of an epidemic of maize lethal necrosis disease in East Africa was likely to have been initiated through the inadvertent importation of a Chinese strain of MCMV. Taken together with our discovery of a bean-infecting CMV strain with a likely Asian origin suggests that increased vigilance is needed to prevent accidental importation of exotic viruses that may threaten African food security.

## Availability of Data and Materials

Next generation sequencing data have been deposited to NCBI and under the following GenBank Accession Numbers: MH169563, MH169564, MH169565, MH169566, MH169567, MH169568, and MH169569.

## Author Contributions

Conceived and designed the experiments: JC, JH, AD, GM, and JM. Performed the experiments: GM, JH, JC, JN, FW, TT, and JM. Analyzed the data and wrote the paper: J-BD, JM, FW, GM, JN, MW, KK, S-KC, TT, SG, HM, AD, JC, and JH. All authors read and approved the final manuscript.

### Conflict of Interest Statement

The authors declare that the research was conducted in the absence of any commercial or financial relationships that could be construed as a potential conflict of interest.

## References

[B1] Allen D. J. (1995). An Annotated List of Diseases, Pathogens and Associated Fungi of the Common Bean (Phaseolus vulgaris) in Eastern and Southern Africa. Phytopathological Paper No. 34. Cali; Wallingford: Centro Internacional de Agricultura Tropical (CIAT); CAB International, 42.

[B2] Asfaw A. Blair M. Almekinders C. (2009). Genetic diversity and population structure of common bean (*Phaseolus vulgaris* L.) landraces from the East African highlands. Theor. Appl. Genet. 120, 1–12. 10.1007/s00122-009-1154-7 19756469

[B3] Atreya P. L. Atreya C. D. Pirone T. P. (1991). Amino acid substitutions in the coat protein result in loss of insect transmissibility of a plant virus. Proc. Natl. Acad. Sci. U.S.A. 88, 7887–7891. 10.1073/pnas.88.17.7887 1881922 PMC52409

[B4] Blanc S. Lopez-Moya J. J. Wang R. Garcia-Lampasona S. Thornbury D. W. Pirone T. P. (1997). A specific interaction between coat protein and helper component correlates with aphid transmission of a potyvirus. Virology 231, 141–147. 10.1006/viro.1997.8521 9143313

[B5] Bolger A. M. Marc L. Usadel B. (2014). Trimmomatic: a flexible trimmer for Illumina sequence data. Bioinformatics 30, 2114–2120. 10.1093/bioinformatics/btu170 24695404 PMC4103590

[B6] Braidwood L. Quito-Avila D. F. Cabanas D. Bressan A. Wangai A. Baulcombe D. C. (2018). Maize chlorotic mottle virus exhibits low divergence between differentiated regional subpopulations. Sci. Rep. 8:1173 10.1038/s41598-018-19607-4 29352173 PMC5775324

[B7] Broughton W. J. Hernández G. Blair M. Beebe S. Gepts P. Vanderleyden J. (2003). Beans (*Phaseolus* spp.) - model food legumes. Plant Soil 252, 55–128. 10.1023/A:1024146710611

[B8] Camacho C. Coulouris G. Avagyan V. Ma N. Papadopoulos J. Bealer K.. (2009). BLAST+: architecture and applications. BMC Bioinformatics.10:421. 10.1186/1471-2105-10-421 20003500 PMC2803857

[B9] Chung B. Y.-W. Miller W. A. Atkins J. F. Firth A. E. (2008). An overlapping essential gene in the *Potyviridae*. Proc. Natl. Acad. Sci. U.S.A. 105, 5897–5902. 10.1073/pnas.0800468105 18408156 PMC2311343

[B10] CIAT (2009). Common Bean: The Nearly Perfect Food. Cali: Centro Internacional de Agricultura Tropical (CIAT). Available online at: www.ciat.org/ciatinfocus/beans.htm

[B11] Feng X. Guzmán P. Myers J. R. Karasev A. V. (2017). Resistance to bean common mosaic necrosis virus conferred by the *bc-1* gene affects systemic spread of the virus in common bean. Phytopathology 107, 893–900. 10.1094/PHYTO-01-17-0013-R 28475025

[B12] Feng X. Poplawsky A. R. Nikolaeva O. V. Myers J. R. Karasev A. V. (2014). Recombinants of bean common mosaic virus (BCMV) and genetic determinants of BCMV involved in overcoming resistance in common bean. Phytopathology 104, 786–793. 10.1094/PHYTO-08-13-0243-R 24915430

[B13] Fredslund J. Schauser L. Madsen L. H. Sandal N. Stougaard J. (2005). PriFi: using a multiple alignment of related sequences to find primers for amplification of homologs. Nucleic Acids Res. 33, W516–W520. 10.1093/nar/gki425. 15980525 PMC1160186

[B14] Fukuhara T. Koga R. Aoki N. Yuki C. Yamamoto N. Oyama N.. (2006). The wide distribution of endornaviruses, large double-stranded RNA replicons with plasmid-like properties. Arch. Virol. 151, 995–1002. 10.1007/s00705-005-0688-5 16341944

[B15] Gildow F. E. Shah D. A. Sackett W. M. Butzler T. Nault B. A. Fleischer S. J. (2008). Transmission efficiency of *Cucumber mosaic virus* by aphids associated with virus epidemics in snap bean. Phytopathology 98, 1233–1241. 10.1094/PHYTO-98-11-1233 18943413

[B16] Griffiths P. (2004). Breeding snap beans for cucumber mosaic virus (CMV) resistance. Hortscience 39:869.

[B17] Grill L. K. Garger S. J. (1981). Identification and characterization of double-stranded RNA associated with cytoplasmic male sterility in *Vicia faba*. Proc. Natl. Acad. Sci. U.S.A. 78, 7043–7046. 10.1073/pnas.78.11.7043 16593124 PMC349190

[B18] Groen S. C. Wamonje F. O. Murphy A. M. Carr J. P. (2017). Engineering resistance to virus transmission. Curr. Opin. Virol. 26, 20–27. 10.1016/j.coviro.2017.07.005 28750351

[B19] Ivanov K. I. Eskelin K. Lõhmus A. Mäkinen K. (2014). Molecular and cellular mechanisms underlying potyvirus infection. J. Gen. Virol. 95, 1415–1429. 10.1099/vir.0.064220-0 24722679

[B20] Jacquemond M. (2012). Cucumber mosaic virus. Adv. Virus Res. 84, 439–504. 10.1016/B978-0-12-394314-9.00013-0 22682176

[B21] Jansa J. Bationo A. Frossard E. Rao I. M. (2011). Options for improving plant nutrition to increase common bean productivity in Africa, in Fighting Poverty in Sub-Saharan Africa: The Multiple Roles of Legumes in Integrated Soil Fertility Management, eds Bationo A. Waswa B. Okeyo J. M. Maina F. Kihara J. Mokwunye U. (Dordrecht: Springer), 201–240.

[B22] Jones D. T. Taylor W. R. Thornton J. M. (1992). The rapid generation of mutation data matrices from protein sequences. Comput. Appl. Biosci. 8, 275–282. 10.1093/bioinformatics/8.3.275 1633570

[B23] Kenya Ministry of Agriculture (2015). Economic Review of Agriculture [ERA] Central Planning and Project Monitoring Unit, 104.

[B24] Khankhum S. Valverde R. A. (2018). Physiological traits of endornavirus-infected and endornavirus-free common bean (*Phaseolus vulgaris*) cv Black Turtle Soup. Arch. Virol. 163, 1051–1056. 10.1007/s00705-018-3702-4 29307088

[B25] Khankhum S. Valverde R. A. Pastor-Corrales M. A. Osorno J. M. Sabanadzovic S. (2015). Two endornaviruses show differential infection patterns between gene pools of *Phaseolus vulgaris*. Arch. Virol. 160, 1131–1137. 10.1007/s00705-015-2335-0 25623050

[B26] Kim H. Palukaitis P. (1997). The plant defense response to cucumber mosaic virus in cowpea is elicited by the viral polymerase gene and affects virus accumulation in single cells. EMBO J. 16, 4060–4068. 10.1093/emboj/16.13.4060 9233815 PMC1170029

[B27] Kim K. Seo K. Kwak R. Kim S. Kim H. Cha J.. (2014). Molecular genetic analysis of *Cucumber mosaic virus* populations infecting pepper suggests unique patterns of evolution in Korea. Phytopathology 104, 993–1000. 10.1094/PHYTO-10-13-0275-R 25116642

[B28] Kwon S. J. Yoon J. Y. Cho I. S. Choi S. K. Choi G. S. (2016). Phylogenetic Analyses of *Pepper mild mottle virus* and *Cucumber mosaic virus* isolated from *Rorippa palustris*. Res. Plant Dis. 22, 25–31. 10.5423/RPD.2016.22.1.25

[B29] Langmead B. Steven S. (2012). Fast gapped-read alignment with Bowtie 2. Nat. Methods 9, 357–359. 10.1038/nmeth.1923 22388286 PMC3322381

[B30] Larsen R. C. Druffel K. L. Wyatt S. D. (2011). The complete nucleotide sequences of bean common mosaic necrosis virus strains NL-5, NL8 and TN-1. Arch. Virol. 156, 729–732. 10.1007/s00705-011-0945-8 21344267

[B31] Larsen R. C. Miklas P. N. Druffel K. L. Wyatt S. D. (2005). NL-3 K strain is a stable and naturally occurring interspecific recombinant derived from *Bean common mosaic necrosis virus* and *Bean common mosaic virus*. Phytopathology 95, 1037–1042. 10.1094/PHYTO-95-1037 18943301

[B32] MacDiarmid R. Rodoni B. Melcher U. Ochoa-Corona F. Roossinck M. J. (2013). Biosecurity implications of new technology and discovery in plant virus research. PLoS Pathog. 9:e1003337. 10.1371/journal.ppat.1003337 23950706 PMC3739461

[B33] Mangeni B. C. Abang M. M. Awale H. Omuse C. N. Leitch R. Arinaitwe W. (2014). Distribution and pathogenic characterization of bean common mosaic virus (BCMV) and bean common mosaic necrosis virus (BCMNV) in western Kenya. J. Agric Food Appl. Sci. 2, 308–316.

[B34] Mauck K. Bosque-Perez N. A. Eigenbrode S. D. De Moraes C. M. Mescher C. M. (2012). Transmission mechanisms shape pathogen effects on host-vector interactions: evidence from plant viruses. Funct. Ecol. 26, 1162–1175. 10.1111/j.1365-2435.2012.02026.x

[B35] Morales F. J. (2006). Common beans in Natural Resistance Mechanisms of Plants to Viruses, eds Loebenstein G. Carr J. P. (Dordrecht: Springer), 367–382.

[B36] Mucheru-Muna M. Pypers P. Mugendi D. Kung'u J. Mugwe J. Merckx R. (2010). A staggered maize-legume intercrop arrangement robustly increases crop yields and economic returns in the highlands of Central Kenya. Field Crops Res. 115, 132–139. 10.1016/j.fcr.2009.10.013

[B37] Mwaipopo B. Nchimbi-Msolla S. Njau P. Tairo F. William M. Binagwa P. (2017). Viruses infecting common bean (*Phaseolus vulgaris* L.) in Tanzania: a review on molecular characterization, detection and disease management options. Afr. J. Agric. Res. 12, 1486–1500. 10.5897/AJAR2017.12236 PMC7691756 33282144

[B38] Nordenstedt N. Marcenaro D. Chilagane D. Mwaipopo B. Rajamaki M.-L. Nchimbi-Msolla S. (2017). Pathogenic seedborne viruses are rare but *Phaseolus vulgaris* endornaviruses are common in bean varieties grown in Nicaragua and Tanzania. PLoS ONE 12:e0178242 10.1371/journal.pone.0178242 28542624 PMC5444779

[B39] Nurk S. Meleshko D. Korobeynikov A. Pevzner P. A. (2017). metaSPAdes: a new versatile metagenomic assembler. Genome Res. 27, 824–834. 10.1101/gr.213959.116 28298430 PMC5411777

[B40] Okada R. Kiyota E. Sabanadzovic S. Moriyama H Fukuhara T Saha P.. (2011). Bell pepper endornavirus: molecular and biological properties, and occurrence in the genus *Capsicum*. J. Gen. Virol. 92, 2664–2673. 10.1099/vir.0.034686-0 21775578

[B41] Okada R. Yong C. K. Valverde R. A. Sabanadzovic S. Aoki N. Hotate S.. (2013). Molecular characterization of two evolutionarily distinct endornaviruses co-infecting common bean (*Phaseolus vulgaris*). J. Gen. Virol. 94, 220–229. 10.1099/vir.0.044487-0 23015743

[B42] Olspert A. Carr J. P. Firth A. E. (2016). Mutational analysis of the *Potyviridae* transcriptional slippage site utilized for expression of the P3N-PIPO and P1N-PISPO proteins. Nucleic Acids Res. 44, 7618–7629. 10.1093/nar/gkw441 27185887 PMC5027478

[B43] Olspert A. Chung B.-W. Atkins J. F. Carr J. P. Firth A. E. (2015). Transcriptional slippage in the positive-sense RNA virus family *Potyviridae*. EMBO Rep. 16, 995–1004. 10.15252/embr.201540509 26113364 PMC4552492

[B44] Ondov B. D. Bergman N. H. Phillippy A. M. (2011). Interactive metagenomic visualization in a web browser. BMC Bioinformatics 12:385. 10.1186/1471-2105-12-385 21961884 PMC3190407

[B45] Palukaitis P. García-Arenal F. (2003). Cucumoviruses. Adv. Virus Res. 62, 241–323. 10.1016/S0065-3527(03)62005-1 14719367

[B46] Palukaitis P. Groen S. G. Carr J. P. (2013). The Rumsfeld paradox: some of the things we know that we don't know about plant virus infection. Curr. Opin. Plant Biol. 16, 513–519. 10.1016/j.pbi.2013.06.004 23820310

[B47] Peng Y. H. Kadoury D. Gal-On A. Huet H. Wang Y. Raccah B. (1998). Mutations in the HC-Pro gene of zucchini yellow mosaic potyvirus: effects on aphid transmission and binding to purified virions. J. Gen. Virol. 79, 897–904. 10.1099/0022-1317-79-4-897 9568986

[B48] Rabie M. Ratti C. Calassanzio M. Aleem E. A. Fattouh F. A. (2017). Phylogeny of Egyptian isolates of *Cucumber mosaic virus* (CMV) and *Tomato mosaic virus* (ToMV) infecting *Solanum lycopersicum*. Eur. J. Plant Pathol. 149, 219–225. 10.1007/s10658-017-1164-2.

[B49] Roossinck M. Sabanadzovic S. Okada R. Valverde R. (2011). The remarkable evolutionary history of endornaviruses. J. Gen. Virol. 92, 2674–2678. 10.1099/vir.0.034702-0 21775577

[B50] Roossinck M. J. Martin D. P. Roumagnac P. (2015). Plant virus metagenomics: advances in virus discovery. Phytopathology 105, 716–727. 10.1094/PHYTO-12-14-0356-RVW 26056847

[B51] Safari M. Roossinck M. J. (2018). Coevolution of a persistent plant virus and its pepper hosts. Mol. Plant Microbe Interact. 31, 766–776. 10.1094/MPMI-12-17-0312-R 29845896

[B52] Salvaudon L. De Moraes C. M. Mescher M. C. (2013). Outcomes of co-infection by two potyviruses: implications for the evolution of manipulative strategies. Proc. R. Soc. B 280, 2012–2959. 10.1098/rspb.2012.2959 23407835 PMC3574378

[B53] Sanger F. Nicklen S. Coulson A. R. (1977). DNA sequencing with chain-terminating inhibitors. Proc. Nat. Acad. Sci. U.S.A. 74, 5463–5467. 10.1073/pnas.74.12.5463 271968 PMC431765

[B54] Sengooba T. N. Spence N. J. Walkey D. G. A. Allen D. J. Femi Lana A. (1997). The occurrence of bean common mosaic necrosis virus in wild and forage legumes in Uganda. Plant Pathol. 46, 95–103. 10.1046/j.1365-3059.1997.d01-12.x

[B55] Seo Y. S. Rojas M. R. Lee J. Y. Lee S. W. Jeon J. S. Ronald P.. (2006). A viral resistance gene from common bean functions across plant families and is up-regulated in a non-virus-specific manner. Proc. Natl. Acad. Sci. U.S.A. 103, 11856–11861. 10.1073/pnas.0604815103 16880399 PMC1567666

[B56] Singh Z. Jones R. A. C. Jones M. G. K. (1995). Identification of cucumber mosaic virus subgroup I isolates from banana plants affected by infectious chlorosis disease using RT-PCR. Plant Dis. 79, 713–716. 10.1094/PD-79-0713

[B57] Smith L. M. Sanders J. Z. Kaiser R. J. Hughes P. Dodd C. Connell C. R.. (1986). Fluorescence detection in automated DNA sequence analysis. Nature 321, 674–679. 10.1038/321674a0 3713851

[B58] Syller J. (2012). Facilitative and antagonistic interactions between plant viruses in mixed infections. Mol. Plant Pathol. 13, 204–216. 10.1111/j.1364-3703.2011.00734.x 21726401 PMC6638836

[B59] Takeshita M. Matsuo Y. Suzuki M. Furuya N. Tsuchiya K. Takanami Y. (2009). Impact of a defective RNA 3 from cucumber mosaic virus on helper virus infection dynamics. Virology 389, 59–65. 10.1016/j.virol.2009.04.010 19427011

[B60] Tamura K. Stecher G. Peterson D. Filipski A. Kumar S. (2013). MEGA6: Molecular evolutionary genetics analysis version 6.0. Mol. Biol. Evol. 30, 2725–2729. 10.1093/molbev/mst197 24132122 PMC3840312

[B61] Thompson J. R. Langenhan J. L. Fuchs M. Perry K. L. (2015). Genotyping of *Cucumber mosaic virus* isolates in western New York State during epidemic years: Characterization of an emergent plant virus population. Virus Res. 210, 169–177. 10.1016/j.virusres.2015.07.028 26254084

[B62] Tungadi T. Groen S. C. Murphy A. M. Pate A. E. Iqbal J. Bruce T. J. A.. (2017). Cucumber mosaic virus and its 2b protein alter emission of host volatile organic compounds but not aphid vector settling in tobacco. Virol. J. 14:91. 10.1186/s12985-017-0754-0 28468686 PMC5415739

[B63] Wamaitha M. J. Nigam D. Maina S. Stomeo F. Wangai A. Njuguna J. N.. (2018). Metagenomic analysis of viruses associated with maize lethal necrosis in Kenya. Virol. J. 15:90. 10.1186/s12985-018-0999-2 29792207 PMC5966901

[B64] Wamonje F. O. Michuki G. N. Braidwood L. A. Njuguna J. Mutuku J. M. Djikeng A.. (2017). Viral metagenomics of aphids present in bean and maize plots on mixed-use farms in Kenya reveals the presence of three dicistroviruses including a novel Big Sioux River virus-like dicistrovirus. Virol. J. 14:188. 10.1186/s12985-017-0854-x 28969654 PMC5625602

[B65] Whelan S. Goldman N. (2001). A general empirical model of protein evolution derived from multiple protein families using a maximum-likelihood approach. Mol. Biol. Evol. 18, 691–699. 10.1093/oxfordjournals.molbev.a003851 11319253

[B66] Worrall E. A. Wamonje F. O. Mukeshimana G. Harvey J. J. W. Carr J. P. Mitter N. (2015). *Bean common mosaic virus* and *Bean common mosaic necrosis virus*: relationships, biology and prospects for control. Adv. Virus Res. 93, 1–46. 10.1016/bs.aivir.2015.04.002 26111585

