# Supplementary material for: Metagenomic Analysis of Plant Virus Occurrence in Common Bean (Phaseolus vulgaris) in Central Kenya
Source: Front Microbiol. 2018 Dec 7;9:2939. doi: 10.3389/fmicb.2018.02939 (PMC6293961; doi:10.3389/fmicb.2018.02939)
Supplement: Supplementary file 1 [file Data_Sheet_1.PDF]

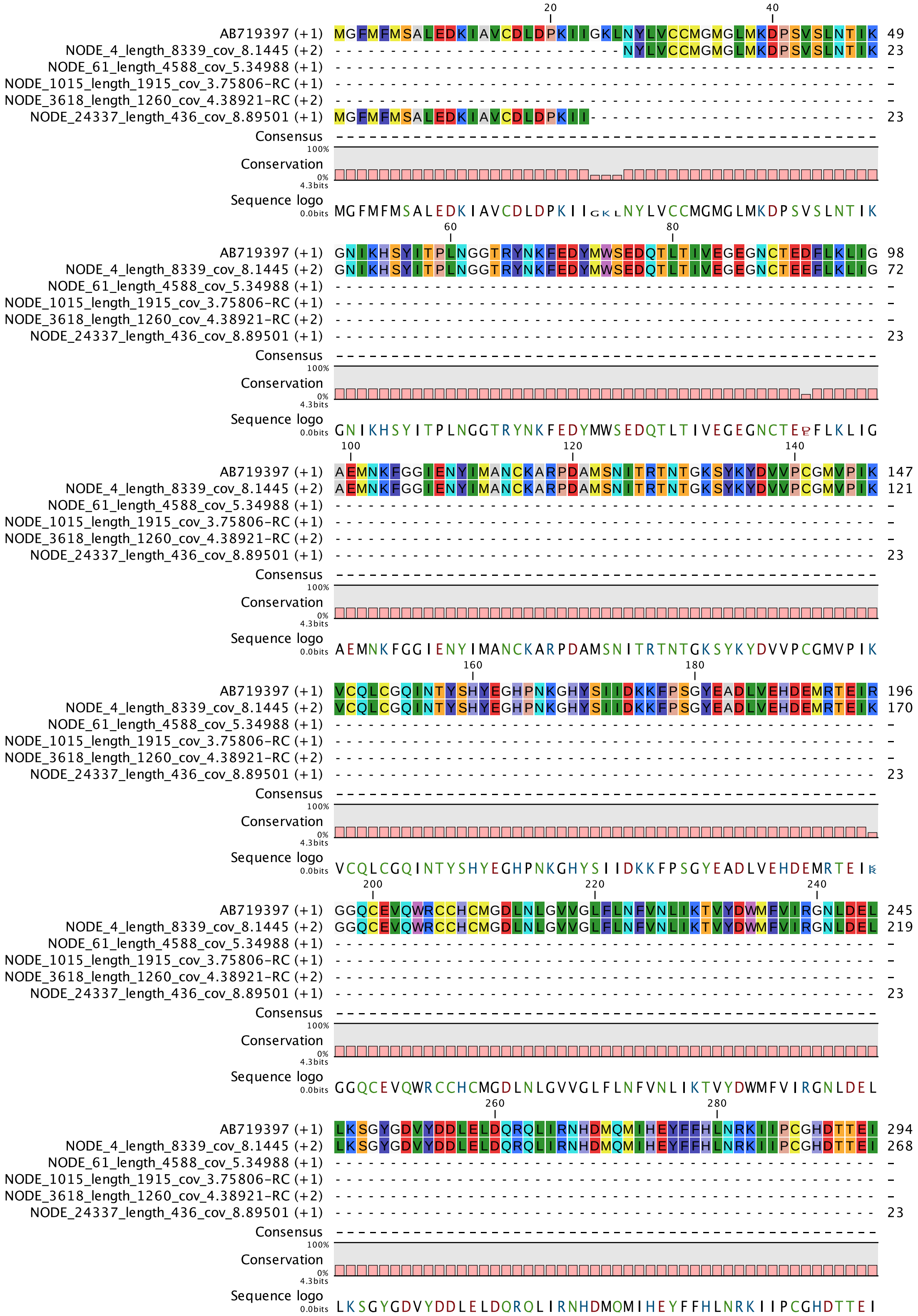
AB719397 (+1) MGFMFMSALEDKIAVCDLDPKIIGKLNYLVCCMGMGLMKDPSVSLNTIK 49
NODE_4_length_8339_cov_8.1445 (+2) --------------------------NYLVCCMGMGLMKDPSVSLNTIK 23
NODE_61_length_4588_cov_5.34988 (+1)
NODE_1015_length_1915_cov_3.75806-RC (+1)
NODE_3618_length_1260_cov_4.38921-RC (+2)
NODE_24337_length_436_cov_8.89501 (+1) MGFMFMSALEDKIAVCDLDPKII-------------------------- 23
Consensus
Conservation
Sequence logo
AB719397 (+1) GNIKHSYITPLNGGTRYNKFEDYMWSEDQTLTIVEGEGNCTEDFLKLIG 98
NODE_4_length_8339_cov_8.1445 (+2) GNIKHSYITPLNGGTRYNKFEDYMWSEDQTLTIVEGEGNCTEEFLKLIG 72
AB719397 (+1) AEMNKFGGIENYIMANCKARPDAMSNITRTNTGKSYKYDVVPCGMVPIK 147
NODE_4_length_8339_cov_8.1445 (+2) AEMNKFGGIENYIMANCKARPDAMSNITRTNTGKSYKYDVVPCGMVPIK 121
AB719397 (+1) VCQLCGQINTYSHYEGHPNKGHYSIIDKKFPSGYEADLVEHDEMRTEIR 196
NODE_4_length_8339_cov_8.1445 (+2) VCQLCGQINTYSHYEGHPNKGHYSIIDKKFPSGYEADLVEHDEMRTEIK 170
AB719397 (+1) GGQCEVQWRCCHCMGDLNLGVVGLFLNFVNLIKTVYDWMFVIRGNLDEL 245
NODE_4_length_8339_cov_8.1445 (+2) GGQCEVQWRCCHCMGDLNLGVVGLFLNFVNLIKTVYDWMFVIRGNLDEL 219
AB719397 (+1) LKSGYGDVYDDLELDQRQLIRNHDMQMIHEYFFHLNRKIIPCGHDTTEI 294
NODE_4_length_8339_cov_8.1445 (+2) LKSGYGDVYDDLELDQRQLIRNHDMQMIHEYFFHLNRKIIPCGHDTTEI 268

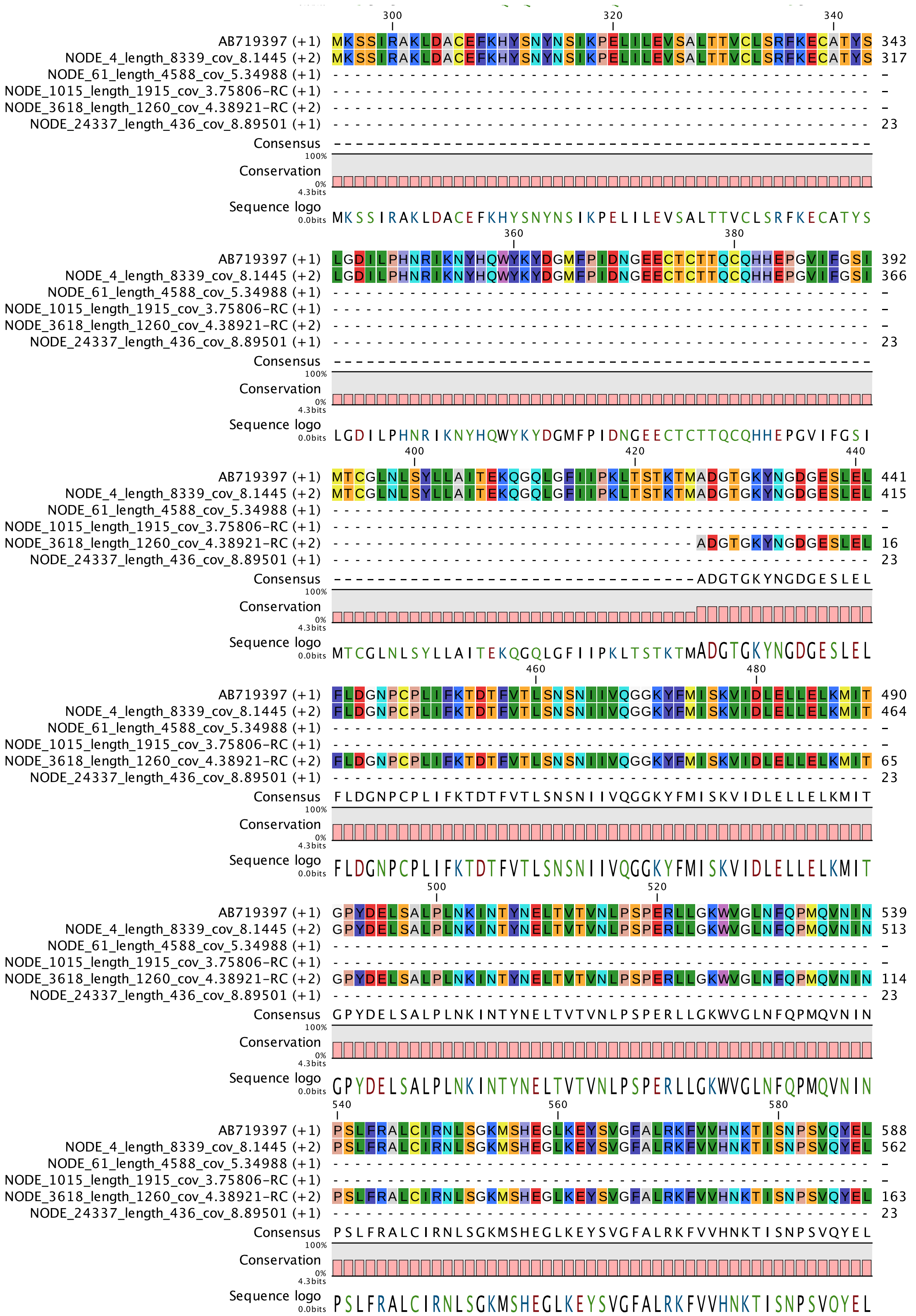
300 320 340
AB719397 (+1) MKSSIRAKLDACEFKHYSNYNSIKPELILEVSALTTVCLSRFKECATYS 343
NODE_4_length_8339_cov_8.1445 (+2) MKSSIRAKLDACEFKHYSNYNSIKPELILEVSALTTVCLSRFKECATYS 317
NODE_61_length_4588_cov_5.34988 (+1) -
NODE_1015_length_1915_cov_3.75806-RC (+1) -
NODE_3618_length_1260_cov_4.38921-RC (+2) -
NODE_24337_length_436_cov_8.89501 (+1) 23
Consensus
Conservation
Sequence logo MKSSIRAKLDACEFKHYSNYNSIKPELILEVSALTTVCLSRFKECATYS
360 380
AB719397 (+1) LGDILPHNRIKNYHQWYKYDGMFPIDNGEECTCTTQCQHHEPGVIFGSI 392
NODE_4_length_8339_cov_8.1445 (+2) LGDILPHNRIKNYHQWYKYDGMFPIDNGEECTCTTQCQHHEPGVIFGSI 366
NODE_61_length_4588_cov_5.34988 (+1) -
NODE_1015_length_1915_cov_3.75806-RC (+1) -
NODE_3618_length_1260_cov_4.38921-RC (+2) -
NODE_24337_length_436_cov_8.89501 (+1) 23
Consensus
Conservation
Sequence logo LGDILPHNRIKNYHQWYKYDGMFPIDNGEECTCTTQCQHHEPGVIFGSI
400 420 440
AB719397 (+1) MTCGLNLSYLLAITEKQGQLGFIIPKLTSTKTMADGTGKYNGDGESLEL 441
NODE_4_length_8339_cov_8.1445 (+2) MTCGLNLSYLLAITEKQGQLGFIIPKLTSTKTMADGTGKYNGDGESLEL 415
NODE_61_length_4588_cov_5.34988 (+1) -
NODE_1015_length_1915_cov_3.75806-RC (+1) -
NODE_3618_length_1260_cov_4.38921-RC (+2) ADGTGKYNGDGESLEL 16
NODE_24337_length_436_cov_8.89501 (+1) 23
Consensus ADGTGKYNGDGESLEL
Conservation
Sequence logo MTCGLNLSYLLAITEKQGQLGFIIPKLTSTKTMADGTGKYNGDGESLEL
460 480
AB719397 (+1) FLDGNPCPLIFKTDTFVTLSNSNIIVQGGKYFMISKVIDLELLELKMIT 490
NODE_4_length_8339_cov_8.1445 (+2) FLDGNPCPLIFKTDTFVTLSNSNIIVQGGKYFMISKVIDLELLELKMIT 464
NODE_61_length_4588_cov_5.34988 (+1) -
NODE_1015_length_1915_cov_3.75806-RC (+1) -
NODE_3618_length_1260_cov_4.38921-RC (+2) FLDGNPCPLIFKTDTFVTLSNSNIIVQGGKYFMISKVIDLELLELKMIT 65
NODE_24337_length_436_cov_8.89501 (+1) 23
Consensus FLDGNPCPLIFKTDTFVTLSNSNIIVQGGKYFMISKVIDLELLELKMIT
Conservation
Sequence logo FLDGNPCPLIFKTDTFVTLSNSNIIVQGGKYFMISKVIDLELLELKMIT
500 520
AB719397 (+1) GPYDELSALPLNKINTYNELTVTVNLPSPERLLGKWVGLNFQPMQVNIN 539
NODE_4_length_8339_cov_8.1445 (+2) GPYDELSALPLNKINTYNELTVTVNLPSPERLLGKWVGLNFQPMQVNIN 513
NODE_61_length_4588_cov_5.34988 (+1) -
NODE_1015_length_1915_cov_3.75806-RC (+1) -
NODE_3618_length_1260_cov_4.38921-RC (+2) GPYDELSALPLNKINTYNELTVTVNLPSPERLLGKWVGLNFQPMQVNIN 114
NODE_24337_length_436_cov_8.89501 (+1) 23
Consensus GPYDELSALPLNKINTYNELTVTVNLPSPERLLGKWVGLNFQPMQVNIN
Conservation
Sequence logo GPYDELSALPLNKINTYNELTVTVNLPSPERLLGKWVGLNFQPMQVNIN
540 560 580
AB719397 (+1) PSLFRALCIRNLSGKMSHEGLKEYSVGFALRKFVVHNKTISNPSVQYEL 588
NODE_4_length_8339_cov_8.1445 (+2) PSLFRALCIRNLSGKMSHEGLKEYSVGFALRKFVVHNKTISNPSVQYEL 562
NODE_61_length_4588_cov_5.34988 (+1) -
NODE_1015_length_1915_cov_3.75806-RC (+1) -
NODE_3618_length_1260_cov_4.38921-RC (+2) PSLFRALCIRNLSGKMSHEGLKEYSVGFALRKFVVHNKTISNPSVQYEL 163
NODE_24337_length_436_cov_8.89501 (+1) 23
Consensus PSLFRALCIRNLSGKMSHEGLKEYSVGFALRKFVVHNKTISNPSVQYEL
Conservation
Sequence logo PSLFRALCIRNLSGKMSHEGLKEYSVGFALRKFVVHNKTISNPSVQYEL

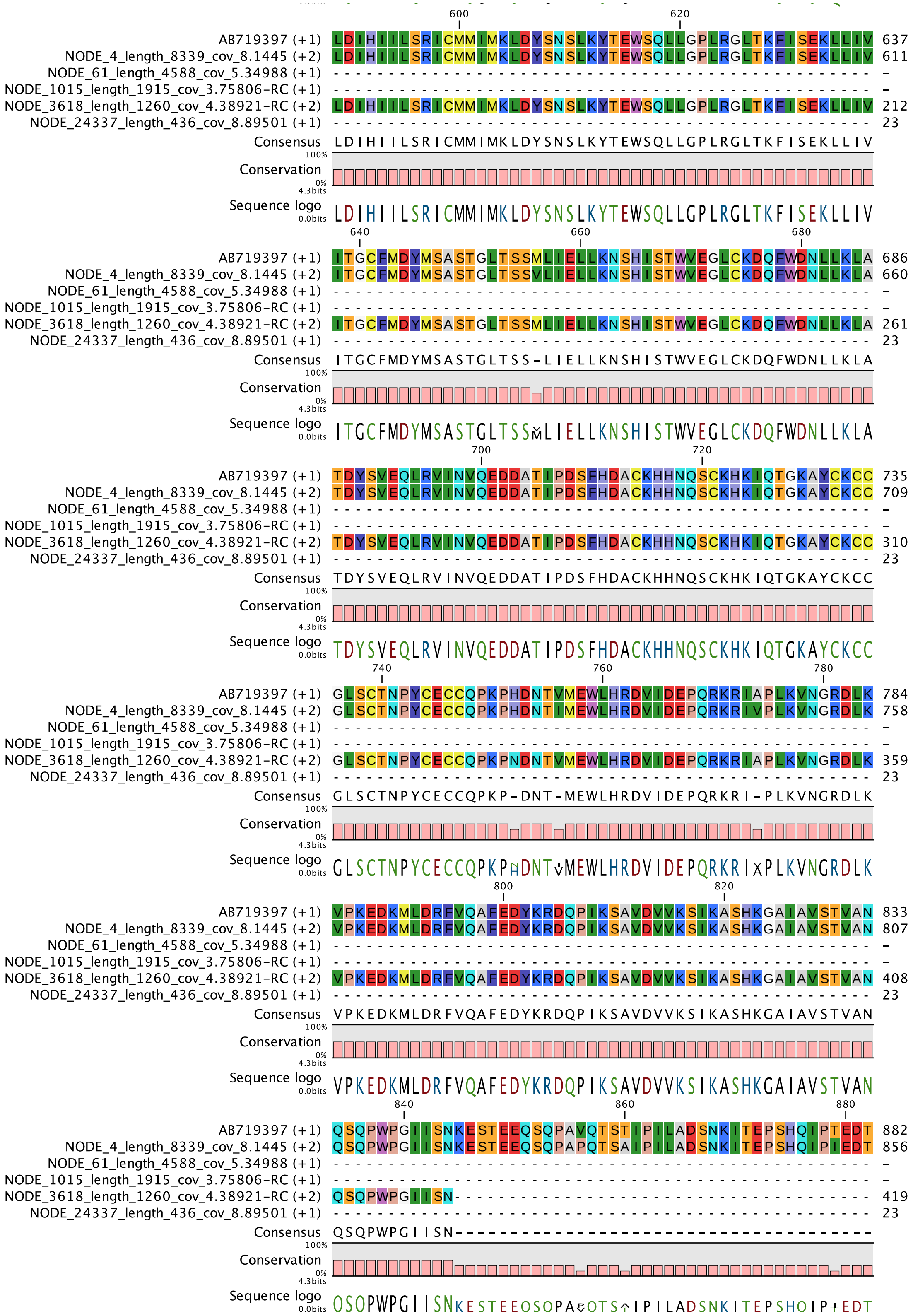
600 620
AB719397 (+1) LDIHIILSRICMMIMKLDYSNSLKYTEWSQLLGPLRGLTKFISEKLLIV 637
NODE_4_length_8339_cov_8.1445 (+2) LDIHIILSRICMMIMKLDYSNSLKYTEWSQLLGPLRGLTKFISEKLLIV 611
NODE_61_length_4588_cov_5.34988 (+1) -
NODE_1015_length_1915_cov_3.75806-RC (+1) -
NODE_3618_length_1260_cov_4.38921-RC (+2) LDIHIILSRICMMIMKLDYSNSLKYTEWSQLLGPLRGLTKFISEKLLIV 212
NODE_24337_length_436_cov_8.89501 (+1) 23
Consensus LDIHIILSRICMMIMKLDYSNSLKYTEWSQLLGPLRGLTKFISEKLLIV
Conservation
Sequence logo
640 660 680
AB719397 (+1) ITGCFMDYMSASTGLTSSMLIELLKNSHISTWVEGLCKDQFWDNLLKLA 686
NODE_4_length_8339_cov_8.1445 (+2) ITGCFMDYMSASTGLTSSVLIELLKNSHISTWVEGLCKDQFWDNLLKLA 660
NODE_61_length_4588_cov_5.34988 (+1) -
NODE_1015_length_1915_cov_3.75806-RC (+1) -
NODE_3618_length_1260_cov_4.38921-RC (+2) ITGCFMDYMSASTGLTSSMLIELLKNSHISTWVEGLCKDQFWDNLLKLA 261
NODE_24337_length_436_cov_8.89501 (+1) 23
Consensus ITGCFMDYMSASTGLTSS-LIELLKNSHISTWVEGLCKDQFWDNLLKLA
Conservation
Sequence logo
700 720
AB719397 (+1) TDYSVEQLRVINVQEDDATIPDSFHDACKHHNQSCKHKIQTGKAYCKCC 735
NODE_4_length_8339_cov_8.1445 (+2) TDYSVEQLRVINVQEDDATIPDSFHDACKHHNQSCKHKIQTGKAYCKCC 709
NODE_61_length_4588_cov_5.34988 (+1) -
NODE_1015_length_1915_cov_3.75806-RC (+1) -
NODE_3618_length_1260_cov_4.38921-RC (+2) TDYSVEQLRVINVQEDDATIPDSFHDACKHHNQSCKHKIQTGKAYCKCC 310
NODE_24337_length_436_cov_8.89501 (+1) 23
Consensus TDYSVEQLRVINVQEDDATIPDSFHDACKHHNQSCKHKIQTGKAYCKCC
Conservation
Sequence logo
740 760 780
AB719397 (+1) GLSCTNPYCECCQPKPHDNTVMEWLHRDVIDEPQRKRIAPLKVNGRDLK 784
NODE_4_length_8339_cov_8.1445 (+2) GLSCTNPYCECCQPKPHDNTIMEWLHRDVIDEPQRKRIVPLKVNGRDLK 758
NODE_61_length_4588_cov_5.34988 (+1) -
NODE_1015_length_1915_cov_3.75806-RC (+1) -
NODE_3618_length_1260_cov_4.38921-RC (+2) GLSCTNPYCECCQPKPNDNTVMEWLHRDVIDEPQRKRIAPLKVNGRDLK 359
NODE_24337_length_436_cov_8.89501 (+1) 23
Consensus GLSCTNPYCECCQPKP-DNT-MEWLHRDVIDEPQRKRI-PLKVNGRDLK
Conservation
Sequence logo
800 820
AB719397 (+1) VPKEDKMLDRFVQAFEDYKRDQPIKSAVDVVKSIKASHKGAIAVSTVAN 833
NODE_4_length_8339_cov_8.1445 (+2) VPKEDKMLDRFVQAFEDYKRDQPIKSAVDVVKSIKASHKGAIAVSTVAN 807
NODE_61_length_4588_cov_5.34988 (+1) -
NODE_1015_length_1915_cov_3.75806-RC (+1) -
NODE_3618_length_1260_cov_4.38921-RC (+2) VPKEDKMLDRFVQAFEDYKRDQPIKSAVDVVKSIKASHKGAIAVSTVAN 408
NODE_24337_length_436_cov_8.89501 (+1) 23
Consensus VPKEDKMLDRFVQAFEDYKRDQPIKSAVDVVKSIKASHKGAIAVSTVAN
Conservation
Sequence logo
840 860 880
AB719397 (+1) QSQPWPGIISNKESTEEQSQPAVQTSTIPILADSNKITEPSHQIPTEDT 882
NODE_4_length_8339_cov_8.1445 (+2) QSQPWPGIISNKESTEEQSQPAPQTSAIPILADSNKITEPSHQIPIEDT 856
NODE_61_length_4588_cov_5.34988 (+1) -
NODE_1015_length_1915_cov_3.75806-RC (+1) -
NODE_3618_length_1260_cov_4.38921-RC (+2) QSQPWPGIISN- 419
NODE_24337_length_436_cov_8.89501 (+1) 23
Consensus QSQPWPGIISN-
Conservation
Sequence logo

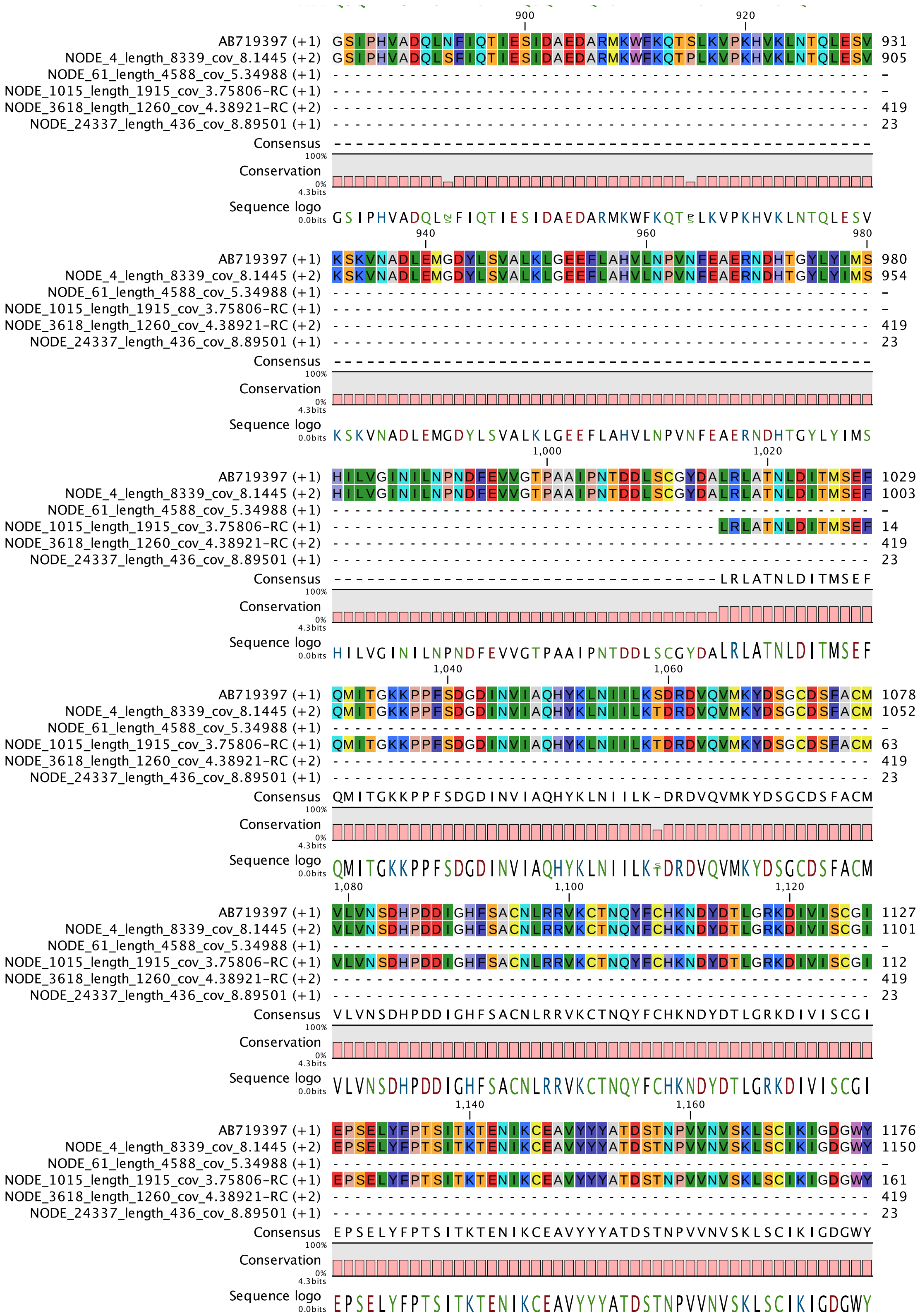
AB719397 (+1) GSIPHVADQLNFIQTIESIDAEDARMKWFKQTSLKVPKHVKLNTQLESV 931
NODE_4_length_8339_cov_8.1445 (+2) GSIPHVADQLSFIQTIESIDAEDARMKWFKQTPLKVPKHVKLNTQLESV 905
NODE_61_length_4588_cov_5.34988 (+1) –
NODE_1015_length_1915_cov_3.75806-RC (+1) –
NODE_3618_length_1260_cov_4.38921-RC (+2) 419
NODE_24337_length_436_cov_8.89501 (+1) 23
Consensus
Conservation
Sequence logo
AB719397 (+1) KSKVNADLEMGDYLSVALKLGEEFLAHVLNPVNFEAERNDHTGYLYIMS 980
NODE_4_length_8339_cov_8.1445 (+2) KSKVNADLEMGDYLSVALKLGEEFLAHVLNPVNFEAERNDHTGYLYIMS 954
AB719397 (+1) HILVGINILNPNDFEVVGTPAAIPNTDDLSCGYDALRLATNLDITMSEF 1029
NODE_4_length_8339_cov_8.1445 (+2) HILVGINILNPNDFEVVGTPAAIPNTDDLSCGYDALRLATNLDITMSEF 1003
NODE_1015_length_1915_cov_3.75806-RC (+1) LRLATNLDITMSEF 14
Consensus LRLATNLDITMSEF
AB719397 (+1) QMITGKKPPFSDGDINVIAQHYKLNIILKSDRDVQVMKYDSGCDSFACM 1078
NODE_4_length_8339_cov_8.1445 (+2) QMITGKKPPFSDGDINVIAQHYKLNIILKTDRDVQVMKYDSGCDSFACM 1052
NODE_1015_length_1915_cov_3.75806-RC (+1) QMITGKKPPFSDGDINVIAQHYKLNIILKTDRDVQVMKYDSGCDSFACM 63
Consensus QMITGKKPPFSDGDINVIAQHYKLNIILK-DRDVQVMKYDSGCDSFACM
AB719397 (+1) VLVNSDHPDDIGHFSACNLRRVKCTNQYFCHKNDYDTLGRKDIVISCGI 1127
NODE_4_length_8339_cov_8.1445 (+2) VLVNSDHPDDIGHFSACNLRRVKCTNQYFCHKNDYDTLGRKDIVISCGI 1101
NODE_1015_length_1915_cov_3.75806-RC (+1) VLVNSDHPDDIGHFSACNLRRVKCTNQYFCHKNDYDTLGRKDIVISCGI 112
Consensus VLVNSDHPDDIGHFSACNLRRVKCTNQYFCHKNDYDTLGRKDIVISCGI
AB719397 (+1) EPSELYFPTSITKTENIKCEAVYYYATDSTNPVVNVSKLSCIKIGDGWY 1176
NODE_4_length_8339_cov_8.1445 (+2) EPSELYFPTSITKTENIKCEAVYYYATDSTNPVVNVSKLSCIKIGDGWY 1150
NODE_1015_length_1915_cov_3.75806-RC (+1) EPSELYFPTSITKTENIKCEAVYYYATDSTNPVVNVSKLSCIKIGDGWY 161
Consensus EPSELYFPTSITKTENIKCEAVYYYATDSTNPVVNVSKLSCIKIGDGWY

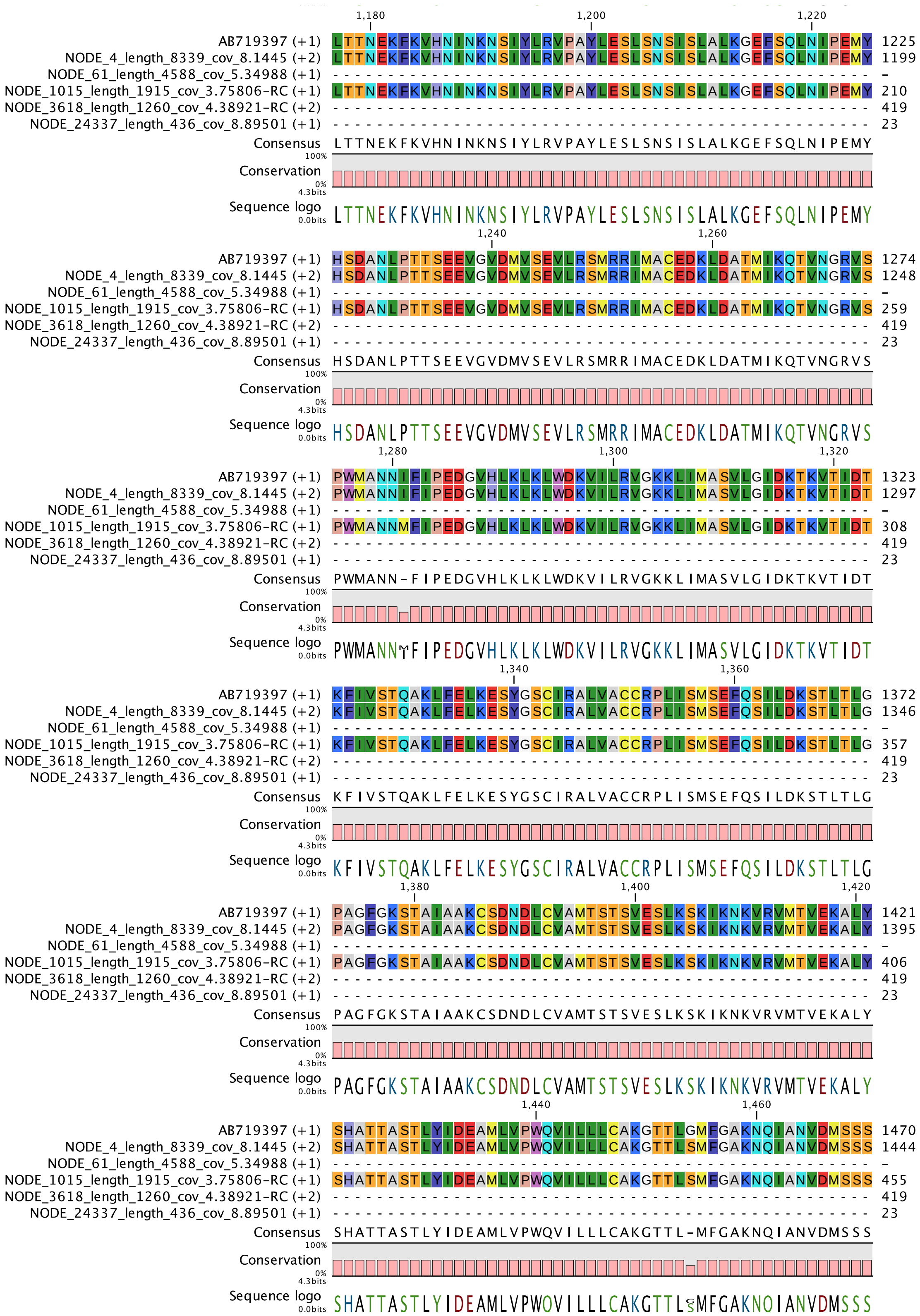

1,180 1,200 1,220
AB719397 (+1) LTTNEKFKVHNINKNSIYLRVPAYLESLSNSISLALKGEFSQLNIPEMY 1225
NODE_4_length_8339_cov_8.1445 (+2) LTTNEKFKVHNINKNSIYLRVPAYLESLSNSISLALKGEFSQLNIPEMY 1199
NODE_61_length_4588_cov_5.34988 (+1) –
NODE_1015_length_1915_cov_3.75806-RC (+1) LTTNEKFKVHNINKNSIYLRVPAYLESLSNSISLALKGEFSQLNIPEMY 210
NODE_3618_length_1260_cov_4.38921-RC (+2) 419
NODE_24337_length_436_cov_8.89501 (+1) 23
Consensus LTTNEKFKVHNINKNSIYLRVPAYLESLSNSISLALKGEFSQLNIPEMY
Conservation
Sequence logo
1,240 1,260
AB719397 (+1) HSDANLPTTSEEVGVDMVSEVLRSMRRIMACEDKLDATMIKQTVNGRVS 1274
NODE_4_length_8339_cov_8.1445 (+2) HSDANLPTTSEEVGVDMVSEVLRSMRRIMACEDKLDATMIKQTVNGRVS 1248
NODE_61_length_4588_cov_5.34988 (+1) –
NODE_1015_length_1915_cov_3.75806-RC (+1) HSDANLPTTSEEVGVDMVSEVLRSMRRIMACEDKLDATMIKQTVNGRVS 259
NODE_3618_length_1260_cov_4.38921-RC (+2) 419
NODE_24337_length_436_cov_8.89501 (+1) 23
Consensus HSDANLPTTSEEVGVDMVSEVLRSMRRIMACEDKLDATMIKQTVNGRVS
Conservation
Sequence logo
1,280 1,300 1,320
AB719397 (+1) PWMANNIFIPEDGVHLKLKLWDKVILRVGKKLIMASVLGIDKTKVTIDT 1323
NODE_4_length_8339_cov_8.1445 (+2) PWMANNIFIPEDGVHLKLKLWDKVILRVGKKLIMASVLGIDKTKVTIDT 1297
NODE_61_length_4588_cov_5.34988 (+1) –
NODE_1015_length_1915_cov_3.75806-RC (+1) PWMANNMFIPEDGVHLKLKLWDKVILRVGKKLIMASVLGIDKTKVTIDT 308
NODE_3618_length_1260_cov_4.38921-RC (+2) 419
NODE_24337_length_436_cov_8.89501 (+1) 23
Consensus PWMANN-FIPEDGVHLKLKLWDKVILRVGKKLIMASVLGIDKTKVTIDT
Conservation
Sequence logo
1,340 1,360
AB719397 (+1) KFIVSTQAKLFELKESYGSCIRALVACCRPLISMSEFQSILDKSTLTLG 1372
NODE_4_length_8339_cov_8.1445 (+2) KFIVSTQAKLFELKESYGSCIRALVACCRPLISMSEFQSILDKSTLTLG 1346
NODE_61_length_4588_cov_5.34988 (+1) –
NODE_1015_length_1915_cov_3.75806-RC (+1) KFIVSTQAKLFELKESYGSCIRALVACCRPLISMSEFQSILDKSTLTLG 357
NODE_3618_length_1260_cov_4.38921-RC (+2) 419
NODE_24337_length_436_cov_8.89501 (+1) 23
Consensus KFIVSTQAKLFELKESYGSCIRALVACCRPLISMSEFQSILDKSTLTLG
Conservation
Sequence logo
1,380 1,400 1,420
AB719397 (+1) PAGFGKSTAIAAKCSDNDLCVAMTSTSVESLKSKIKNKVRVMTVEKALY 1421
NODE_4_length_8339_cov_8.1445 (+2) PAGFGKSTAIAAKCSDNDLCVAMTSTSVESLKSKIKNKVRVMTVEKALY 1395
NODE_61_length_4588_cov_5.34988 (+1) –
NODE_1015_length_1915_cov_3.75806-RC (+1) PAGFGKSTAIAAKCSDNDLCVAMTSTSVESLKSKIKNKVRVMTVEKALY 406
NODE_3618_length_1260_cov_4.38921-RC (+2) 419
NODE_24337_length_436_cov_8.89501 (+1) 23
Consensus PAGFGKSTAIAAKCSDNDLCVAMTSTSVESLKSKIKNKVRVMTVEKALY
Conservation
Sequence logo
1,440 1,460
AB719397 (+1) SHATTASTLYIDEAMLVPWQVILLLCAKGTTLGMFGAKNQIANVDMSSS 1470
NODE_4_length_8339_cov_8.1445 (+2) SHATTASTLYIDEAMLVPWQVILLLCAKGTTLSMFGAKNQIANVDMSSS 1444
NODE_61_length_4588_cov_5.34988 (+1) –
NODE_1015_length_1915_cov_3.75806-RC (+1) SHATTASTLYIDEAMLVPWQVILLLCAKGTTLSMFGAKNQIANVDMSSS 455
NODE_3618_length_1260_cov_4.38921-RC (+2) 419
NODE_24337_length_436_cov_8.89501 (+1) 23
Consensus SHATTASTLYIDEAMLVPWQVILLLCAKGTTL-MFGAKNQIANVDMSSS
Conservation
Sequence logo

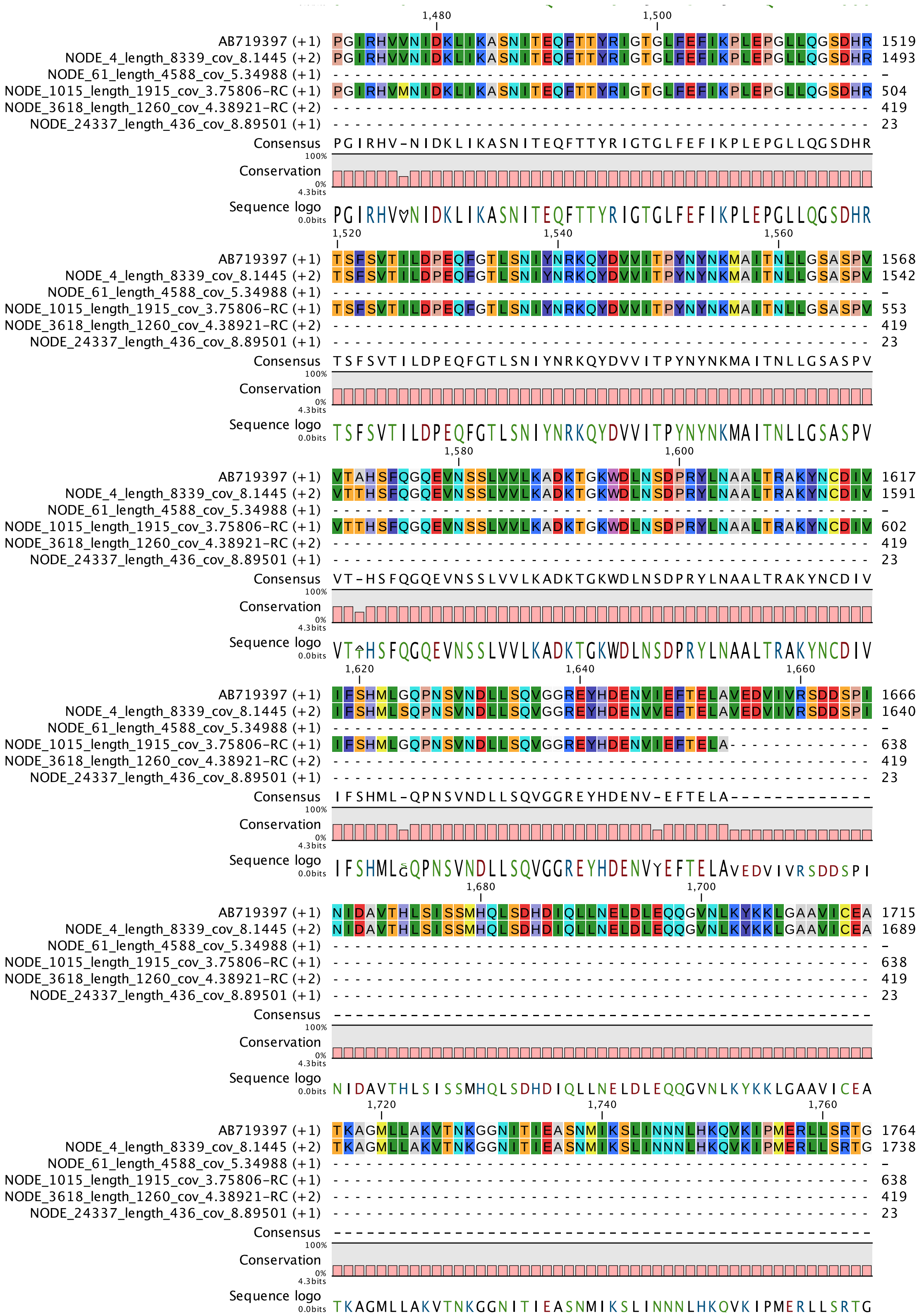
1,480 1,500
AB719397 (+1) PGIRHVVNIDKLIKASNITEQFTTYRIGTGLFEFIKPLEPGLLQGSDHR 1519
NODE_4_length_8339_cov_8.1445 (+2) PGIRHVVNIDKLIKASNITEQFTTYRIGTGLFEFIKPLEPGLLQGSDHR 1493
NODE_61_length_4588_cov_5.34988 (+1) –
NODE_1015_length_1915_cov_3.75806-RC (+1) PGIRHVMNIDKLIKASNITEQFTTYRIGTGLFEFIKPLEPGLLQGSDHR 504
NODE_3618_length_1260_cov_4.38921-RC (+2) 419
NODE_24337_length_436_cov_8.89501 (+1) 23
Consensus PGIRHV-NIDKLIKASNITEQFTTYRIGTGLFEFIKPLEPGLLQGSDHR
Conservation
Sequence logo
1,520 1,540 1,560
AB719397 (+1) TSFSVTILDPEQFGTLSNIYNRKQYDVVITPYNYNKMAITNLLGSASPV 1568
NODE_4_length_8339_cov_8.1445 (+2) TSFSVTILDPEQFGTLSNIYNRKQYDVVITPYNYNKMAITNLLGSASPV 1542
NODE_61_length_4588_cov_5.34988 (+1) –
NODE_1015_length_1915_cov_3.75806-RC (+1) TSFSVTILDPEQFGTLSNIYNRKQYDVVITPYNYNKMAITNLLGSASPV 553
NODE_3618_length_1260_cov_4.38921-RC (+2) 419
NODE_24337_length_436_cov_8.89501 (+1) 23
Consensus TSFSVTILDPEQFGTLSNIYNRKQYDVVITPYNYNKMAITNLLGSASPV
Conservation
Sequence logo
1,580 1,600
AB719397 (+1) VTAHSFQGQEVNSSLVVLKADKTGKWDLNSDPRYLNAALTRAKYNCDIV 1617
NODE_4_length_8339_cov_8.1445 (+2) VTTHSFQGQEVNSSLVVLKADKTGKWDLNSDPRYLNAALTRAKYNCDIV 1591
NODE_61_length_4588_cov_5.34988 (+1) –
NODE_1015_length_1915_cov_3.75806-RC (+1) VTTHSFQGQEVNSSLVVLKADKTGKWDLNSDPRYLNAALTRAKYNCDIV 602
NODE_3618_length_1260_cov_4.38921-RC (+2) 419
NODE_24337_length_436_cov_8.89501 (+1) 23
Consensus VT-HSFQGQEVNSSLVVLKADKTGKWDLNSDPRYLNAALTRAKYNCDIV
Conservation
Sequence logo
1,620 1,640 1,660
AB719397 (+1) IFSHMLGQPNSVNDLLSQVGGREYHDENVIEFTELAVEDVIVRSDDSPI 1666
NODE_4_length_8339_cov_8.1445 (+2) IFSHMLSQPNSVNDLLSQVGGREYHDENVVEFTELAVEDVIVRSDDSPI 1640
NODE_61_length_4588_cov_5.34988 (+1) –
NODE_1015_length_1915_cov_3.75806-RC (+1) IFSHMLGQPNSVNDLLSQVGGREYHDENVIEFTELA------------- 638
NODE_3618_length_1260_cov_4.38921-RC (+2) 419
NODE_24337_length_436_cov_8.89501 (+1) 23
Consensus IFSHML-QPNSVNDLLSQVGGREYHDENV-EFTELA-------------
Conservation
Sequence logo
1,680 1,700
AB719397 (+1) NIDAVTHLSISSMHQLSDHDIQLLNELDLEQQGVNLKYKKLGAAVICEA 1715
NODE_4_length_8339_cov_8.1445 (+2) NIDAVTHLSISSMHQLSDHDIQLLNELDLEQQGVNLKYKKLGAAVICEA 1689
NODE_61_length_4588_cov_5.34988 (+1) –
NODE_1015_length_1915_cov_3.75806-RC (+1) 638
NODE_3618_length_1260_cov_4.38921-RC (+2) 419
NODE_24337_length_436_cov_8.89501 (+1) 23
Consensus
Conservation
Sequence logo
1,720 1,740 1,760
AB719397 (+1) TKAGMLLAKVTNKGGNITIEASNMIKSLINNNLHKQVKIPMERLLSRTG 1764
NODE_4_length_8339_cov_8.1445 (+2) TKAGMLLAKVTNKGGNITIEASNMIKSLINNNLHKQVKIPMERLLSRTG 1738
NODE_61_length_4588_cov_5.34988 (+1) –
NODE_1015_length_1915_cov_3.75806-RC (+1) 638
NODE_3618_length_1260_cov_4.38921-RC (+2) 419
NODE_24337_length_436_cov_8.89501 (+1) 23
Consensus
Conservation
Sequence logo

```
                                           1,780                         1,800
AB719397 (+1)                              MAKTHKVTLSTQSACKLRELLWIVDKTKDGILTLPLNDGLITITKTSGC  1813
NODE_4_length_8339_cov_8.1445 (+2)         MAKTHKVTLSTQSACKLRELLWIVDKTKDGILTLPLNDGLITITKTSGC  1787
NODE_61_length_4588_cov_5.34988 (+1)       -------------------------------------------------  -
NODE_1015_length_1915_cov_3.75806-RC (+1)  -------------------------------------------------  638
NODE_3618_length_1260_cov_4.38921-RC (+2)  -------------------------------------------------  419
NODE_24337_length_436_cov_8.89501 (+1)     -------------------------------------------------  23
Consensus                                  -------------------------------------------------
Conservation (100% / 0%)
Sequence logo (4.3bits / 0.0bits)          MAKTHKVTLSTQSACKLRELLWIVDKTKDGILTLPLNDGLITITKTSGC

                                           1,820                         1,840                         1,860
AB719397 (+1)                              PLFAGCKMQIDDEWIYITDAWQAITSRTITYNFSASPCKLNDILIWMQL  1862
NODE_4_length_8339_cov_8.1445 (+2)         PLFAGCKMQIDDEWIYITDAWQAITSRTITYNFSASPCKLNDILIWMQL  1836
NODE_61_length_4588_cov_5.34988 (+1)       -------------------------------------------------  -
NODE_1015_length_1915_cov_3.75806-RC (+1)  -------------------------------------------------  638
NODE_3618_length_1260_cov_4.38921-RC (+2)  -------------------------------------------------  419
NODE_24337_length_436_cov_8.89501 (+1)     -------------------------------------------------  23
Consensus                                  -------------------------------------------------
Conservation (100% / 0%)
Sequence logo (4.3bits / 0.0bits)          PLFAGCKMQIDDEWIYITDAWQAITSRTITYNFSASPCKLNDILIWMQL

                                           1,880                         1,900
AB719397 (+1)                              ELPNIHLIPHNGTTWLDVIKGNLVTTYWQATERLTQVKYWMLNALAFSE  1911
NODE_4_length_8339_cov_8.1445 (+2)         ELPNIHLIPHNGTTWLDVIKGNLVTTYWQATERLTQVKYWMLNALAFSE  1885
NODE_61_length_4588_cov_5.34988 (+1)       -------------------------------------------------  -
NODE_1015_length_1915_cov_3.75806-RC (+1)  -------------------------------------------------  638
NODE_3618_length_1260_cov_4.38921-RC (+2)  -------------------------------------------------  419
NODE_24337_length_436_cov_8.89501 (+1)     -------------------------------------------------  23
Consensus                                  -------------------------------------------------
Conservation (100% / 0%)
Sequence logo (4.3bits / 0.0bits)          ELPNIHLIPHNGTTWLDVIKGNLVTTYWQATERLTQVKYWMLNALAFSE

                                           1,920                         1,940                         1,960
AB719397 (+1)                              NPKCYGNNLRNVSKTNKFMRKLKIKGAIDYHQSVINHKTLILLEQGWFK  1960
NODE_4_length_8339_cov_8.1445 (+2)         NPKCYGNNLRNVSKTNKFMRKLKIKGAIDYHQSVINHKTLILLEQGWFK  1934
NODE_61_length_4588_cov_5.34988 (+1)       -------------------------------------------------  -
NODE_1015_length_1915_cov_3.75806-RC (+1)  -------------------------------------------------  638
NODE_3618_length_1260_cov_4.38921-RC (+2)  -------------------------------------------------  419
NODE_24337_length_436_cov_8.89501 (+1)     -------------------------------------------------  23
Consensus                                  -------------------------------------------------
Conservation (100% / 0%)
Sequence logo (4.3bits / 0.0bits)          NPKCYGNNLRNVSKTNKFMRKLKIKGAIDYHQSVINHKTLILLEQGWFK

                                           1,980                         2,000
AB719397 (+1)                              SYVTLCSEDGSLISIHKTSKDLEIKEFIVEALQDIQQSEEADKIKGNDI  2009
NODE_4_length_8339_cov_8.1445 (+2)         SYVTLCSEDGSLISIHKTSKDLEIKEFIVEALQDIQQSEEADKIKGNDI  1983
NODE_61_length_4588_cov_5.34988 (+1)       -------------------------------------------------  -
NODE_1015_length_1915_cov_3.75806-RC (+1)  -------------------------------------------------  638
NODE_3618_length_1260_cov_4.38921-RC (+2)  -------------------------------------------------  419
NODE_24337_length_436_cov_8.89501 (+1)     -------------------------------------------------  23
Consensus                                  -------------------------------------------------
Conservation (100% / 0%)
Sequence logo (4.3bits / 0.0bits)          SYVTLCSEDGSLISIHKTSKDLEIKEFIVEALQDIQQSEEADKIKGNDI

                                           2,020                         2,040
AB719397 (+1)                              GEMDIPGLLMPMSLHKQKRTPVYQYAVNELGKVISHLGKVSHDLIRVNR  2058
NODE_4_length_8339_cov_8.1445 (+2)         GEMDIPGLLMPMSLHKQKRTPVYQYAVNELGKVISHLGKVSHDLIRVNR  2032
NODE_61_length_4588_cov_5.34988 (+1)       -------------------------------------------------  -
NODE_1015_length_1915_cov_3.75806-RC (+1)  -------------------------------------------------  638
NODE_3618_length_1260_cov_4.38921-RC (+2)  -------------------------------------------------  419
NODE_24337_length_436_cov_8.89501 (+1)     -------------------------------------------------  23
Consensus                                  -------------------------------------------------
Conservation (100% / 0%)
Sequence logo (4.3bits / 0.0bits)          GEMDIPGLLMPMSLHKQKRTPVYQYAVNELGKVISHLGKVSHDLIRVNR
```

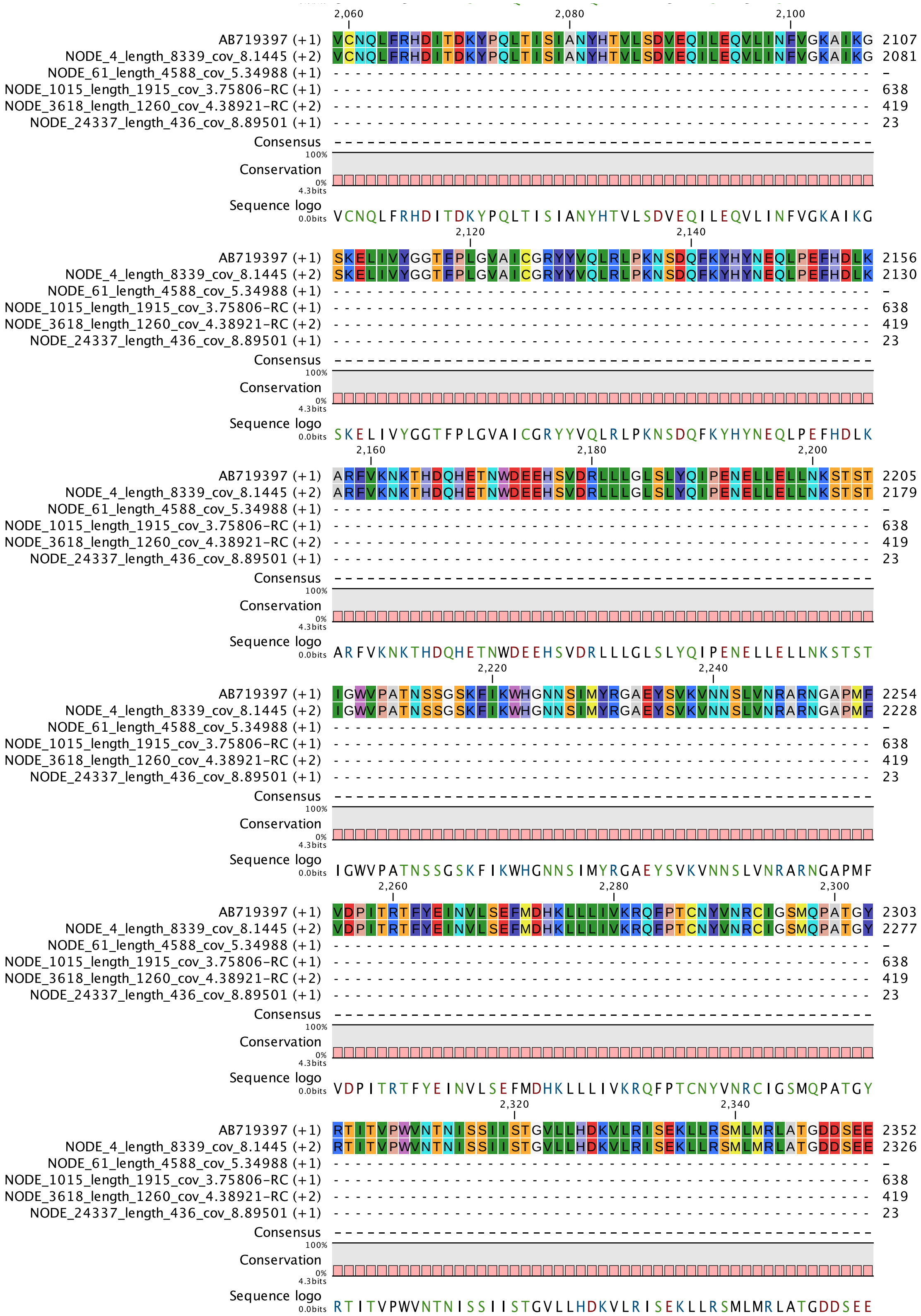
2,060 2,080 2,100
AB719397 (+1) VCNQLFRHDITDKYPQLTISIANYHTVLSDVEQILEQVLINFVGKAIKG 2107
NODE_4_length_8339_cov_8.1445 (+2) VCNQLFRHDITDKYPQLTISIANYHTVLSDVEQILEQVLINFVGKAIKG 2081
NODE_61_length_4588_cov_5.34988 (+1) -
NODE_1015_length_1915_cov_3.75806-RC (+1) 638
NODE_3618_length_1260_cov_4.38921-RC (+2) 419
NODE_24337_length_436_cov_8.89501 (+1) 23
Consensus
Conservation
100%
0%
Sequence logo
4.3bits
0.0bits
VCNQLFRHDITDKYPQLTISIANYHTVLSDVEQILEQVLINFVGKAIKG
2,120 2,140
AB719397 (+1) SKELIVYGGTFPLGVAICGRYYVQLRLPKNSDQFKYHYNEQLPEFHDLK 2156
NODE_4_length_8339_cov_8.1445 (+2) SKELIVYGGTFPLGVAICGRYYVQLRLPKNSDQFKYHYNEQLPEFHDLK 2130
NODE_61_length_4588_cov_5.34988 (+1) -
NODE_1015_length_1915_cov_3.75806-RC (+1) 638
NODE_3618_length_1260_cov_4.38921-RC (+2) 419
NODE_24337_length_436_cov_8.89501 (+1) 23
Consensus
Conservation
Sequence logo
SKELIVYGGTFPLGVAICGRYYVQLRLPKNSDQFKYHYNEQLPEFHDLK
2,160 2,180 2,200
AB719397 (+1) ARFVKNKTHDQHETNWDEEHSVDRLLLGLSLYQIPENELLELLNKSTST 2205
NODE_4_length_8339_cov_8.1445 (+2) ARFVKNKTHDQHETNWDEEHSVDRLLLGLSLYQIPENELLELLNKSTST 2179
NODE_61_length_4588_cov_5.34988 (+1) -
NODE_1015_length_1915_cov_3.75806-RC (+1) 638
NODE_3618_length_1260_cov_4.38921-RC (+2) 419
NODE_24337_length_436_cov_8.89501 (+1) 23
Consensus
Conservation
Sequence logo
ARFVKNKTHDQHETNWDEEHSVDRLLLGLSLYQIPENELLELLNKSTST
2,220 2,240
AB719397 (+1) IGWVPATNSSGSKFIKWHGNNSIMYRGAEYSVKVNNSLVNRARNGAPMF 2254
NODE_4_length_8339_cov_8.1445 (+2) IGWVPATNSSGSKFIKWHGNNSIMYRGAEYSVKVNNSLVNRARNGAPMF 2228
NODE_61_length_4588_cov_5.34988 (+1) -
NODE_1015_length_1915_cov_3.75806-RC (+1) 638
NODE_3618_length_1260_cov_4.38921-RC (+2) 419
NODE_24337_length_436_cov_8.89501 (+1) 23
Consensus
Conservation
Sequence logo
IGWVPATNSSGSKFIKWHGNNSIMYRGAEYSVKVNNSLVNRARNGAPMF
2,260 2,280 2,300
AB719397 (+1) VDPITRTFYEINVLSEFMDHKLLLIVKRQFPTCNYVNRCIGSMQPATGY 2303
NODE_4_length_8339_cov_8.1445 (+2) VDPITRTFYEINVLSEFMDHKLLLIVKRQFPTCNYVNRCIGSMQPATGY 2277
NODE_61_length_4588_cov_5.34988 (+1) -
NODE_1015_length_1915_cov_3.75806-RC (+1) 638
NODE_3618_length_1260_cov_4.38921-RC (+2) 419
NODE_24337_length_436_cov_8.89501 (+1) 23
Consensus
Conservation
Sequence logo
VDPITRTFYEINVLSEFMDHKLLLIVKRQFPTCNYVNRCIGSMQPATGY
2,320 2,340
AB719397 (+1) RTITVPWVNTNISSIISTGVLLHDKVLRISEKLLRSMLMRLATGDDSEE 2352
NODE_4_length_8339_cov_8.1445 (+2) RTITVPWVNTNISSIISTGVLLHDKVLRISEKLLRSMLMRLATGDDSEE 2326
NODE_61_length_4588_cov_5.34988 (+1) -
NODE_1015_length_1915_cov_3.75806-RC (+1) 638
NODE_3618_length_1260_cov_4.38921-RC (+2) 419
NODE_24337_length_436_cov_8.89501 (+1) 23
Consensus
Conservation
Sequence logo
RTITVPWVNTNISSIISTGVLLHDKVLRISEKLLRSMLMRLATGDDSEE

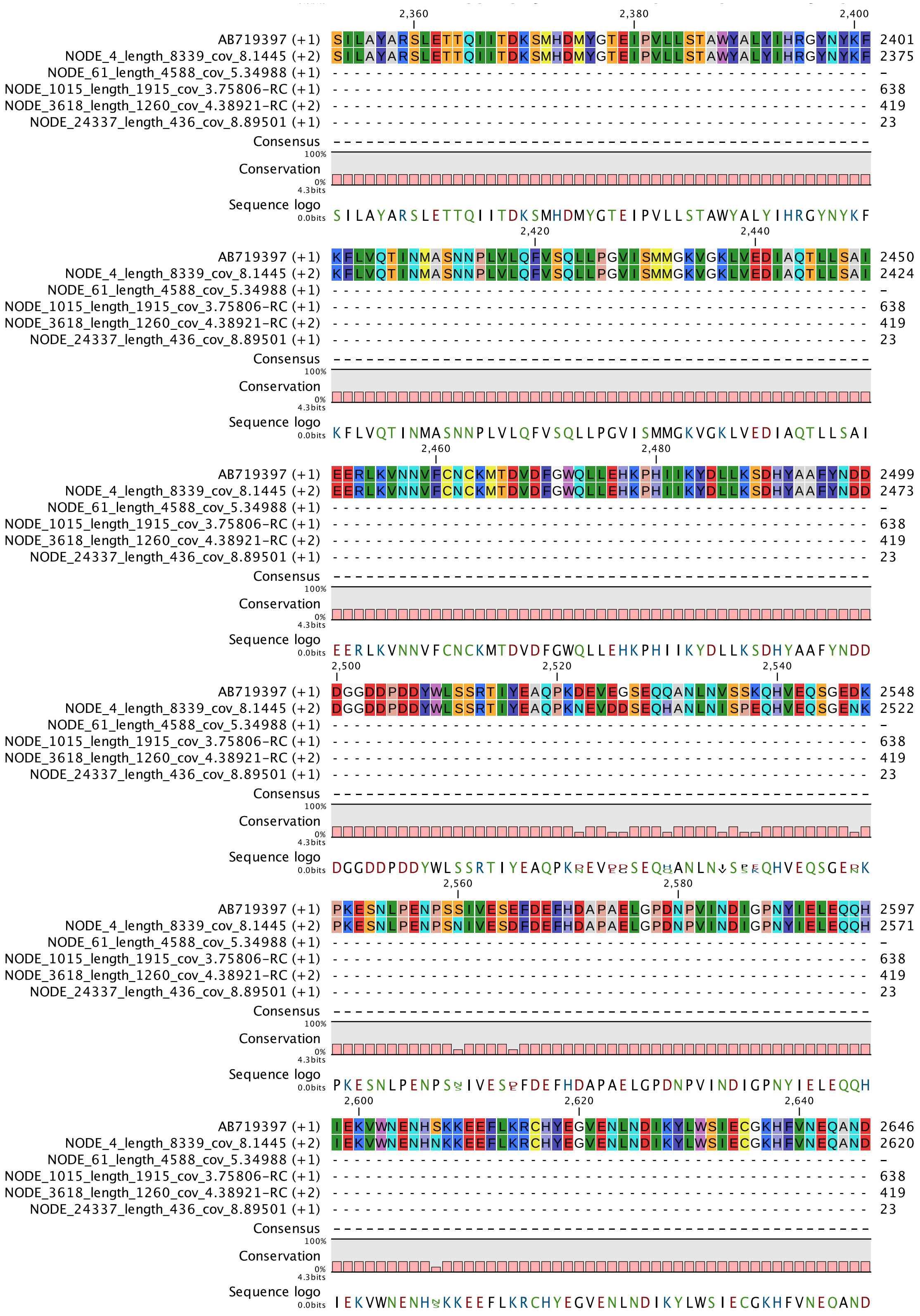
2,360 2,380 2,400
AB719397 (+1) SILAYARSLETTQIITDKSMHDMYGTEIPVLLSTAWYALYIHRGYNYKF 2401
NODE_4_length_8339_cov_8.1445 (+2) SILAYARSLETTQIITDKSMHDMYGTEIPVLLSTAWYALYIHRGYNYKF 2375
NODE_61_length_4588_cov_5.34988 (+1) –
NODE_1015_length_1915_cov_3.75806-RC (+1) 638
NODE_3618_length_1260_cov_4.38921-RC (+2) 419
NODE_24337_length_436_cov_8.89501 (+1) 23
Consensus
Conservation
Sequence logo
SILAYARSLETTQIITDKSMHDMYGTEIPVLLSTAWYALYIHRGYNYKF
2,420 2,440
AB719397 (+1) KFLVQTINMASNNPLVLQFVSQLLPGVISMMGKVGKLVEDIAQTLLSAI 2450
NODE_4_length_8339_cov_8.1445 (+2) KFLVQTINMASNNPLVLQFVSQLLPGVISMMGKVGKLVEDIAQTLLSAI 2424
NODE_61_length_4588_cov_5.34988 (+1) –
NODE_1015_length_1915_cov_3.75806-RC (+1) 638
NODE_3618_length_1260_cov_4.38921-RC (+2) 419
NODE_24337_length_436_cov_8.89501 (+1) 23
Consensus
Conservation
Sequence logo
KFLVQTINMASNNPLVLQFVSQLLPGVISMMGKVGKLVEDIAQTLLSAI
2,460 2,480
AB719397 (+1) EERLKVNNVFCNCKMTDVDFGWQLLEHKPHIIKYDLLKSDHYAAFYNDD 2499
NODE_4_length_8339_cov_8.1445 (+2) EERLKVNNVFCNCKMTDVDFGWQLLEHKPHIIKYDLLKSDHYAAFYNDD 2473
NODE_61_length_4588_cov_5.34988 (+1) –
NODE_1015_length_1915_cov_3.75806-RC (+1) 638
NODE_3618_length_1260_cov_4.38921-RC (+2) 419
NODE_24337_length_436_cov_8.89501 (+1) 23
Consensus
Conservation
Sequence logo
EERLKVNNVFCNCKMTDVDFGWQLLEHKPHIIKYDLLKSDHYAAFYNDD
2,500 2,520 2,540
AB719397 (+1) DGGDDPDDYWLSSRTIYEAQPKDEVEGSEQQANLNVSSKQHVEQSGEDK 2548
NODE_4_length_8339_cov_8.1445 (+2) DGGDDPDDYWLSSRTIYEAQPKNEVDDSEQHANLNISPEQHVEQSGENK 2522
NODE_61_length_4588_cov_5.34988 (+1) –
NODE_1015_length_1915_cov_3.75806-RC (+1) 638
NODE_3618_length_1260_cov_4.38921-RC (+2) 419
NODE_24337_length_436_cov_8.89501 (+1) 23
Consensus
Conservation
Sequence logo
2,560 2,580
AB719397 (+1) PKESNLPENPSSIVESEFDEFHDAPAELGPDNPVINDIGPNYIELEQQH 2597
NODE_4_length_8339_cov_8.1445 (+2) PKESNLPENPSNIVESDFDEFHDAPAELGPDNPVINDIGPNYIELEQQH 2571
NODE_61_length_4588_cov_5.34988 (+1) –
NODE_1015_length_1915_cov_3.75806-RC (+1) 638
NODE_3618_length_1260_cov_4.38921-RC (+2) 419
NODE_24337_length_436_cov_8.89501 (+1) 23
Consensus
Conservation
Sequence logo
2,600 2,620 2,640
AB719397 (+1) IEKVWNENHSKKEEFLKRCHYEGVENLNDIKYLWSIECGKHFVNEQAND 2646
NODE_4_length_8339_cov_8.1445 (+2) IEKVWNENHNKKEEFLKRCHYEGVENLNDIKYLWSIECGKHFVNEQAND 2620
NODE_61_length_4588_cov_5.34988 (+1) –
NODE_1015_length_1915_cov_3.75806-RC (+1) 638
NODE_3618_length_1260_cov_4.38921-RC (+2) 419
NODE_24337_length_436_cov_8.89501 (+1) 23
Consensus
Conservation
Sequence logo

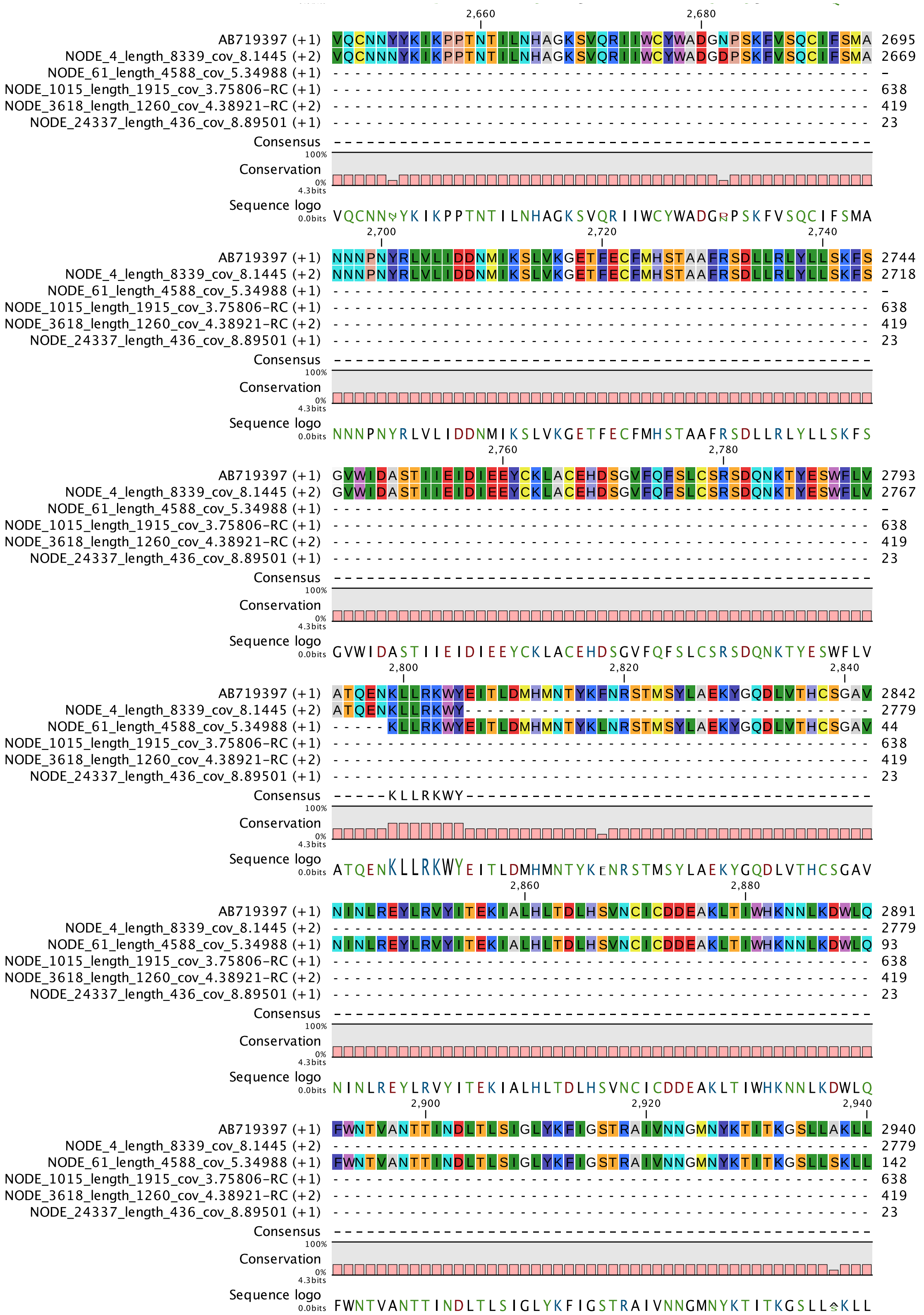
AB719397 (+1) VQCNNYYKIKPPTNTILNHAGKSVQRIIWCYWADGNPSKFVSQCIFSMA 2695
NODE_4_length_8339_cov_8.1445 (+2) VQCNNNYKIKPPTNTILNHAGKSVQRIIWCYWADGDPSKFVSQCIFSMA 2669
NODE_61_length_4588_cov_5.34988 (+1) –
NODE_1015_length_1915_cov_3.75806-RC (+1) 638
NODE_3618_length_1260_cov_4.38921-RC (+2) 419
NODE_24337_length_436_cov_8.89501 (+1) 23
AB719397 (+1) NNNPNYRLVLIDDNMIKSLVKGETFECFMHSTAAFRSDLLRLYLLSKFS 2744
NODE_4_length_8339_cov_8.1445 (+2) NNNPNYRLVLIDDNMIKSLVKGETFECFMHSTAAFRSDLLRLYLLSKFS 2718
AB719397 (+1) GVWIDASTIIEIDIEEYCKLACEHDSGVFQFSLCSRSDQNKTYESWFLV 2793
NODE_4_length_8339_cov_8.1445 (+2) GVWIDASTIIEIDIEEYCKLACEHDSGVFQFSLCSRSDQNKTYESWFLV 2767
AB719397 (+1) ATQENKLLRKWYEITLDMHMNTYKFNRSTMSYLAEKYGQDLVTHCSGAV 2842
NODE_4_length_8339_cov_8.1445 (+2) ATQENKLLRKWY 2779
NODE_61_length_4588_cov_5.34988 (+1) KLLRKWYEITLDMHMNTYKLNRSTMSYLAEKYGQDLVTHCSGAV 44
AB719397 (+1) NINLREYLRVYITEKIALHLTDLHSVNCICDDEAKLTIWHKNNLKDWLQ 2891
NODE_61_length_4588_cov_5.34988 (+1) NINLREYLRVYITEKIALHLTDLHSVNCICDDEAKLTIWHKNNLKDWLQ 93
AB719397 (+1) FWNTVANTTINDLTLSIGLYKFIGSTRAIVNNGMNYKTITKGSLLAKLL 2940
NODE_61_length_4588_cov_5.34988 (+1) FWNTVANTTINDLTLSIGLYKFIGSTRAIVNNGMNYKTITKGSLLSKLL 142
Consensus
Conservation
Sequence logo

```
                                                        2,960                     2,980
AB719397 (+1)                              TYIPVKYNIKTMERIIFNAPSLPINLPPKVSLYQKTNCVLLCLGSTGDM 2989
NODE_4_length_8339_cov_8.1445 (+2)         ------------------------------------------------- 2779
NODE_61_length_4588_cov_5.34988 (+1)       TYMPVKYNIKTMERIIFNAPSLPINLPPKVSLYQKTNCVLLCLGSTGDM 191
NODE_1015_length_1915_cov_3.75806-RC (+1)  ------------------------------------------------- 638
NODE_3618_length_1260_cov_4.38921-RC (+2)  ------------------------------------------------- 419
NODE_24337_length_436_cov_8.89501 (+1)     ------------------------------------------------- 23
Consensus                                  -------------------------------------------------
Conservation (100% / 0%)
Sequence logo (4.3bits / 0.0bits)          TY[illegible]PVKYNIKTMERIIFNAPSLPINLPPKVSLYQKTNCVLLCLGSTGDM

                                                3,000                     3,020
AB719397 (+1)                              IPYLVCYDYLAGHGNYPLLISHKDHEVLVKGRRFHGLSIKSEDVMGIAM 3038
NODE_4_length_8339_cov_8.1445 (+2)         ------------------------------------------------- 2779
NODE_61_length_4588_cov_5.34988 (+1)       IPYLVCYDYLAGHGNYPLLISHKDHEVLVKGRRFHGLSIKSEDVMGIAM 240
NODE_1015_length_1915_cov_3.75806-RC (+1)  ------------------------------------------------- 638
NODE_3618_length_1260_cov_4.38921-RC (+2)  ------------------------------------------------- 419
NODE_24337_length_436_cov_8.89501 (+1)     ------------------------------------------------- 23
Consensus                                  -------------------------------------------------
Conservation (100% / 0%)
Sequence logo (4.3bits / 0.0bits)          IPYLVCYDYLAGHGNYPLLISHKDHEVLVKGRRFHGLSIKSEDVMGIAM

                                           3,040                3,060                3,080
AB719397 (+1)                              NLLYEKEITSSILGYHLMRNIAKETIEILTLYKSHNIKLYIYNPLYPAI 3087
NODE_4_length_8339_cov_8.1445 (+2)         ------------------------------------------------- 2779
NODE_61_length_4588_cov_5.34988 (+1)       NLLYEKEITSSILGYHLMRNIAKETIEILTLYKSHNIKLYVYNPLYPAI 289
NODE_1015_length_1915_cov_3.75806-RC (+1)  ------------------------------------------------- 638
NODE_3618_length_1260_cov_4.38921-RC (+2)  ------------------------------------------------- 419
NODE_24337_length_436_cov_8.89501 (+1)     ------------------------------------------------- 23
Consensus                                  -------------------------------------------------
Conservation (100% / 0%)
Sequence logo (4.3bits / 0.0bits)          NLLYEKEITSSILGYHLMRNIAKETIEILTLYKSHNIKLY[illegible]YNPLYPAI

                                                  3,100                     3,120
AB719397 (+1)                              QAMKSDSLASWVMLNPFPPEWIVGDKPESTWNKFKTNVAKWIVNLSDTI 3136
NODE_4_length_8339_cov_8.1445 (+2)         ------------------------------------------------- 2779
NODE_61_length_4588_cov_5.34988 (+1)       QAMKSNSLASWVMLNPFPPEWIVGDKPESTWNKFKTNVAKWIVNLSDTI 338
NODE_1015_length_1915_cov_3.75806-RC (+1)  ------------------------------------------------- 638
NODE_3618_length_1260_cov_4.38921-RC (+2)  ------------------------------------------------- 419
NODE_24337_length_436_cov_8.89501 (+1)     ------------------------------------------------- 23
Consensus                                  -------------------------------------------------
Conservation (100% / 0%)
Sequence logo (4.3bits / 0.0bits)          QAMKS[illegible]SLASWVMLNPFPPEWIVGDKPESTWNKFKTNVAKWIVNLSDTI

                                             3,140                3,160                3,180
AB719397 (+1)                              IRVDLNLPEHQSGTYQELTPMVCCPAELLPINDISNVICCGSLTMLNYY 3185
NODE_4_length_8339_cov_8.1445 (+2)         ------------------------------------------------- 2779
NODE_61_length_4588_cov_5.34988 (+1)       IRVDLNLPEHQSGTYQELTPMVCCPAELLPINDISNVICCGSLTMLNYY 387
NODE_1015_length_1915_cov_3.75806-RC (+1)  ------------------------------------------------- 638
NODE_3618_length_1260_cov_4.38921-RC (+2)  ------------------------------------------------- 419
NODE_24337_length_436_cov_8.89501 (+1)     ------------------------------------------------- 23
Consensus                                  -------------------------------------------------
Conservation (100% / 0%)
Sequence logo (4.3bits / 0.0bits)          IRVDLNLPEHQSGTYQELTPMVCCPAELLPINDISNVICCGSLTMLNYY

                                                    3,200                     3,220
AB719397 (+1)                              TDENFKTNFDSKAVLVSFGSCNIGVDTQLIIKILLICIKQGYKVIFHDG 3234
NODE_4_length_8339_cov_8.1445 (+2)         ------------------------------------------------- 2779
NODE_61_length_4588_cov_5.34988 (+1)       TDENFKTNFDSKAVLVSFGSCNIGVDAQLIIKILLICIKQGYKVIFHDG 436
NODE_1015_length_1915_cov_3.75806-RC (+1)  ------------------------------------------------- 638
NODE_3618_length_1260_cov_4.38921-RC (+2)  ------------------------------------------------- 419
NODE_24337_length_436_cov_8.89501 (+1)     ------------------------------------------------- 23
Consensus                                  -------------------------------------------------
Conservation (100% / 0%)
Sequence logo (4.3bits / 0.0bits)          TDENFKTNFDSKAVLVSFGSCNIGVD[illegible]QLIIKILLICIKQGYKVIFHDG
```

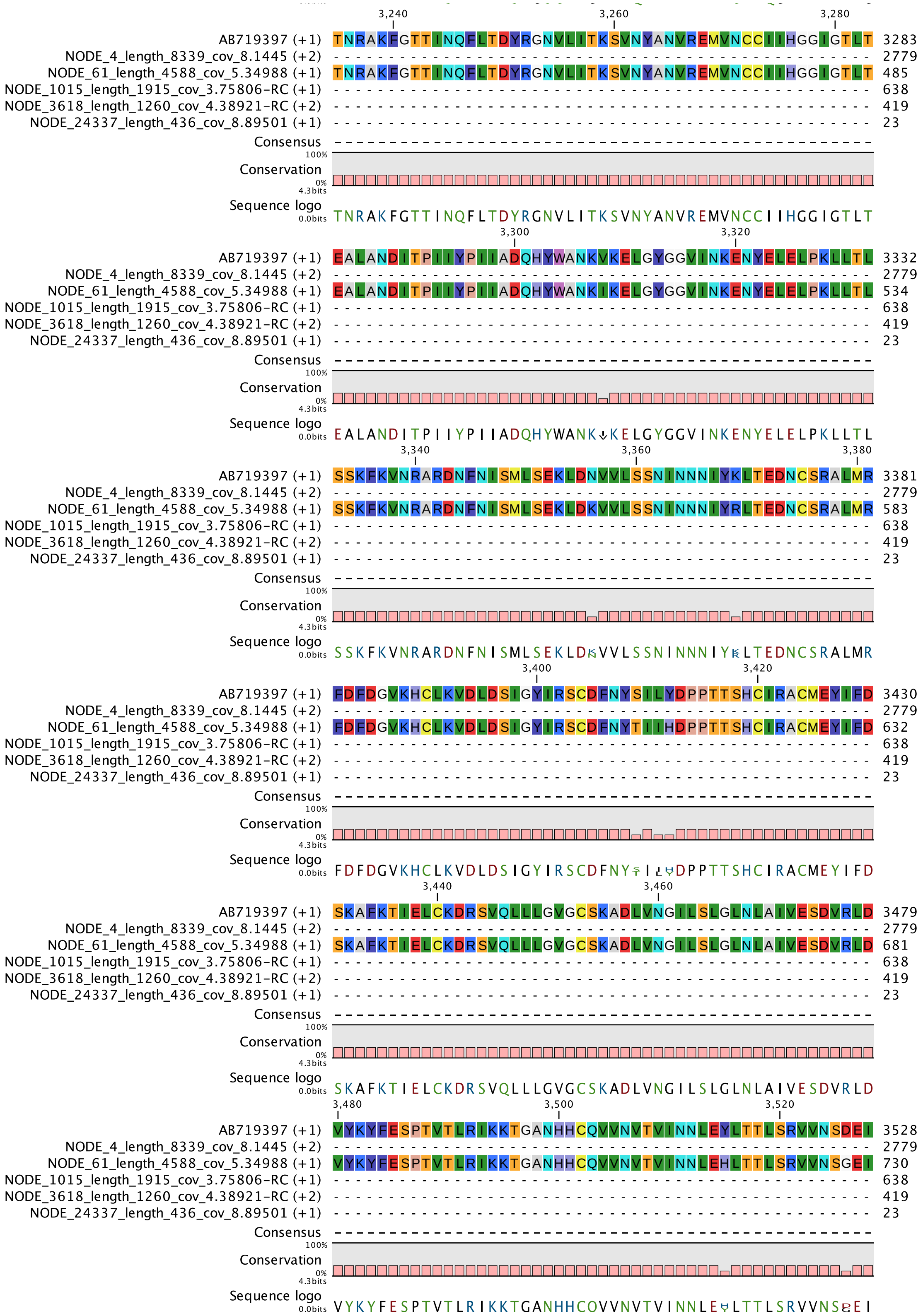
3,240 3,260 3,280
AB719397 (+1) TNRAKFGTTINQFLTDYRGNVLITKSVNYANVREMVNCCIIHGGIGTLT 3283
NODE_4_length_8339_cov_8.1445 (+2) 2779
NODE_61_length_4588_cov_5.34988 (+1) TNRAKFGTTINQFLTDYRGNVLITKSVNYANVREMVNCCIIHGGIGTLT 485
NODE_1015_length_1915_cov_3.75806-RC (+1) 638
NODE_3618_length_1260_cov_4.38921-RC (+2) 419
NODE_24337_length_436_cov_8.89501 (+1) 23
Consensus
Conservation
Sequence logo
3,300 3,320
AB719397 (+1) EALANDITPIIYPIIADQHYWANKVKELGYGGVINKENYELELPKLLTL 3332
NODE_4_length_8339_cov_8.1445 (+2) 2779
NODE_61_length_4588_cov_5.34988 (+1) EALANDITPIIYPIIADQHYWANKIKELGYGGVINKENYELELPKLLTL 534
NODE_1015_length_1915_cov_3.75806-RC (+1) 638
NODE_3618_length_1260_cov_4.38921-RC (+2) 419
NODE_24337_length_436_cov_8.89501 (+1) 23
Consensus
Conservation
Sequence logo
3,340 3,360 3,380
AB719397 (+1) SSKFKVNRARDNFNISMLSEKLDNVVLSSNINNNIYKLTEDNCSRALMR 3381
NODE_4_length_8339_cov_8.1445 (+2) 2779
NODE_61_length_4588_cov_5.34988 (+1) SSKFKVNRARDNFNISMLSEKLDKVVLSSNINNNIYRLTEDNCSRALMR 583
NODE_1015_length_1915_cov_3.75806-RC (+1) 638
NODE_3618_length_1260_cov_4.38921-RC (+2) 419
NODE_24337_length_436_cov_8.89501 (+1) 23
Consensus
Conservation
Sequence logo
3,400 3,420
AB719397 (+1) FDFDGVKHCLKVDLDSIGYIRSCDFNYSILYDPPTTSHCIRACMEYIFD 3430
NODE_4_length_8339_cov_8.1445 (+2) 2779
NODE_61_length_4588_cov_5.34988 (+1) FDFDGVKHCLKVDLDSIGYIRSCDFNYTIIHDPPTTSHCIRACMEYIFD 632
NODE_1015_length_1915_cov_3.75806-RC (+1) 638
NODE_3618_length_1260_cov_4.38921-RC (+2) 419
NODE_24337_length_436_cov_8.89501 (+1) 23
Consensus
Conservation
Sequence logo
3,440 3,460
AB719397 (+1) SKAFKTIELCKDRSVQLLLGVGCSKADLVNGILSLGLNLAIVESDVRLD 3479
NODE_4_length_8339_cov_8.1445 (+2) 2779
NODE_61_length_4588_cov_5.34988 (+1) SKAFKTIELCKDRSVQLLLGVGCSKADLVNGILSLGLNLAIVESDVRLD 681
NODE_1015_length_1915_cov_3.75806-RC (+1) 638
NODE_3618_length_1260_cov_4.38921-RC (+2) 419
NODE_24337_length_436_cov_8.89501 (+1) 23
Consensus
Conservation
Sequence logo
3,480 3,500 3,520
AB719397 (+1) VYKYFESPTVTLRIKKTGANHHCQVVNVTVINNLEYLTTLSRVVNSDEI 3528
NODE_4_length_8339_cov_8.1445 (+2) 2779
NODE_61_length_4588_cov_5.34988 (+1) VYKYFESPTVTLRIKKTGANHHCQVVNVTVINNLEHLTTLSRVVNSGEI 730
NODE_1015_length_1915_cov_3.75806-RC (+1) 638
NODE_3618_length_1260_cov_4.38921-RC (+2) 419
NODE_24337_length_436_cov_8.89501 (+1) 23
Consensus
Conservation
Sequence logo

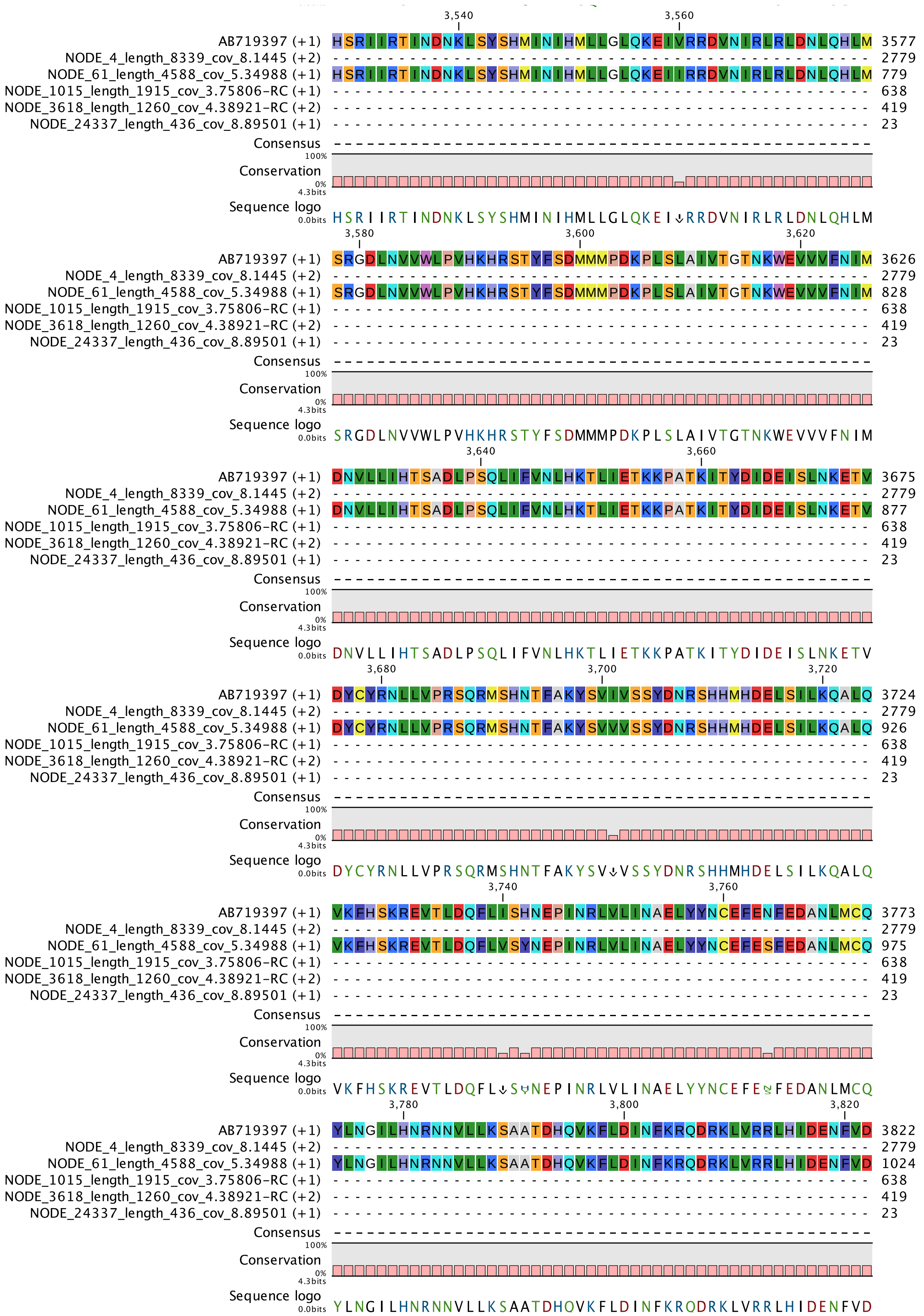
AB719397 (+1) HSRIIRTINDNKLSYSHMINIHMLLGLQKEIVRRDVNIRLRLDNLQHLM 3577
NODE_4_length_8339_cov_8.1445 (+2) 2779
NODE_61_length_4588_cov_5.34988 (+1) HSRIIRTINDNKLSYSHMINIHMLLGLQKEIIRRDVNIRLRLDNLQHLM 779
NODE_1015_length_1915_cov_3.75806-RC (+1) 638
NODE_3618_length_1260_cov_4.38921-RC (+2) 419
NODE_24337_length_436_cov_8.89501 (+1) 23
Consensus
Conservation
Sequence logo
AB719397 (+1) SRGDLNVVWLPVHKHRSTYFSDMMMPDKPLSLAIVTGTNKWEVVVFNIM 3626
NODE_61_length_4588_cov_5.34988 (+1) SRGDLNVVWLPVHKHRSTYFSDMMMPDKPLSLAIVTGTNKWEVVVFNIM 828
AB719397 (+1) DNVLLIHTSADLPSQLIFVNLHKTLIETKKPATKITYDIDEISLNKETV 3675
NODE_61_length_4588_cov_5.34988 (+1) DNVLLIHTSADLPSQLIFVNLHKTLIETKKPATKITYDIDEISLNKETV 877
AB719397 (+1) DYCYRNLLVPRSQRMSHNTFAKYSVIVSSYDNRSHHMHDELSILKQALQ 3724
NODE_61_length_4588_cov_5.34988 (+1) DYCYRNLLVPRSQRMSHNTFAKYSVVVSSYDNRSHHMHDELSILKQALQ 926
AB719397 (+1) VKFHSKREVTLDQFLISHNEPINRLVLINAELYYNCEFENFEDANLMCQ 3773
NODE_61_length_4588_cov_5.34988 (+1) VKFHSKREVTLDQFLVSYNEPINRLVLINAELYYNCEFESFEDANLMCQ 975
AB719397 (+1) YLNGILHNRNNVLLKSAATDHQVKFLDINFKRQDRKLVRRLHIDENFVD 3822
NODE_61_length_4588_cov_5.34988 (+1) YLNGILHNRNNVLLKSAATDHQVKFLDINFKRQDRKLVRRLHIDENFVD 1024

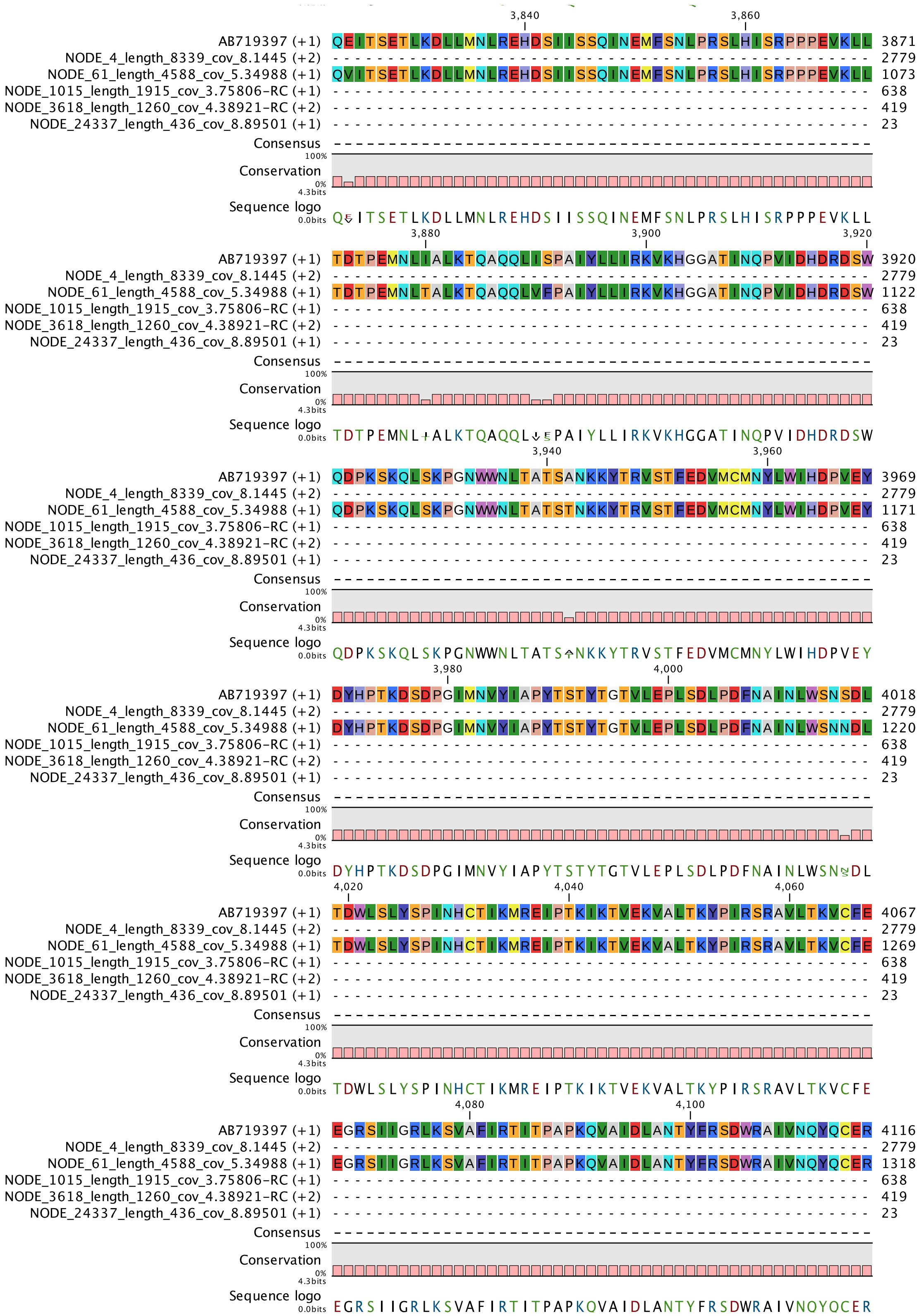
3,840 3,860
AB719397 (+1) QEITSETLKDLLMNLREHDSIISSQINEMFSNLPRSLHISRPPPEVKLL 3871
NODE_4_length_8339_cov_8.1445 (+2) 2779
NODE_61_length_4588_cov_5.34988 (+1) QVITSETLKDLLMNLREHDSIISSQINEMFSNLPRSLHISRPPPEVKLL 1073
NODE_1015_length_1915_cov_3.75806-RC (+1) 638
NODE_3618_length_1260_cov_4.38921-RC (+2) 419
NODE_24337_length_436_cov_8.89501 (+1) 23
Consensus
Conservation
Sequence logo
3,880 3,900 3,920
AB719397 (+1) TDTPEMNLIALKTQAQQLISPAIYLLIRKVKHGGATINQPVIDHDRDSW 3920
NODE_4_length_8339_cov_8.1445 (+2) 2779
NODE_61_length_4588_cov_5.34988 (+1) TDTPEMNLTALKTQAQQLVFPAIYLLIRKVKHGGATINQPVIDHDRDSW 1122
NODE_1015_length_1915_cov_3.75806-RC (+1) 638
NODE_3618_length_1260_cov_4.38921-RC (+2) 419
NODE_24337_length_436_cov_8.89501 (+1) 23
Consensus
Conservation
Sequence logo
3,940 3,960
AB719397 (+1) QDPKSKQLSKPGNWWNLTATSANKKYTRVSTFEDVMCMNYLWIHDPVEY 3969
NODE_4_length_8339_cov_8.1445 (+2) 2779
NODE_61_length_4588_cov_5.34988 (+1) QDPKSKQLSKPGNWWNLTATSTNKKYTRVSTFEDVMCMNYLWIHDPVEY 1171
NODE_1015_length_1915_cov_3.75806-RC (+1) 638
NODE_3618_length_1260_cov_4.38921-RC (+2) 419
NODE_24337_length_436_cov_8.89501 (+1) 23
Consensus
Conservation
Sequence logo
3,980 4,000
AB719397 (+1) DYHPTKDSDPGIMNVYIAPYTSTYTGTVLEPLSDLPDFNAINLWSNSDL 4018
NODE_4_length_8339_cov_8.1445 (+2) 2779
NODE_61_length_4588_cov_5.34988 (+1) DYHPTKDSDPGIMNVYIAPYTSTYTGTVLEPLSDLPDFNAINLWSNNDL 1220
NODE_1015_length_1915_cov_3.75806-RC (+1) 638
NODE_3618_length_1260_cov_4.38921-RC (+2) 419
NODE_24337_length_436_cov_8.89501 (+1) 23
Consensus
Conservation
Sequence logo
4,020 4,040 4,060
AB719397 (+1) TDWLSLYSPINHCTIKMREIPTKIKTVEKVALTKYPIRSRAVLTKVCFE 4067
NODE_4_length_8339_cov_8.1445 (+2) 2779
NODE_61_length_4588_cov_5.34988 (+1) TDWLSLYSPINHCTIKMREIPTKIKTVEKVALTKYPIRSRAVLTKVCFE 1269
NODE_1015_length_1915_cov_3.75806-RC (+1) 638
NODE_3618_length_1260_cov_4.38921-RC (+2) 419
NODE_24337_length_436_cov_8.89501 (+1) 23
Consensus
Conservation
Sequence logo
4,080 4,100
AB719397 (+1) EGRSIIGRLKSVAFIRTITPAPKQVAIDLANTYFRSDWRAIVNQYQCER 4116
NODE_4_length_8339_cov_8.1445 (+2) 2779
NODE_61_length_4588_cov_5.34988 (+1) EGRSIIGRLKSVAFIRTITPAPKQVAIDLANTYFRSDWRAIVNQYQCER 1318
NODE_1015_length_1915_cov_3.75806-RC (+1) 638
NODE_3618_length_1260_cov_4.38921-RC (+2) 419
NODE_24337_length_436_cov_8.89501 (+1) 23
Consensus
Conservation
Sequence logo

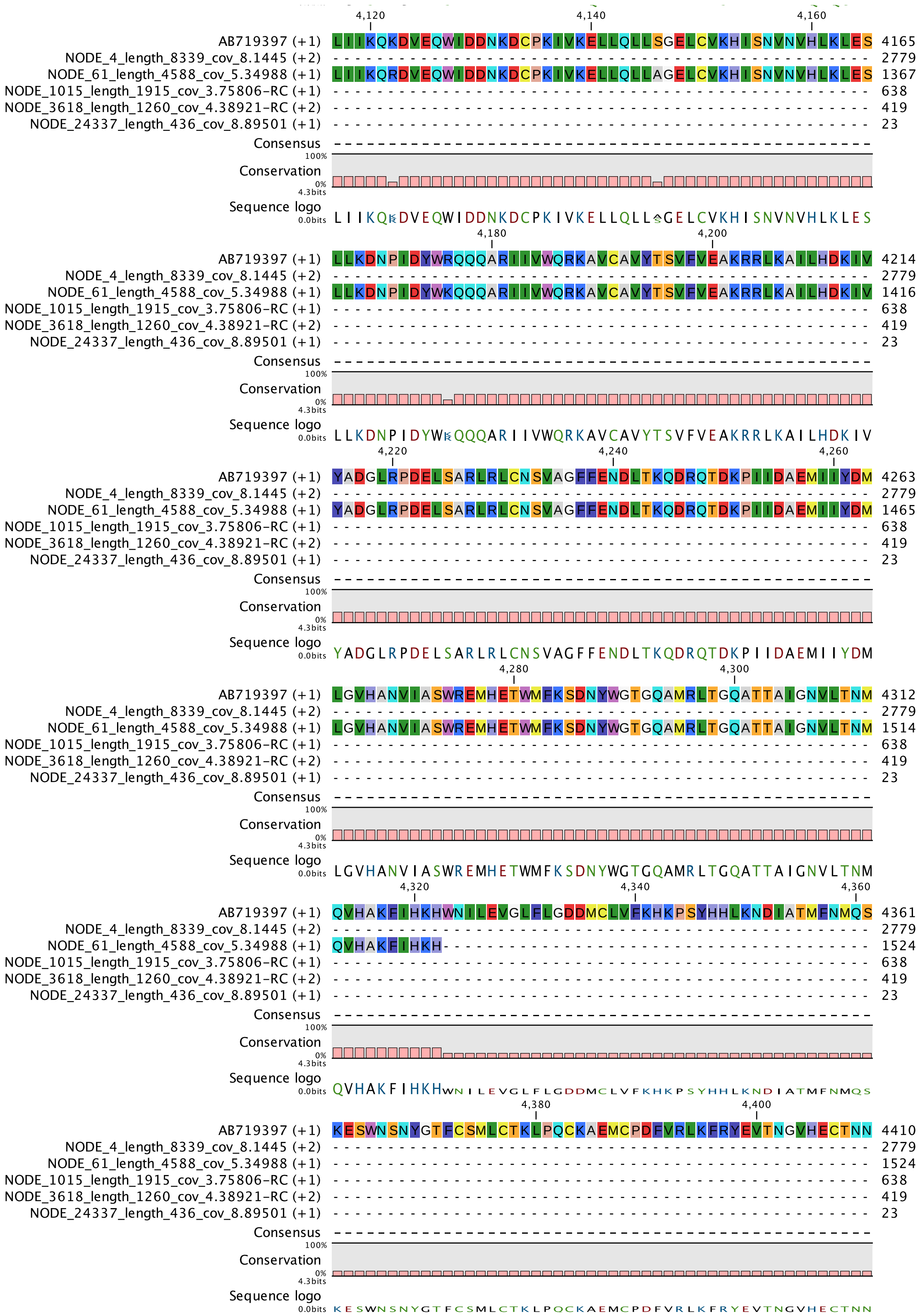
4,120 4,140 4,160
AB719397 (+1) LIIKQKDVEQWIDDNKDCPKIVKELLQLLSGELCVKHISNVNVHLKLES 4165
NODE_4_length_8339_cov_8.1445 (+2) 2779
NODE_61_length_4588_cov_5.34988 (+1) LIIKQRDVEQWIDDNKDCPKIVKELLQLLAGELCVKHISNVNVHLKLES 1367
NODE_1015_length_1915_cov_3.75806-RC (+1) 638
NODE_3618_length_1260_cov_4.38921-RC (+2) 419
NODE_24337_length_436_cov_8.89501 (+1) 23
Consensus
Conservation
Sequence logo
4,180 4,200
AB719397 (+1) LLKDNPIDYWRQQQARIIVWQRKAVCAVYTSVFVEAKRRLKAILHDKIV 4214
NODE_4_length_8339_cov_8.1445 (+2) 2779
NODE_61_length_4588_cov_5.34988 (+1) LLKDNPIDYWKQQQARIIVWQRKAVCAVYTSVFVEAKRRLKAILHDKIV 1416
NODE_1015_length_1915_cov_3.75806-RC (+1) 638
NODE_3618_length_1260_cov_4.38921-RC (+2) 419
NODE_24337_length_436_cov_8.89501 (+1) 23
Consensus
Conservation
Sequence logo
4,220 4,240 4,260
AB719397 (+1) YADGLRPDELSARLRLCNSVAGFFENDLTKQDRQTDKPIIDAEMIIYDM 4263
NODE_4_length_8339_cov_8.1445 (+2) 2779
NODE_61_length_4588_cov_5.34988 (+1) YADGLRPDELSARLRLCNSVAGFFENDLTKQDRQTDKPIIDAEMIIYDM 1465
NODE_1015_length_1915_cov_3.75806-RC (+1) 638
NODE_3618_length_1260_cov_4.38921-RC (+2) 419
NODE_24337_length_436_cov_8.89501 (+1) 23
Consensus
Conservation
Sequence logo
4,280 4,300
AB719397 (+1) LGVHANVIASWREMHETWMFKSDNYWGTGQAMRLTGQATTAIGNVLTNM 4312
NODE_4_length_8339_cov_8.1445 (+2) 2779
NODE_61_length_4588_cov_5.34988 (+1) LGVHANVIASWREMHETWMFKSDNYWGTGQAMRLTGQATTAIGNVLTNM 1514
NODE_1015_length_1915_cov_3.75806-RC (+1) 638
NODE_3618_length_1260_cov_4.38921-RC (+2) 419
NODE_24337_length_436_cov_8.89501 (+1) 23
Consensus
Conservation
Sequence logo
4,320 4,340 4,360
AB719397 (+1) QVHAKFIHKHWNILEVGLFLGDDMCLVFKHKPSYHHLKNDIATMFNMQS 4361
NODE_4_length_8339_cov_8.1445 (+2) 2779
NODE_61_length_4588_cov_5.34988 (+1) QVHAKFIHKH 1524
NODE_1015_length_1915_cov_3.75806-RC (+1) 638
NODE_3618_length_1260_cov_4.38921-RC (+2) 419
NODE_24337_length_436_cov_8.89501 (+1) 23
Consensus
Conservation
Sequence logo
4,380 4,400
AB719397 (+1) KESWNSNYGTFCSMLCTKLPQCKAEMCPDFVRLKFRYEVTNGVHECTNN 4410
NODE_4_length_8339_cov_8.1445 (+2) 2779
NODE_61_length_4588_cov_5.34988 (+1) 1524
NODE_1015_length_1915_cov_3.75806-RC (+1) 638
NODE_3618_length_1260_cov_4.38921-RC (+2) 419
NODE_24337_length_436_cov_8.89501 (+1) 23
Consensus
Conservation
Sequence logo

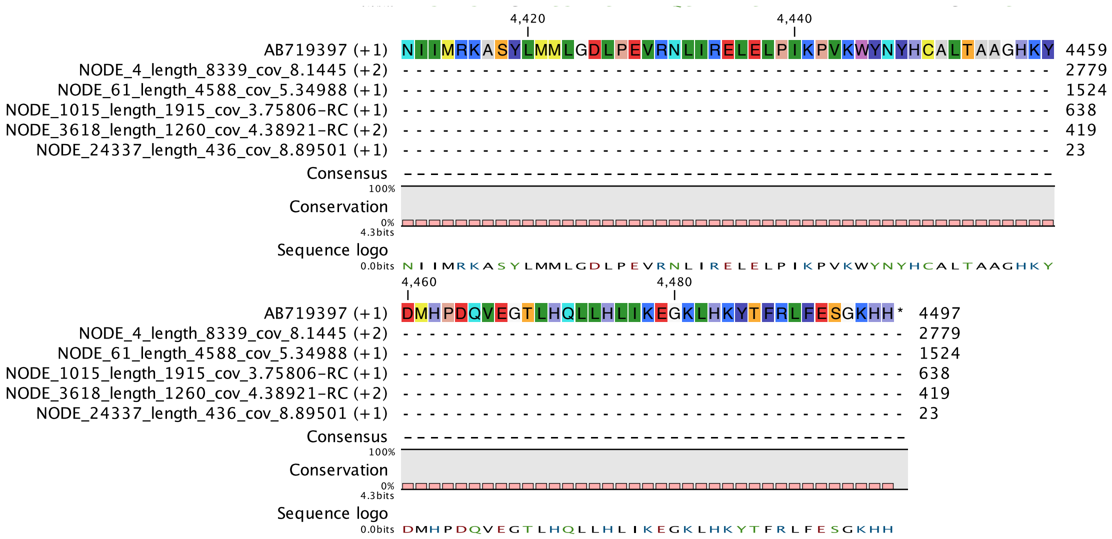

Supplemental Figure S2. Sequence alignment of the amino acids of the PvEV1 polyprotein (accession number AB719397: Okada et al., 2013) and sequence from the Kenyan isolates of PvEV1. The sequences were aligned by the CLC Sequence Viewer 8.0 application.
