# Supplementary material for: Metagenomic Analysis of Plant Virus Occurrence in Common Bean (Phaseolus vulgaris) in Central Kenya
Source: Front Microbiol. 2018 Dec 7;9:2939. doi: 10.3389/fmicb.2018.02939 (PMC6293961; doi:10.3389/fmicb.2018.02939)
Supplement: Supplementary file 2 [file Data_Sheet_2.PDF]

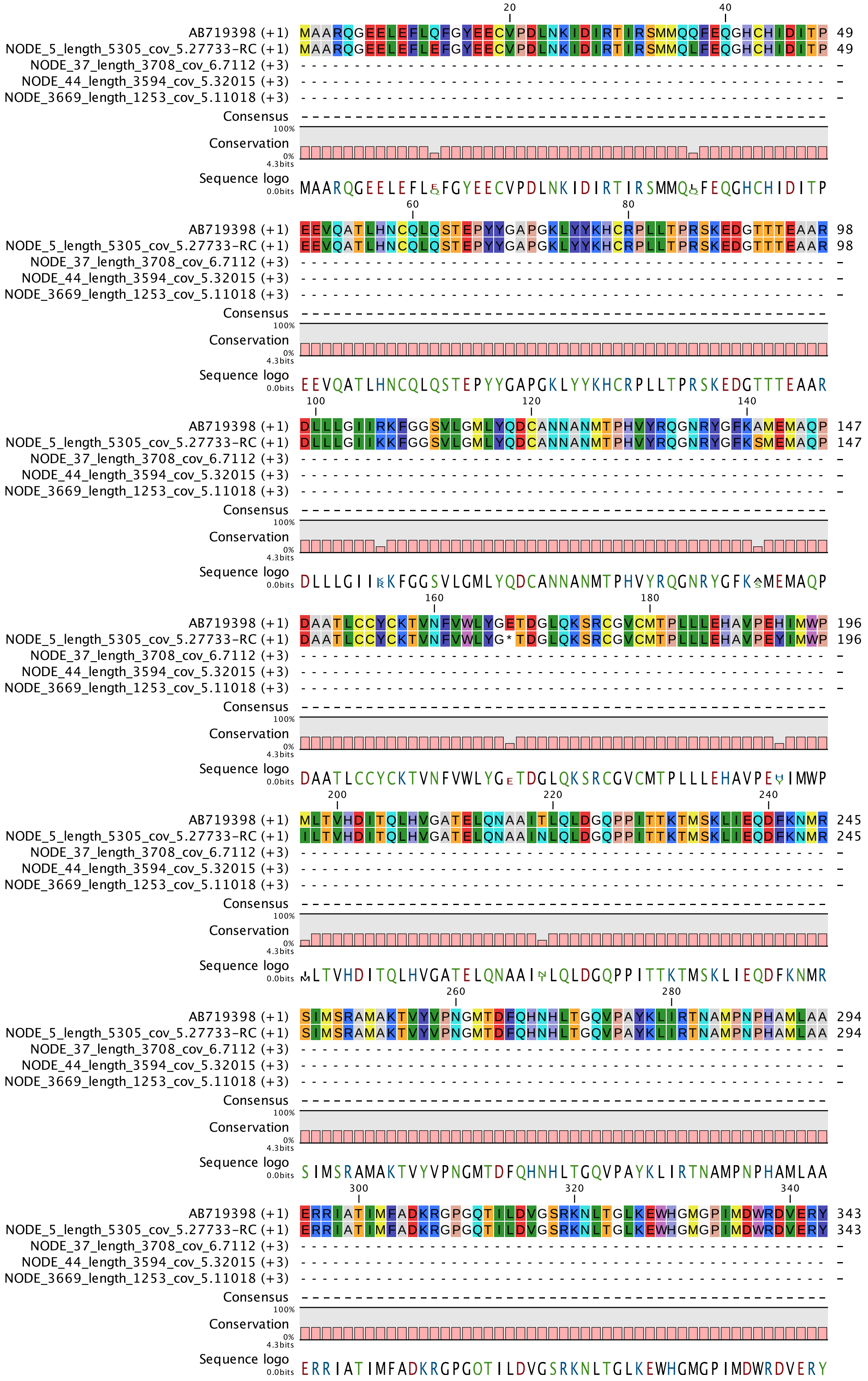
20
40
AB719398 (+1) MAARQGEELEFLQFGYEECVPDLNKIDIRTIRSMMQQFEQGHCHIDITP 49
NODE_5_length_5305_cov_5.27733-RC (+1) MAARQGEELEFLEFGYEECVPDLNKIDIRTIRSMMQLFEQGHCHIDITP 49
NODE_37_length_3708_cov_6.7112 (+3)
NODE_44_length_3594_cov_5.32015 (+3)
NODE_3669_length_1253_cov_5.11018 (+3)
Consensus
100%
Conservation
0%
4.3bits
Sequence logo
0.0bits
60
80
AB719398 (+1) EEVQATLHNCQLQSTEPYYGAPGKLYYKHCRPLLTPRSKEDGTTTEAAR 98
NODE_5_length_5305_cov_5.27733-RC (+1) EEVQATLHNCQLQSTEPYYGAPGKLYYKHCRPLLTPRSKEDGTTTEAAR 98
100
120
140
AB719398 (+1) DLLLGIIRKFGGSVLGMLYQDCANNANMTPHVYRQGNRYGFKAMEMAQP 147
NODE_5_length_5305_cov_5.27733-RC (+1) DLLLGIIKKFGGSVLGMLYQDCANNANMTPHVYRQGNRYGFKSMEMAQP 147
160
180
AB719398 (+1) DAATLCCYCKTVNFVWLYGETDGLQKSRCGVCMTPLLLEHAVPEHIMWP 196
NODE_5_length_5305_cov_5.27733-RC (+1) DAATLCCYCKTVNFVWLYG*TDGLQKSRCGVCMTPLLLEHAVPEYIMWP 196
200
220
240
AB719398 (+1) MLTVHDITQLHVGATELQNAAITLQLDGQPPITTKTMSKLIEQDFKNMR 245
NODE_5_length_5305_cov_5.27733-RC (+1) ILTVHDITQLHVGATELQNAAINLQLDGQPPITTKTMSKLIEQDFKNMR 245
260
280
AB719398 (+1) SIMSRAMAKTVYVPNGMTDFQHNHLTGQVPAYKLIRTNAMPNPHAMLAA 294
NODE_5_length_5305_cov_5.27733-RC (+1) SIMSRAMAKTVYVPNGMTDFQHNHLTGQVPAYKLIRTNAMPNPHAMLAA 294
300
320
340
AB719398 (+1) ERRIATIMFADKRGPGQTILDVGSRKNLTGLKEWHGMGPIMDWRDVERY 343
NODE_5_length_5305_cov_5.27733-RC (+1) ERRIATIMFADKRGPGQTILDVGSRKNLTGLKEWHGMGPIMDWRDVERY 343

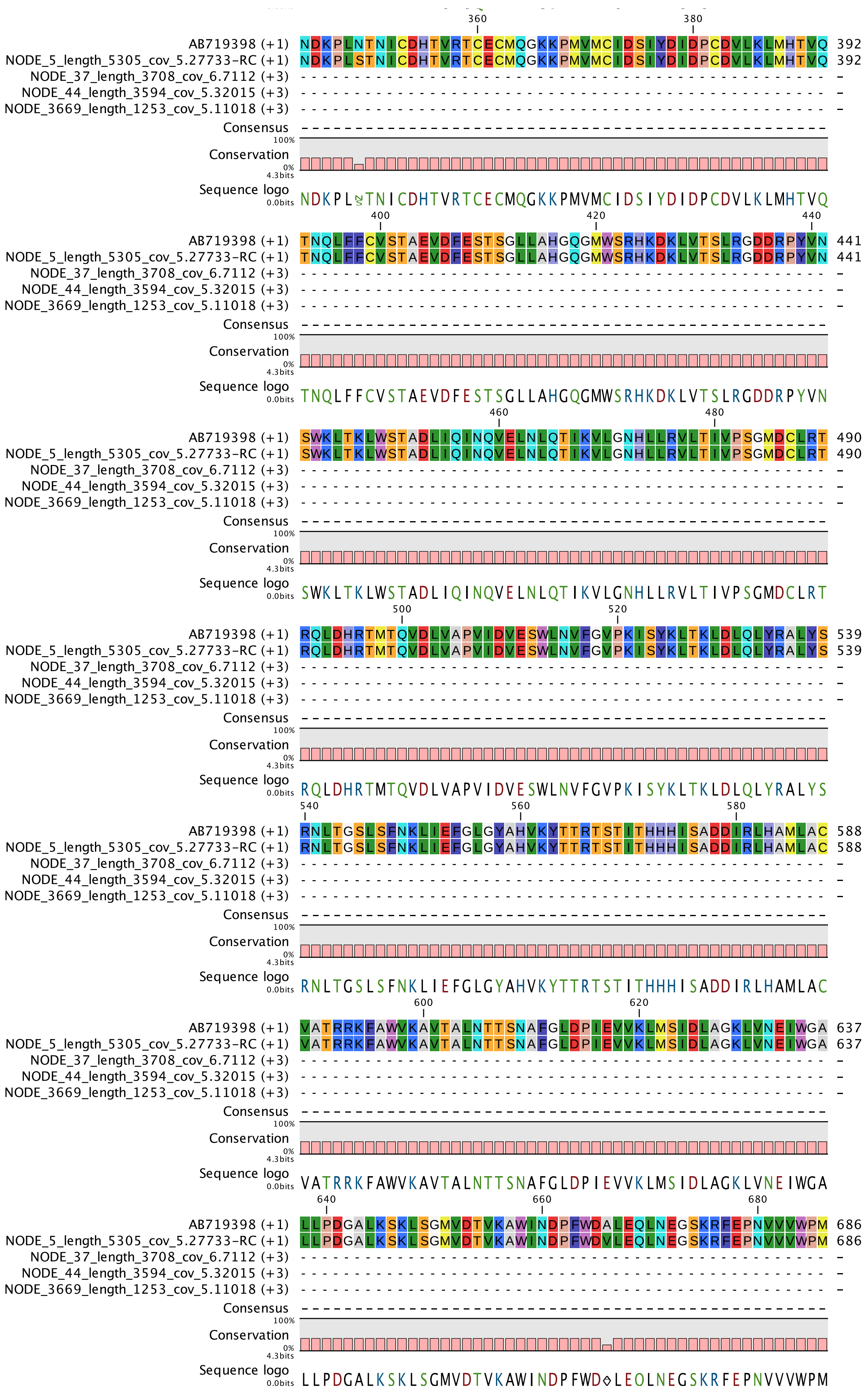

360 380
AB719398 (+1) NDKPLNTNICDHTVRTCECMQGKKPMVMCIDSIYDIDPCDVLKLMHTVQ 392
NODE_5_length_5305_cov_5.27733-RC (+1) NDKPLSTNICDHTVRTCECMQGKKPMVMCIDSIYDIDPCDVLKLMHTVQ 392
NODE_37_length_3708_cov_6.7112 (+3)
NODE_44_length_3594_cov_5.32015 (+3)
NODE_3669_length_1253_cov_5.11018 (+3)
Consensus
Conservation
Sequence logo
400 420 440
AB719398 (+1) TNQLFFCVSTAEVDFESTSGLLAHGQGMWSRHKDKLVTSLRGDDRPYVN 441
NODE_5_length_5305_cov_5.27733-RC (+1) TNQLFFCVSTAEVDFESTSGLLAHGQGMWSRHKDKLVTSLRGDDRPYVN 441
460 480
AB719398 (+1) SWKLTKLWSTADLIQINQVELNLQTIKVLGNHLLRVLTIVPSGMDCLRT 490
NODE_5_length_5305_cov_5.27733-RC (+1) SWKLTKLWSTADLIQINQVELNLQTIKVLGNHLLRVLTIVPSGMDCLRT 490
500 520
AB719398 (+1) RQLDHRTMTQVDLVAPVIDVESWLNVFGVPKISYKLTKLDLQLYRALYS 539
NODE_5_length_5305_cov_5.27733-RC (+1) RQLDHRTMTQVDLVAPVIDVESWLNVFGVPKISYKLTKLDLQLYRALYS 539
540 560 580
AB719398 (+1) RNLTGSLSFNKLIEFGLGYAHVKYTTRTSTITHHHISADDIRLHAMLAC 588
NODE_5_length_5305_cov_5.27733-RC (+1) RNLTGSLSFNKLIEFGLGYAHVKYTTRTSTITHHHISADDIRLHAMLAC 588
600 620
AB719398 (+1) VATRRKFAWVKAVTALNTTSNAFGLDPIEVVKLMSIDLAGKLVNEIWGA 637
NODE_5_length_5305_cov_5.27733-RC (+1) VATRRKFAWVKAVTALNTTSNAFGLDPIEVVKLMSIDLAGKLVNEIWGA 637
640 660 680
AB719398 (+1) LLPDGALKSKLSGMVDTVKAWINDPFWDALEQLNEGSKRFEPNVVVWPM 686
NODE_5_length_5305_cov_5.27733-RC (+1) LLPDGALKSKLSGMVDTVKAWINDPFWDVLEQLNEGSKRFEPNVVVWPM 686

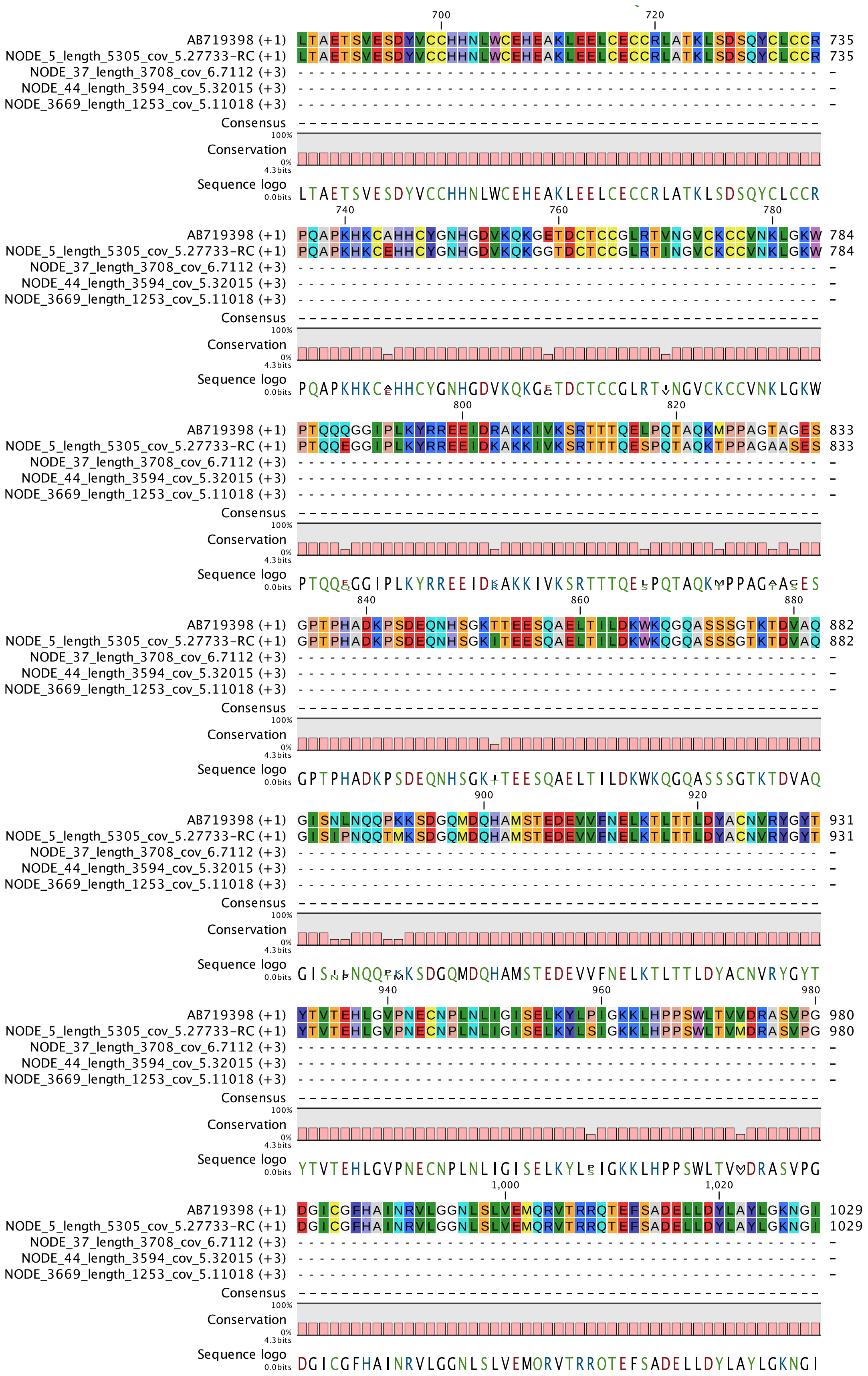
AB719398 (+1) LTAETSVESDYVCCHHNLWCEHEAKLEELCECCRLATKLSDSQYCLCCR 735
NODE_5_length_5305_cov_5.27733-RC (+1) LTAETSVESDYVCCHHNLWCEHEAKLEELCECCRLATKLSDSQYCLCCR 735
NODE_37_length_3708_cov_6.7112 (+3)
NODE_44_length_3594_cov_5.32015 (+3)
NODE_3669_length_1253_cov_5.11018 (+3)
Consensus
Conservation
Sequence logo
AB719398 (+1) PQAPKHKCAHHCYGNHGDVKQKGETDCTCCGLRTVNGVCKCCVNKLGKW 784
NODE_5_length_5305_cov_5.27733-RC (+1) PQAPKHKCEHHCYGNHGDVKQKGGTDCTCCGLRTINGVCKCCVNKLGKW 784
AB719398 (+1) PTQQQGGIPLKYRREEIDRAKKIVKSRTTTQELPQTAQKMPPAGTAGES 833
NODE_5_length_5305_cov_5.27733-RC (+1) PTQQEGGIPLKYRREEIDKAKKIVKSRTTTQESPQTAQKTPPAGAASES 833
AB719398 (+1) GPTPHADKPSDEQNHSGKTTEESQAELTILDKWKQGQASSSGTKTDVAQ 882
NODE_5_length_5305_cov_5.27733-RC (+1) GPTPHADKPSDEQNHSGKITEESQAELTILDKWKQGQASSSGTKTDVAQ 882
AB719398 (+1) GISNLNQQPKKSDGQMDQHAMSTEDEVVFNELKTLTTLDYACNVRYGYT 931
NODE_5_length_5305_cov_5.27733-RC (+1) GISIPNQQTMKSDGQMDQHAMSTEDEVVFNELKTLTTLDYACNVRYGYT 931
AB719398 (+1) YTVTEHLGVPNECNPLNLIGISELKYLPIGKKLHPPSWLTVVDRASVPG 980
NODE_5_length_5305_cov_5.27733-RC (+1) YTVTEHLGVPNECNPLNLIGISELKYLSIGKKLHPPSWLTVMDRASVPG 980
AB719398 (+1) DGICGFHAINRVLGGNLSLVEMQRVTRRQTEFSADELLDYLAYLGKNGI 1029
NODE_5_length_5305_cov_5.27733-RC (+1) DGICGFHAINRVLGGNLSLVEMQRVTRRQTEFSADELLDYLAYLGKNGI 1029

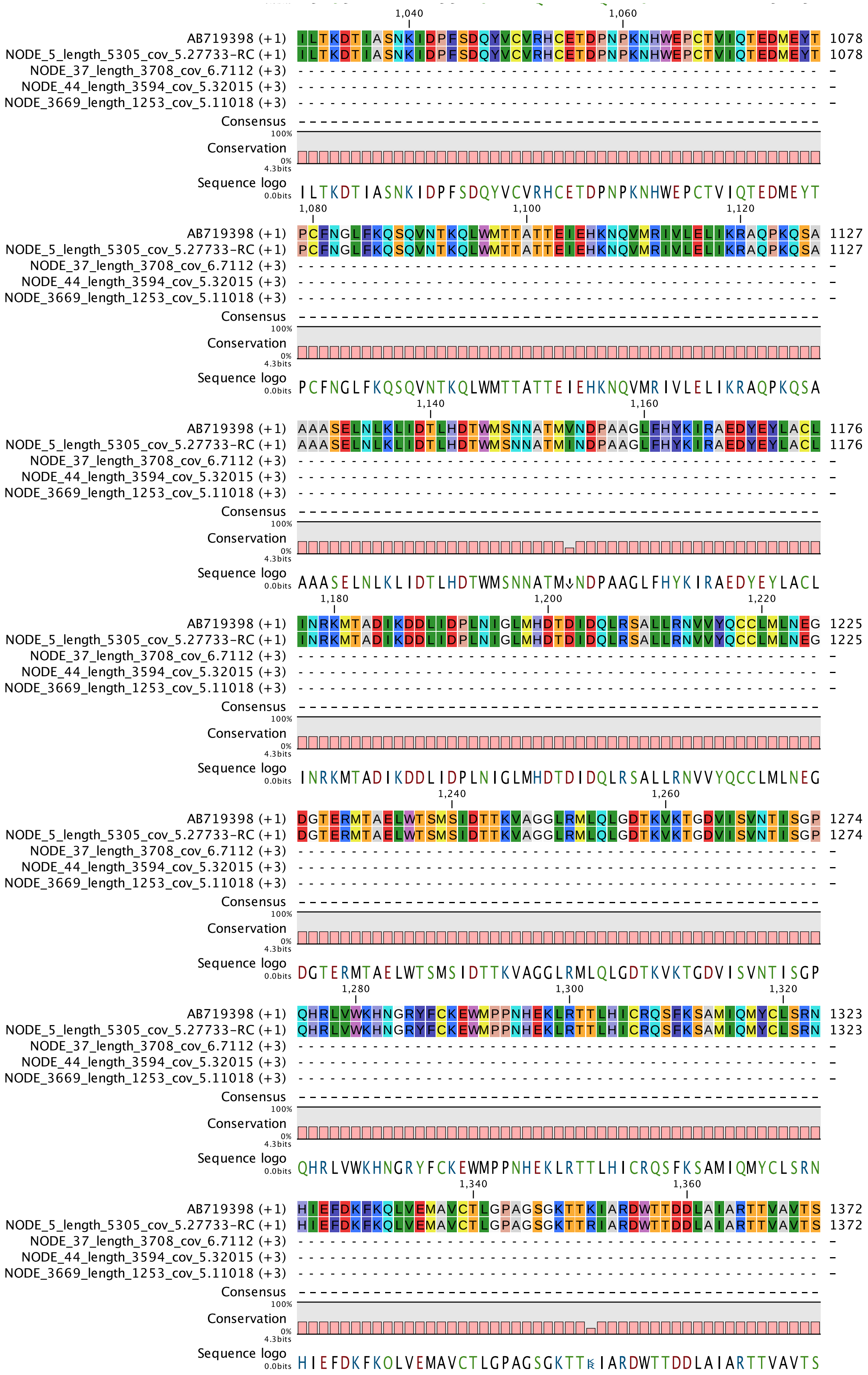
1,040
1,060
AB719398 (+1) ILTKDTIASNKIDPFSDQYVCVRHCETDPNPKNHWEPCTVIQTEDMEYT 1078
NODE_5_length_5305_cov_5.27733-RC (+1) ILTKDTIASNKIDPFSDQYVCVRHCETDPNPKNHWEPCTVIQTEDMEYT 1078
NODE_37_length_3708_cov_6.7112 (+3)
NODE_44_length_3594_cov_5.32015 (+3)
NODE_3669_length_1253_cov_5.11018 (+3)
Consensus
Conservation
Sequence logo
1,080
1,100
1,120
AB719398 (+1) PCFNGLFKQSQVNTKQLWMTTATTEIEHKNQVMRIVLELIKRAQPKQSA 1127
NODE_5_length_5305_cov_5.27733-RC (+1) PCFNGLFKQSQVNTKQLWMTTATTEIEHKNQVMRIVLELIKRAQPKQSA 1127
1,140
1,160
AB719398 (+1) AAASELNLKLIDTLHDTWMSNNATMVNDPAAGLFHYKIRAEDYEYLACL 1176
NODE_5_length_5305_cov_5.27733-RC (+1) AAASELNLKLIDTLHDTWMSNNATMINDPAAGLFHYKIRAEDYEYLACL 1176
1,180
1,200
1,220
AB719398 (+1) INRKMTADIKDDLIDPLNIGLMHDTDIDQLRSALLRNVVYQCCLMLNEG 1225
NODE_5_length_5305_cov_5.27733-RC (+1) INRKMTADIKDDLIDPLNIGLMHDTDIDQLRSALLRNVVYQCCLMLNEG 1225
1,240
1,260
AB719398 (+1) DGTERMTAELWTSMSIDTTKVAGGLRMLQLGDTKVKTGDVISVNTISGP 1274
NODE_5_length_5305_cov_5.27733-RC (+1) DGTERMTAELWTSMSIDTTKVAGGLRMLQLGDTKVKTGDVISVNTISGP 1274
1,280
1,300
1,320
AB719398 (+1) QHRLVWKHNGRYFCKEWMPPNHEKLRTTLHICRQSFKSAMIQMYCLSRN 1323
NODE_5_length_5305_cov_5.27733-RC (+1) QHRLVWKHNGRYFCKEWMPPNHEKLRTTLHICRQSFKSAMIQMYCLSRN 1323
1,340
1,360
AB719398 (+1) HIEFDKFKQLVEMAVCTLGPAGSGKTTKIARDWTTDDLAIARTTVAVTS 1372
NODE_5_length_5305_cov_5.27733-RC (+1) HIEFDKFKQLVEMAVCTLGPAGSGKTTRIARDWTTDDLAIARTTVAVTS 1372

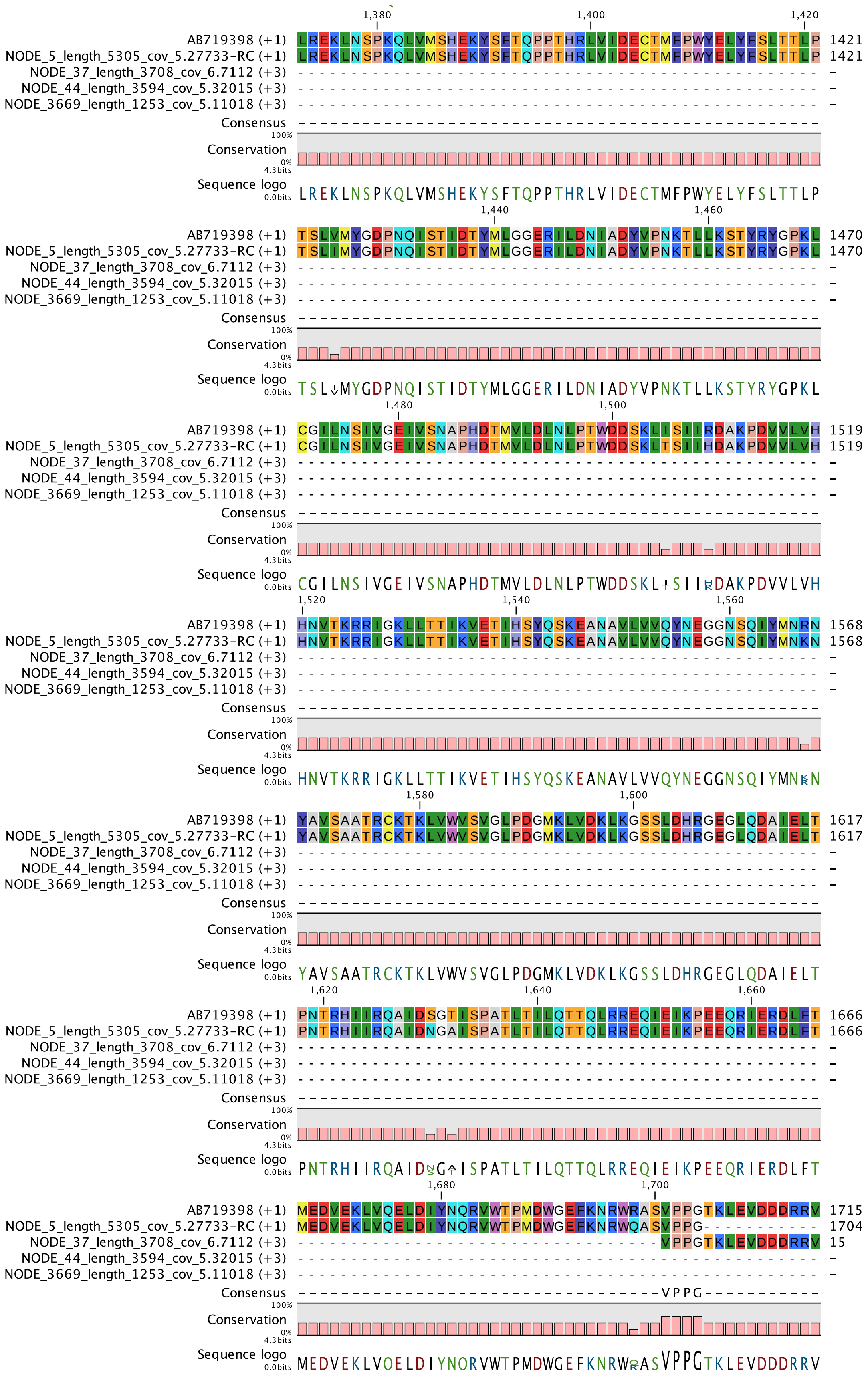
1,380 1,400 1,420
AB719398 (+1) LREKLNSPKQLVMSHEKYSFTQPPTHRLVIDECTMFPWYELYFSLTTLP 1421
NODE_5_length_5305_cov_5.27733-RC (+1) LREKLNSPKQLVMSHEKYSFTQPPTHRLVIDECTMFPWYELYFSLTTLP 1421
NODE_37_length_3708_cov_6.7112 (+3)
NODE_44_length_3594_cov_5.32015 (+3)
NODE_3669_length_1253_cov_5.11018 (+3)
Consensus
Conservation
Sequence logo
1,440 1,460
AB719398 (+1) TSLVMYGDPNQISTIDTYMLGGERILDNIADYVPNKTLLKSTYRYGPKL 1470
NODE_5_length_5305_cov_5.27733-RC (+1) TSLIMYGDPNQISTIDTYMLGGERILDNIADYVPNKTLLKSTYRYGPKL 1470
1,480 1,500
AB719398 (+1) CGILNSIVGEIVSNAPHDTMVLDLNLPTWDDSKLISIIRDAKPDVVLVH 1519
NODE_5_length_5305_cov_5.27733-RC (+1) CGILNSIVGEIVSNAPHDTMVLDLNLPTWDDSKLTSIIHDAKPDVVLVH 1519
1,520 1,540 1,560
AB719398 (+1) HNVTKRRIGKLLTTIKVETIHSYQSKEANAVLVVQYNEGGNSQIYMNRN 1568
NODE_5_length_5305_cov_5.27733-RC (+1) HNVTKRRIGKLLTTIKVETIHSYQSKEANAVLVVQYNEGGNSQIYMNKN 1568
1,580 1,600
AB719398 (+1) YAVSAATRCKTKLVWVSVGLPDGMKLVDKLKGSSLDHRGEGLQDAIELT 1617
NODE_5_length_5305_cov_5.27733-RC (+1) YAVSAATRCKTKLVWVSVGLPDGMKLVDKLKGSSLDHRGEGLQDAIELT 1617
1,620 1,640 1,660
AB719398 (+1) PNTRHIIRQAIDSGTISPATLTILQTTQLRREQIEIKPEEQRIERDLFT 1666
NODE_5_length_5305_cov_5.27733-RC (+1) PNTRHIIRQAIDNGAISPATLTILQTTQLRREQIEIKPEEQRIERDLFT 1666
1,680 1,700
AB719398 (+1) MEDVEKLVQELDIYNQRVWTPMDWGEFKNRWRASVPPGTKLEVDDDRRV 1715
NODE_5_length_5305_cov_5.27733-RC (+1) MEDVEKLVQELDIYNQRVWTPMDWGEFKNRWQASVPPG 1704
NODE_37_length_3708_cov_6.7112 (+3) VPPGTKLEVDDDRRV 15
Consensus VPPG

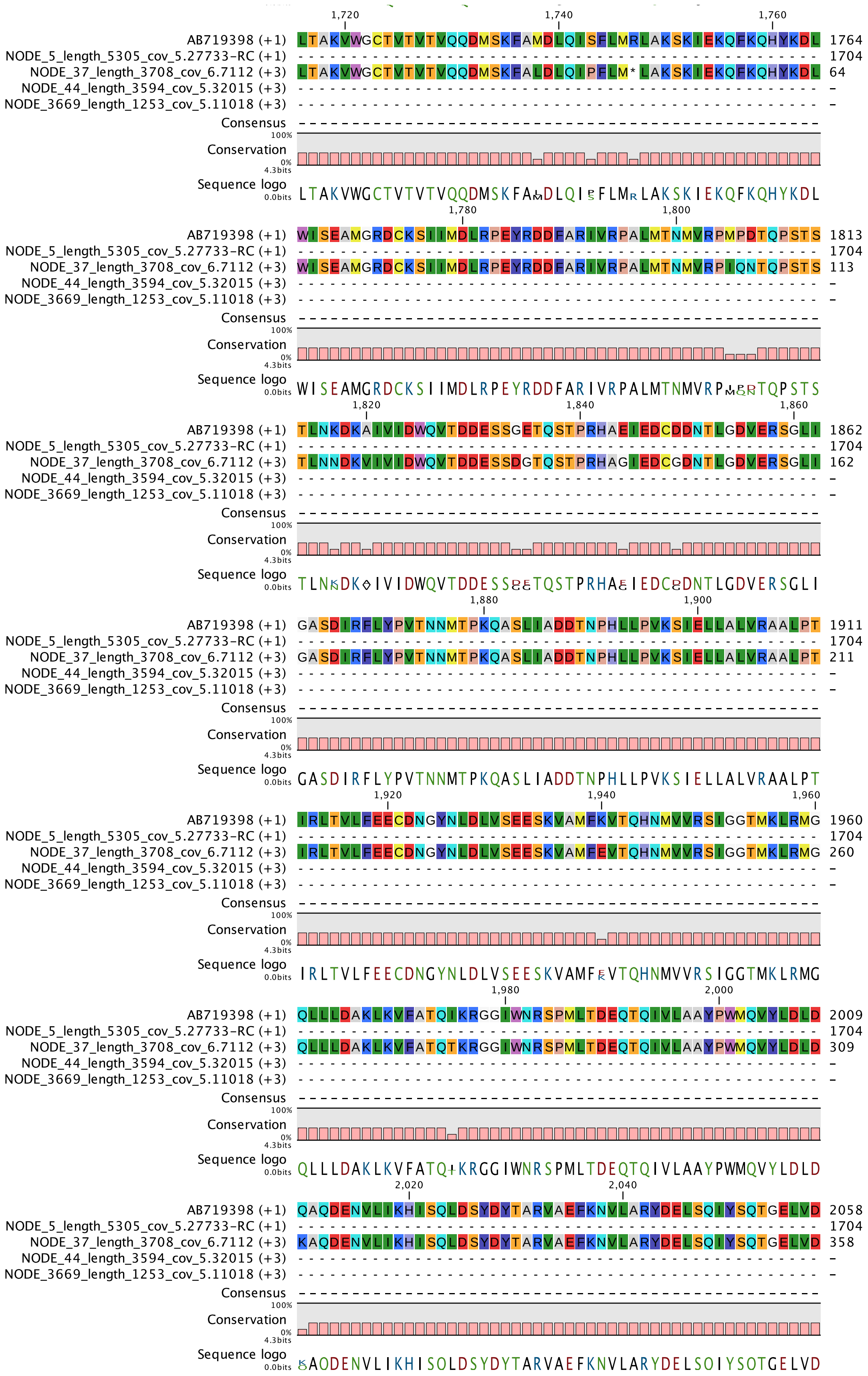
AB719398 (+1)
NODE_5_length_5305_cov_5.27733-RC (+1)
NODE_37_length_3708_cov_6.7112 (+3)
NODE_44_length_3594_cov_5.32015 (+3)
NODE_3669_length_1253_cov_5.11018 (+3)
Consensus
Conservation
Sequence logo
LTAKVWGCTVTVTVQQDMSKFAMDLQISFLMRLAKSKIEKQFKQHYKDL 1764
LTAKVWGCTVTVTVQQDMSKFALDLQIPFLM*LAKSKIEKQFKQHYKDL 64
WISEAMGRDCKSIIMDLRPEYRDDFARIVRPALMTNMVRPMPDTQPSTS 1813
WISEAMGRDCKSIIMDLRPEYRDDFARIVRPALMTNMVRPIQNTQPSTS 113
TLNKDKAIVIDWQVTDDESSGETQSTPRHAEIEDCDDNTLGDVERSGLI 1862
TLNNDKVIVIDWQVTDDESSDGTQSTPRHAGIEDCGDNTLGDVERSGLI 162
GASDIRFLYPVTNNMTPKQASLIADDTNPHLLPVKSIELLALVRAALPT 1911
GASDIRFLYPVTNNMTPKQASLIADDTNPHLLPVKSIELLALVRAALPT 211
IRLTVLFEECDNGYNLDLVSEESKVAMFKVTQHNMVVRSIGGTMKLRMG 1960
IRLTVLFEECDNGYNLDLVSEESKVAMFEVTQHNMVVRSIGGTMKLRMG 260
QLLLDAKLKVFATQIKRGGIWNRSPMLTDEQTQIVLAAYPWMQVYLDLD 2009
QLLLDAKLKVFATQTKRGGIWNRSPMLTDEQTQIVLAAYPWMQVYLDLD 309
QAQDENVLIKHISQLDSYDYTARVAEFKNVLARYDELSQIYSQTGELVD 2058
KAQDENVLIKHISQLDSYDYTARVAEFKNVLARYDELSQIYSQTGELVD 358

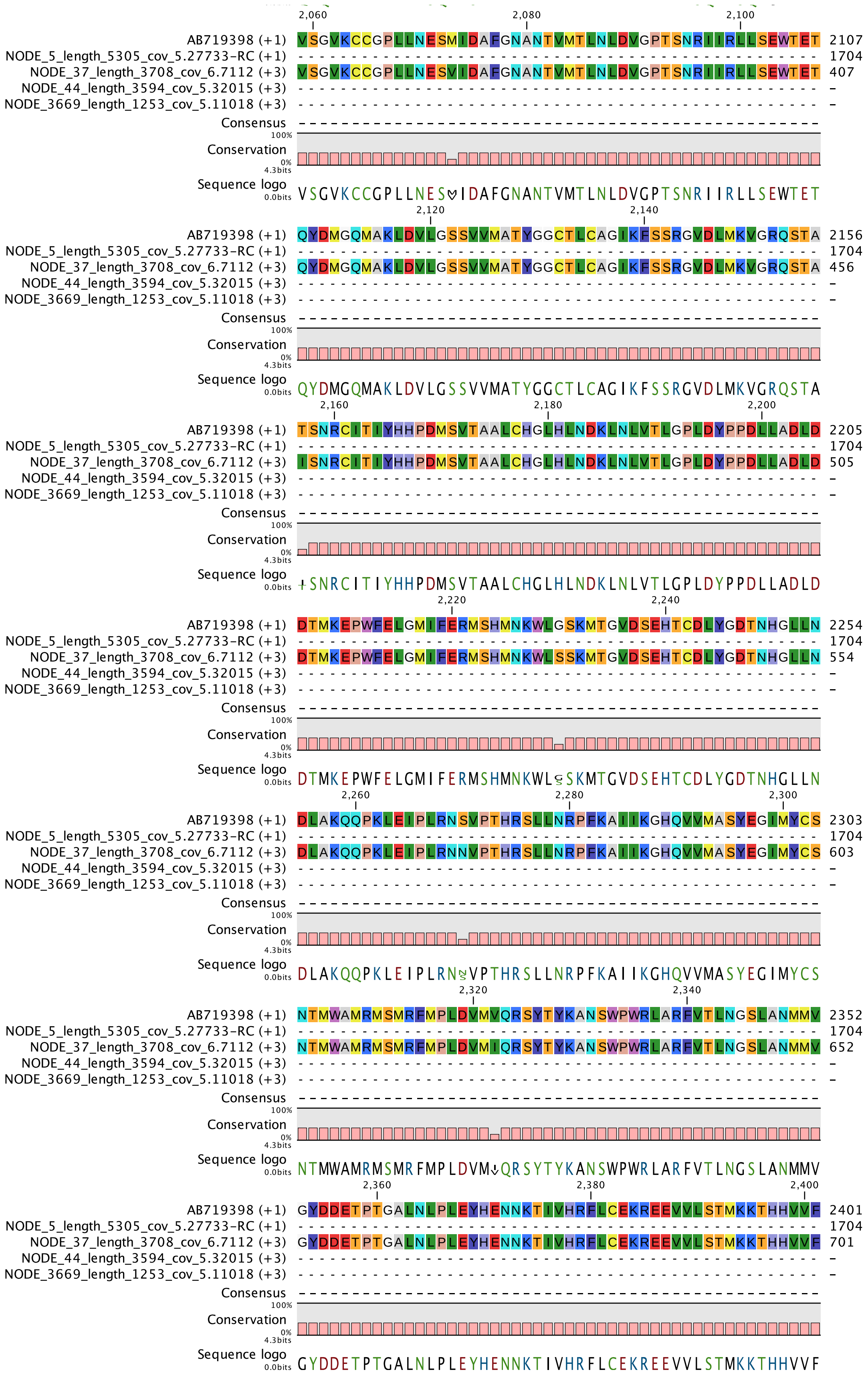
AB719398 (+1) VSGVKCCGPLLNESMIDAFGNANTVMTLNLDVGPTSNRIIRLLSEWTET 2107
NODE_5_length_5305_cov_5.27733-RC (+1) 1704
NODE_37_length_3708_cov_6.7112 (+3) VSGVKCCGPLLNESVIDAFGNANTVMTLNLDVGPTSNRIIRLLSEWTET 407
NODE_44_length_3594_cov_5.32015 (+3)
NODE_3669_length_1253_cov_5.11018 (+3)
Consensus
Conservation
Sequence logo
AB719398 (+1) QYDMGQMAKLDVLGSSVVMATYGGCTLCAGIKFSSRGVDLMKVGRQSTA 2156
NODE_5_length_5305_cov_5.27733-RC (+1) 1704
NODE_37_length_3708_cov_6.7112 (+3) QYDMGQMAKLDVLGSSVVMATYGGCTLCAGIKFSSRGVDLMKVGRQSTA 456
NODE_44_length_3594_cov_5.32015 (+3)
NODE_3669_length_1253_cov_5.11018 (+3)
Consensus
Conservation
Sequence logo
AB719398 (+1) TSNRCITIYHHPDMSVTAALCHGLHLNDKLNLVTLGPLDYPPDLLADLD 2205
NODE_5_length_5305_cov_5.27733-RC (+1) 1704
NODE_37_length_3708_cov_6.7112 (+3) ISNRCITIYHHPDMSVTAALCHGLHLNDKLNLVTLGPLDYPPDLLADLD 505
NODE_44_length_3594_cov_5.32015 (+3)
NODE_3669_length_1253_cov_5.11018 (+3)
Consensus
Conservation
Sequence logo
AB719398 (+1) DTMKEPWFELGMIFERMSHMNKWLGSKMTGVDSEHTCDLYGDTNHGLLN 2254
NODE_5_length_5305_cov_5.27733-RC (+1) 1704
NODE_37_length_3708_cov_6.7112 (+3) DTMKEPWFELGMIFERMSHMNKWLSSKMTGVDSEHTCDLYGDTNHGLLN 554
NODE_44_length_3594_cov_5.32015 (+3)
NODE_3669_length_1253_cov_5.11018 (+3)
Consensus
Conservation
Sequence logo
AB719398 (+1) DLAKQQPKLEIPLRNSVPTHRSLLNRPFKAIIKGHQVVMASYEGIMYCS 2303
NODE_5_length_5305_cov_5.27733-RC (+1) 1704
NODE_37_length_3708_cov_6.7112 (+3) DLAKQQPKLEIPLRNNVPTHRSLLNRPFKAIIKGHQVVMASYEGIMYCS 603
NODE_44_length_3594_cov_5.32015 (+3)
NODE_3669_length_1253_cov_5.11018 (+3)
Consensus
Conservation
Sequence logo
AB719398 (+1) NTMWAMRMSMRFMPLDVMVQRSYTYKANSWPWRLARFVTLNGSLANMMV 2352
NODE_5_length_5305_cov_5.27733-RC (+1) 1704
NODE_37_length_3708_cov_6.7112 (+3) NTMWAMRMSMRFMPLDVMIQRSYTYKANSWPWRLARFVTLNGSLANMMV 652
NODE_44_length_3594_cov_5.32015 (+3)
NODE_3669_length_1253_cov_5.11018 (+3)
Consensus
Conservation
Sequence logo
AB719398 (+1) GYDDETPTGALNLPLEYHENNKTIVHRFLCEKREEVVLSTMKKTHHVVF 2401
NODE_5_length_5305_cov_5.27733-RC (+1) 1704
NODE_37_length_3708_cov_6.7112 (+3) GYDDETPTGALNLPLEYHENNKTIVHRFLCEKREEVVLSTMKKTHHVVF 701
NODE_44_length_3594_cov_5.32015 (+3)
NODE_3669_length_1253_cov_5.11018 (+3)
Consensus
Conservation
Sequence logo

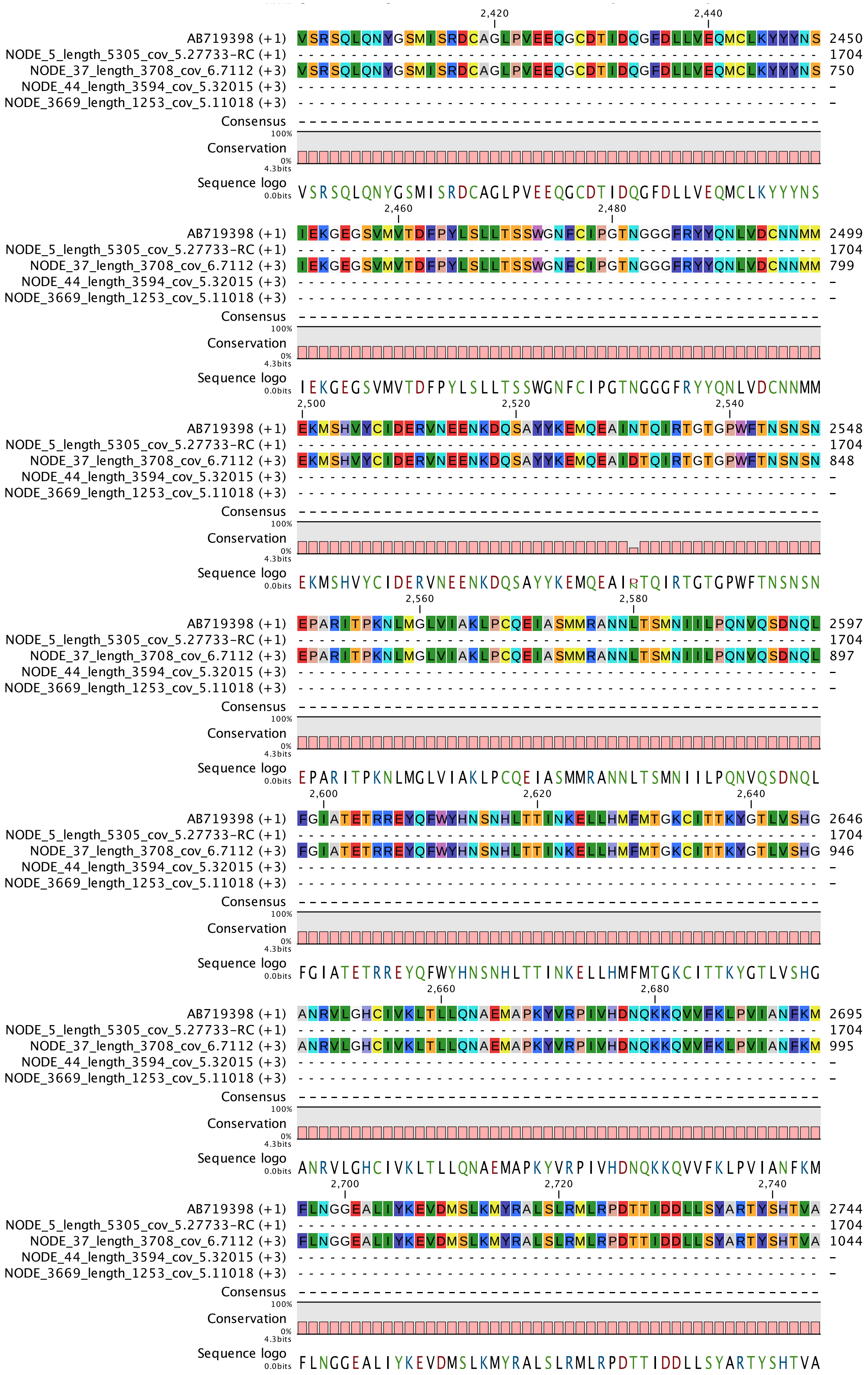
2,420 2,440
AB719398 (+1) VSRSQLQNYGSMISRDCAGLPVEEQGCDTIDQGFDLLVEQMCLKYYYNS 2450
NODE_5_length_5305_cov_5.27733-RC (+1) 1704
NODE_37_length_3708_cov_6.7112 (+3) VSRSQLQNYGSMISRDCAGLPVEEQGCDTIDQGFDLLVEQMCLKYYYNS 750
NODE_44_length_3594_cov_5.32015 (+3) –
NODE_3669_length_1253_cov_5.11018 (+3) –
Consensus
Conservation 100% 0%
Sequence logo 4.3bits 0.0bits VSRSQLQNYGSMISRDCAGLPVEEQGCDTIDQGFDLLVEQMCLKYYYNS
2,460 2,480
AB719398 (+1) IEKGEGSVMVTDFPYLSLLTSSWGNFCIPGTNGGGFRYYQNLVDCNNMM 2499
NODE_5_length_5305_cov_5.27733-RC (+1) 1704
NODE_37_length_3708_cov_6.7112 (+3) IEKGEGSVMVTDFPYLSLLTSSWGNFCIPGTNGGGFRYYQNLVDCNNMM 799
NODE_44_length_3594_cov_5.32015 (+3) –
NODE_3669_length_1253_cov_5.11018 (+3) –
Consensus
Conservation 100% 0%
Sequence logo 4.3bits 0.0bits IEKGEGSVMVTDFPYLSLLTSSWGNFCIPGTNGGGFRYYQNLVDCNNMM
2,500 2,520 2,540
AB719398 (+1) EKMSHVYCIDERVNEENKDQSAYYKEMQEAINTQIRTGTGPWFTNSNSN 2548
NODE_5_length_5305_cov_5.27733-RC (+1) 1704
NODE_37_length_3708_cov_6.7112 (+3) EKMSHVYCIDERVNEENKDQSAYYKEMQEAIDTQIRTGTGPWFTNSNSN 848
NODE_44_length_3594_cov_5.32015 (+3) –
NODE_3669_length_1253_cov_5.11018 (+3) –
Consensus
Conservation 100% 0%
Sequence logo 4.3bits 0.0bits EKMSHVYCIDERVNEENKDQSAYYKEMQEAI TQIRTGTGPWFTNSNSN
2,560 2,580
AB719398 (+1) EPARITPKNLMGLVIAKLPCQEIASMMRANNLTSMNIILPQNVQSDNQL 2597
NODE_5_length_5305_cov_5.27733-RC (+1) 1704
NODE_37_length_3708_cov_6.7112 (+3) EPARITPKNLMGLVIAKLPCQEIASMMRANNLTSMNIILPQNVQSDNQL 897
NODE_44_length_3594_cov_5.32015 (+3) –
NODE_3669_length_1253_cov_5.11018 (+3) –
Consensus
Conservation 100% 0%
Sequence logo 4.3bits 0.0bits EPARITPKNLMGLVIAKLPCQEIASMMRANNLTSMNIILPQNVQSDNQL
2,600 2,620 2,640
AB719398 (+1) FGIATETRREYQFWYHNSNHLTTINKELLHMFMTGKCITTKYGTLVSHG 2646
NODE_5_length_5305_cov_5.27733-RC (+1) 1704
NODE_37_length_3708_cov_6.7112 (+3) FGIATETRREYQFWYHNSNHLTTINKELLHMFMTGKCITTKYGTLVSHG 946
NODE_44_length_3594_cov_5.32015 (+3) –
NODE_3669_length_1253_cov_5.11018 (+3) –
Consensus
Conservation 100% 0%
Sequence logo 4.3bits 0.0bits FGIATETRREYQFWYHNSNHLTTINKELLHMFMTGKCITTKYGTLVSHG
2,660 2,680
AB719398 (+1) ANRVLGHCIVKLTLLQNAEMAPKYVRPIVHDNQKKQVVFKLPVIANFKM 2695
NODE_5_length_5305_cov_5.27733-RC (+1) 1704
NODE_37_length_3708_cov_6.7112 (+3) ANRVLGHCIVKLTLLQNAEMAPKYVRPIVHDNQKKQVVFKLPVIANFKM 995
NODE_44_length_3594_cov_5.32015 (+3) –
NODE_3669_length_1253_cov_5.11018 (+3) –
Consensus
Conservation 100% 0%
Sequence logo 4.3bits 0.0bits ANRVLGHCIVKLTLLQNAEMAPKYVRPIVHDNQKKQVVFKLPVIANFKM
2,700 2,720 2,740
AB719398 (+1) FLNGGEALIYKEVDMSLKMYRALSLRMLRPDTTIDDLLSYARTYSHTVA 2744
NODE_5_length_5305_cov_5.27733-RC (+1) 1704
NODE_37_length_3708_cov_6.7112 (+3) FLNGGEALIYKEVDMSLKMYRALSLRMLRPDTTIDDLLSYARTYSHTVA 1044
NODE_44_length_3594_cov_5.32015 (+3) –
NODE_3669_length_1253_cov_5.11018 (+3) –
Consensus
Conservation 100% 0%
Sequence logo 4.3bits 0.0bits FLNGGEALIYKEVDMSLKMYRALSLRMLRPDTTIDDLLSYARTYSHTVA

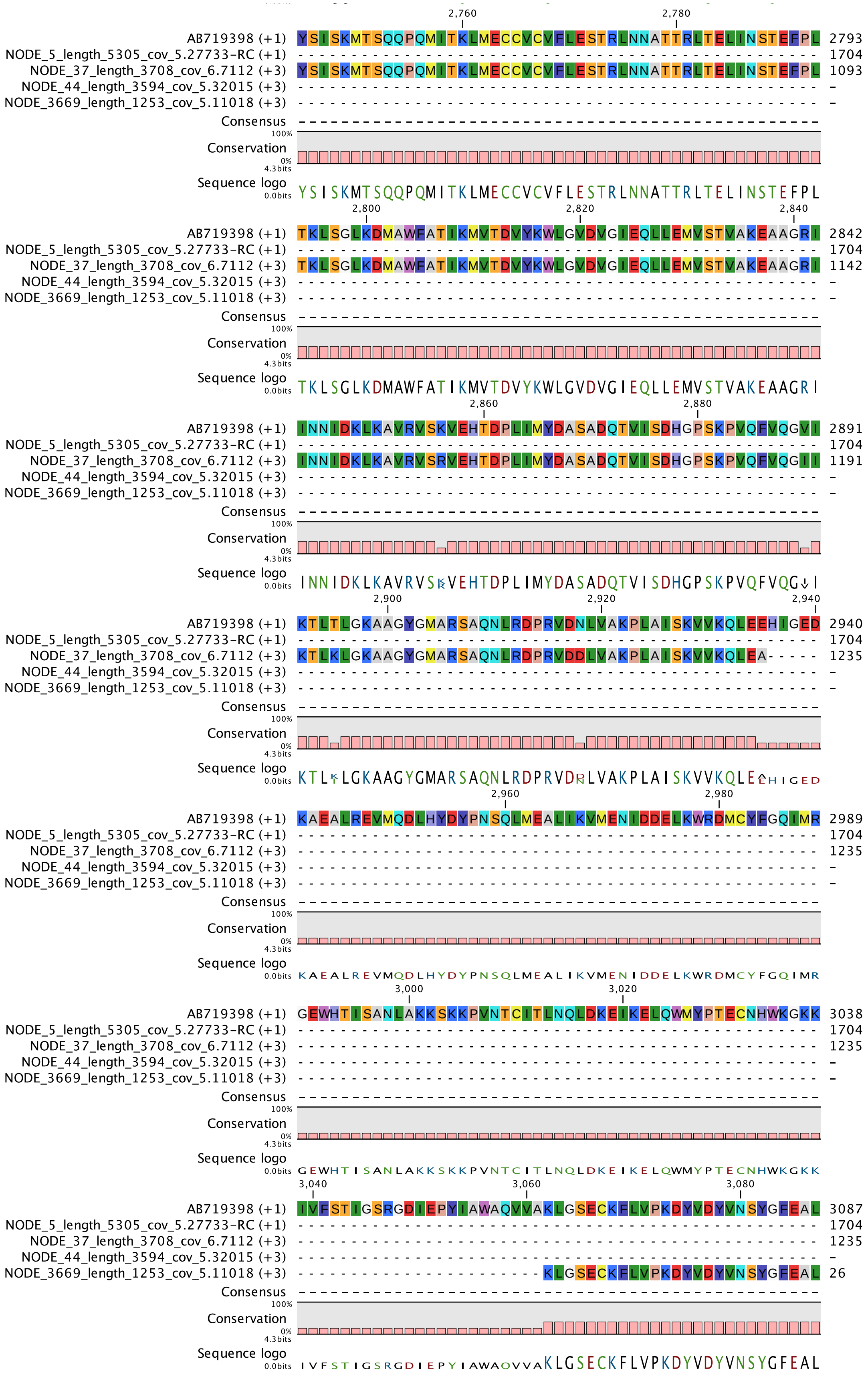
AB719398 (+1) YSISKMTSQQPQMITKLMECCVCVFLESTRLNNATTRLTELINSTEFPL 2793
NODE_5_length_5305_cov_5.27733-RC (+1) 1704
NODE_37_length_3708_cov_6.7112 (+3) YSISKMTSQQPQMITKLMECCVCVFLESTRLNNATTRLTELINSTEFPL 1093
NODE_44_length_3594_cov_5.32015 (+3)
NODE_3669_length_1253_cov_5.11018 (+3)
Consensus
Conservation
Sequence logo
AB719398 (+1) TKLSGLKDMAWFATIKMVTDVYKWLGVDVGIEQLLEMVSTVAKEAAGRI 2842
NODE_37_length_3708_cov_6.7112 (+3) TKLSGLKDMAWFATIKMVTDVYKWLGVDVGIEQLLEMVSTVAKEAAGRI 1142
AB719398 (+1) INNIDKLKAVRVSKVEHTDPLIMYDASADQTVISDHGPSKPVQFVQGVI 2891
NODE_37_length_3708_cov_6.7112 (+3) INNIDKLKAVRVSRVEHTDPLIMYDASADQTVISDHGPSKPVQFVQGII 1191
AB719398 (+1) KTLTLGKAAGYGMARSAQNLRDPRVDNLVAKPLAISKVVKQLEEHIGED 2940
NODE_37_length_3708_cov_6.7112 (+3) KTLKLGKAAGYGMARSAQNLRDPRVDDLVAKPLAISKVVKQLEA 1235
AB719398 (+1) KAEALREVMQDLHYDYPNSQLMEALIKVMENIDDELKWRDMCYFGQIMR 2989
AB719398 (+1) GEWHTISANLAKKSKKPVNTCITLNQLDKEIKELQWMYPTECNHWKGKK 3038
AB719398 (+1) IVFSTIGSRGDIEPYIAWAQVVAKLGSECKFLVPKDYVDYVNSYGFEAL 3087
NODE_3669_length_1253_cov_5.11018 (+3) KLGSECKFLVPKDYVDYVNSYGFEAL 26

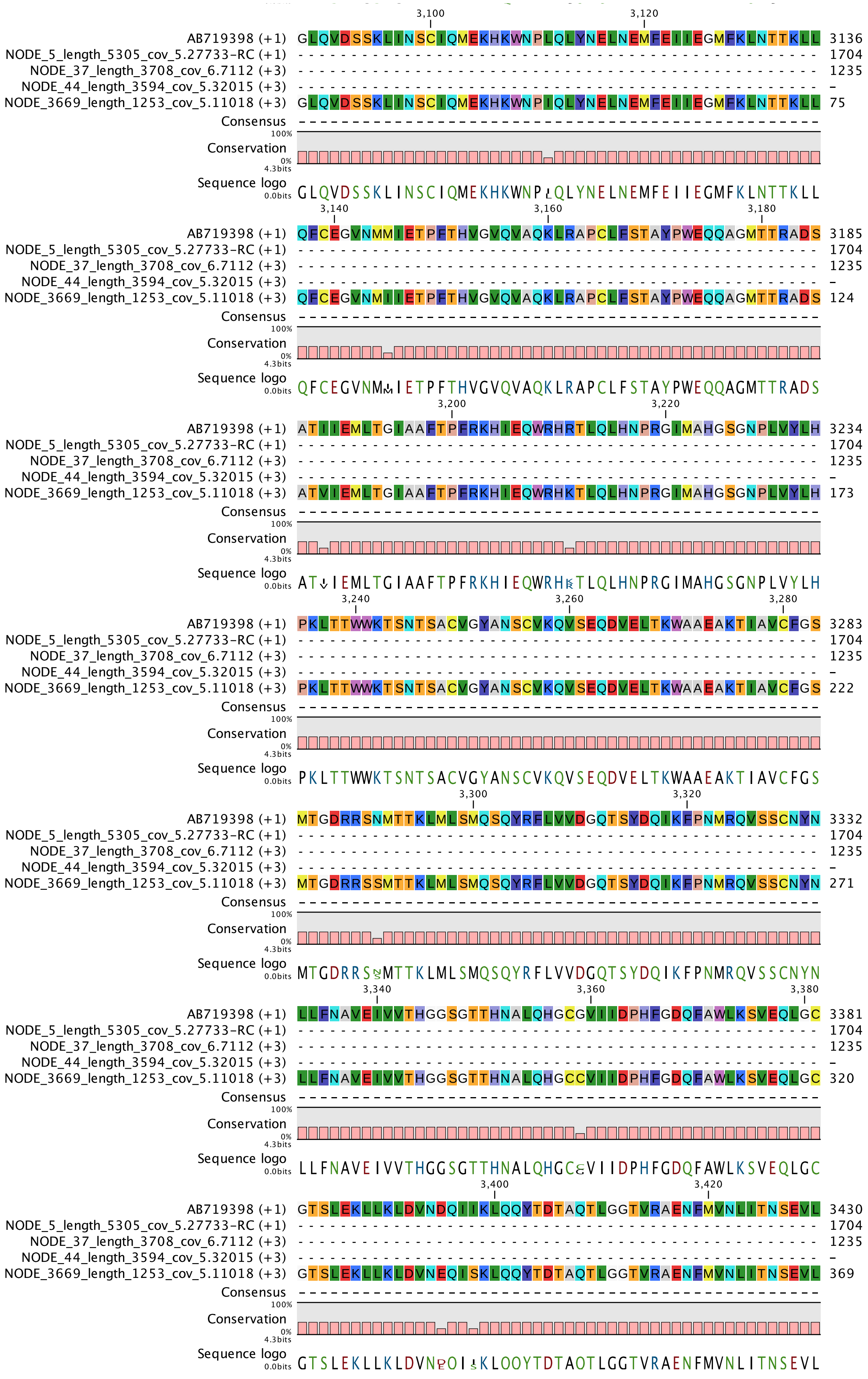

3,100 3,120
AB719398 (+1) GLQVDSSKLINSCIQMEKHKWNPLQLYNELNEMFEIIEGMFKLNTTKLL 3136
NODE_5_length_5305_cov_5.27733-RC (+1) 1704
NODE_37_length_3708_cov_6.7112 (+3) 1235
NODE_44_length_3594_cov_5.32015 (+3) –
NODE_3669_length_1253_cov_5.11018 (+3) GLQVDSSKLINSCIQMEKHKWNPIQLYNELNEMFEIIEGMFKLNTTKLL 75
Consensus
Conservation
Sequence logo
3,140 3,160 3,180
AB719398 (+1) QFCEGVNMMIETPFTHVGVQVAQKLRAPCLFSTAYPWEQQAGMTTRADS 3185
NODE_5_length_5305_cov_5.27733-RC (+1) 1704
NODE_37_length_3708_cov_6.7112 (+3) 1235
NODE_44_length_3594_cov_5.32015 (+3) –
NODE_3669_length_1253_cov_5.11018 (+3) QFCEGVNMIIETPFTHVGVQVAQKLRAPCLFSTAYPWEQQAGMTTRADS 124
Consensus
Conservation
Sequence logo
3,200 3,220
AB719398 (+1) ATIIEMLTGIAAFTPFRKHIEQWRHRTLQLHNPRGIMAHGSGNPLVYLH 3234
NODE_5_length_5305_cov_5.27733-RC (+1) 1704
NODE_37_length_3708_cov_6.7112 (+3) 1235
NODE_44_length_3594_cov_5.32015 (+3) –
NODE_3669_length_1253_cov_5.11018 (+3) ATVIEMLTGIAAFTPFRKHIEQWRHKTLQLHNPRGIMAHGSGNPLVYLH 173
Consensus
Conservation
Sequence logo
3,240 3,260 3,280
AB719398 (+1) PKLTTWWKTSNTSACVGYANSCVKQVSEQDVELTKWAAEAKTIAVCFGS 3283
NODE_5_length_5305_cov_5.27733-RC (+1) 1704
NODE_37_length_3708_cov_6.7112 (+3) 1235
NODE_44_length_3594_cov_5.32015 (+3) –
NODE_3669_length_1253_cov_5.11018 (+3) PKLTTWWKTSNTSACVGYANSCVKQVSEQDVELTKWAAEAKTIAVCFGS 222
Consensus
Conservation
Sequence logo
3,300 3,320
AB719398 (+1) MTGDRRSNMTTKLMLSMQSQYRFLVVDGQTSYDQIKFPNMRQVSSCNYN 3332
NODE_5_length_5305_cov_5.27733-RC (+1) 1704
NODE_37_length_3708_cov_6.7112 (+3) 1235
NODE_44_length_3594_cov_5.32015 (+3) –
NODE_3669_length_1253_cov_5.11018 (+3) MTGDRRSSMTTKLMLSMQSQYRFLVVDGQTSYDQIKFPNMRQVSSCNYN 271
Consensus
Conservation
Sequence logo
3,340 3,360 3,380
AB719398 (+1) LLFNAVEIVVTHGGSGTTHNALQHGCGVIIDPHFGDQFAWLKSVEQLGC 3381
NODE_5_length_5305_cov_5.27733-RC (+1) 1704
NODE_37_length_3708_cov_6.7112 (+3) 1235
NODE_44_length_3594_cov_5.32015 (+3) –
NODE_3669_length_1253_cov_5.11018 (+3) LLFNAVEIVVTHGGSGTTHNALQHGCCVIIDPHFGDQFAWLKSVEQLGC 320
Consensus
Conservation
Sequence logo
3,400 3,420
AB719398 (+1) GTSLEKLLKLDVNDQIIKLQQYTDTAQTLGGTVRAENFMVNLITNSEVL 3430
NODE_5_length_5305_cov_5.27733-RC (+1) 1704
NODE_37_length_3708_cov_6.7112 (+3) 1235
NODE_44_length_3594_cov_5.32015 (+3) –
NODE_3669_length_1253_cov_5.11018 (+3) GTSLEKLLKLDVNEQISKLQQYTDTAQTLGGTVRAENFMVNLITNSEVL 369
Consensus
Conservation
Sequence logo

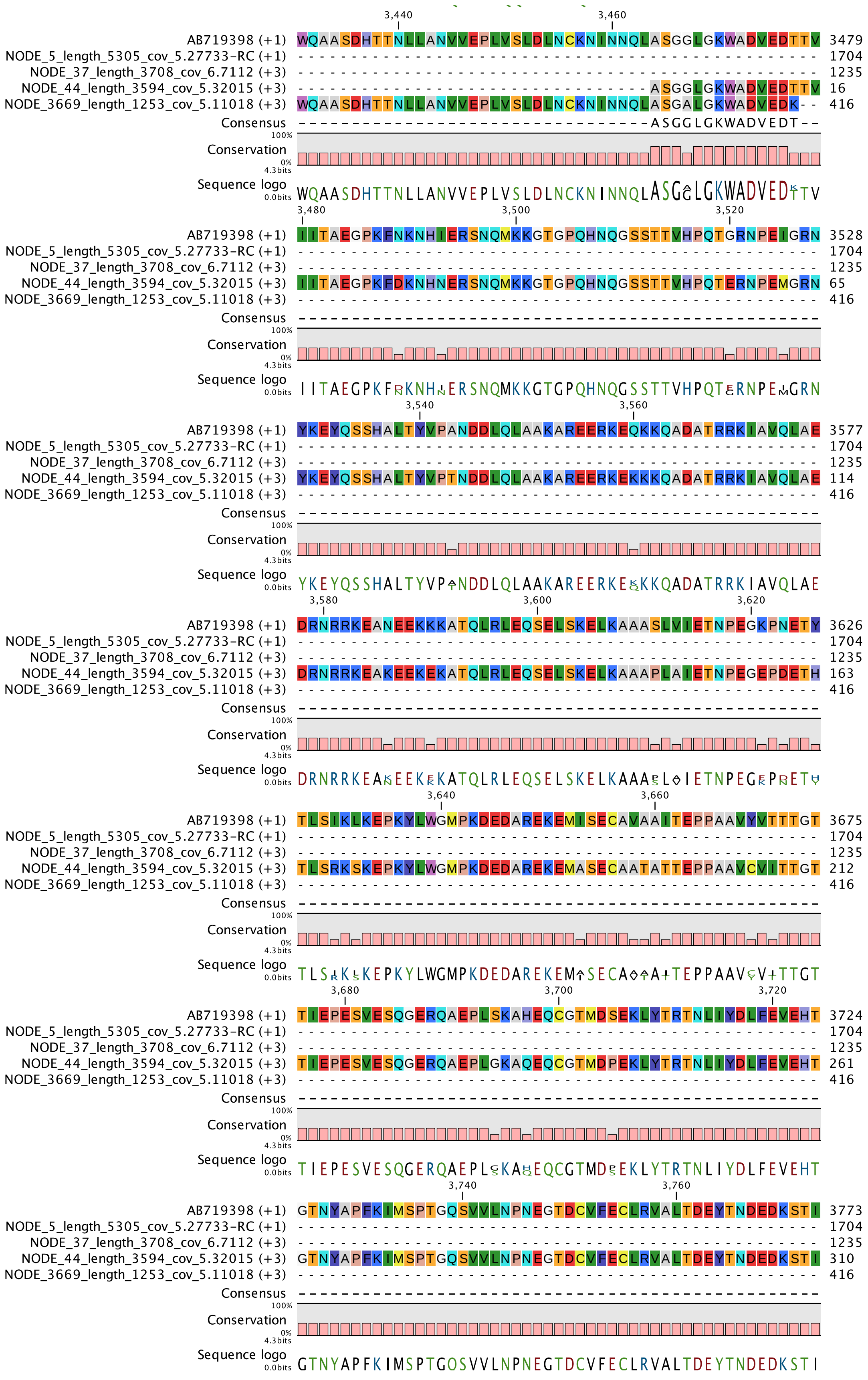

3,440 3,460
AB719398 (+1) WQAASDHTTNLLANVVEPLVSLDLNCKNINNQLASGGLGKWADVEDTTV 3479
NODE_5_length_5305_cov_5.27733-RC (+1) --- 1704
NODE_37_length_3708_cov_6.7112 (+3) --- 1235
NODE_44_length_3594_cov_5.32015 (+3) ---------------------------------ASGGLGKWADVEDTTV 16
NODE_3669_length_1253_cov_5.11018 (+3) WQAASDHTTNLLANVVEPLVSLDLNCKNINNQLASGALGKWADVEDK-- 416
Consensus ---------------------------------ASGGLGKWADVEDT--
Conservation
Sequence logo
3,480 3,500 3,520
AB719398 (+1) IITAEGPKFNKNHIERSNQMKKGTGPQHNQGSSTTVHPQTGRNPEIGRN 3528
NODE_5_length_5305_cov_5.27733-RC (+1) --- 1704
NODE_37_length_3708_cov_6.7112 (+3) --- 1235
NODE_44_length_3594_cov_5.32015 (+3) IITAEGPKFDKNHNERSNQMKKGTGPQHNQGSSTTVHPQTERNPEMGRN 65
NODE_3669_length_1253_cov_5.11018 (+3) --- 416
Consensus ---
Conservation
Sequence logo
3,540 3,560
AB719398 (+1) YKEYQSSHALTYVPANDDLQLAAKAREERKEQKKQADATRRKIAVQLAE 3577
NODE_5_length_5305_cov_5.27733-RC (+1) --- 1704
NODE_37_length_3708_cov_6.7112 (+3) --- 1235
NODE_44_length_3594_cov_5.32015 (+3) YKEYQSSHALTYVPTNDDLQLAAKAREERKEKKKQADATRRKIAVQLAE 114
NODE_3669_length_1253_cov_5.11018 (+3) --- 416
Consensus ---
Conservation
Sequence logo
3,580 3,600 3,620
AB719398 (+1) DRNRRKEANEEKKKATQLRLEQSELSKELKAAASLVIETNPEGKPNETY 3626
NODE_5_length_5305_cov_5.27733-RC (+1) --- 1704
NODE_37_length_3708_cov_6.7112 (+3) --- 1235
NODE_44_length_3594_cov_5.32015 (+3) DRNRRKEAKEEKEKATQLRLEQSELSKELKAAAPLAIETNPEGEPDETH 163
NODE_3669_length_1253_cov_5.11018 (+3) --- 416
Consensus ---
Conservation
Sequence logo
3,640 3,660
AB719398 (+1) TLSIKLKEPKYLWGMPKDEDAREKEMISECAVAAITEPPAAVYVTTTGT 3675
NODE_5_length_5305_cov_5.27733-RC (+1) --- 1704
NODE_37_length_3708_cov_6.7112 (+3) --- 1235
NODE_44_length_3594_cov_5.32015 (+3) TLSRKSKEPKYLWGMPKDEDAREKEMASECAATATTEPPAAVCVITTGT 212
NODE_3669_length_1253_cov_5.11018 (+3) --- 416
Consensus ---
Conservation
Sequence logo
3,680 3,700 3,720
AB719398 (+1) TIEPESVESQGERQAEPLSKAHEQCGTMDSEKLYTRTNLIYDLFEVEHT 3724
NODE_5_length_5305_cov_5.27733-RC (+1) --- 1704
NODE_37_length_3708_cov_6.7112 (+3) --- 1235
NODE_44_length_3594_cov_5.32015 (+3) TIEPESVESQGERQAEPLGKAQEQCGTMDPEKLYTRTNLIYDLFEVEHT 261
NODE_3669_length_1253_cov_5.11018 (+3) --- 416
Consensus ---
Conservation
Sequence logo
3,740 3,760
AB719398 (+1) GTNYAPFKIMSPTGQSVVLNPNEGTDCVFECLRVALTDEYTNDEDKSTI 3773
NODE_5_length_5305_cov_5.27733-RC (+1) --- 1704
NODE_37_length_3708_cov_6.7112 (+3) --- 1235
NODE_44_length_3594_cov_5.32015 (+3) GTNYAPFKIMSPTGQSVVLNPNEGTDCVFECLRVALTDEYTNDEDKSTI 310
NODE_3669_length_1253_cov_5.11018 (+3) --- 416
Consensus ---
Conservation
Sequence logo

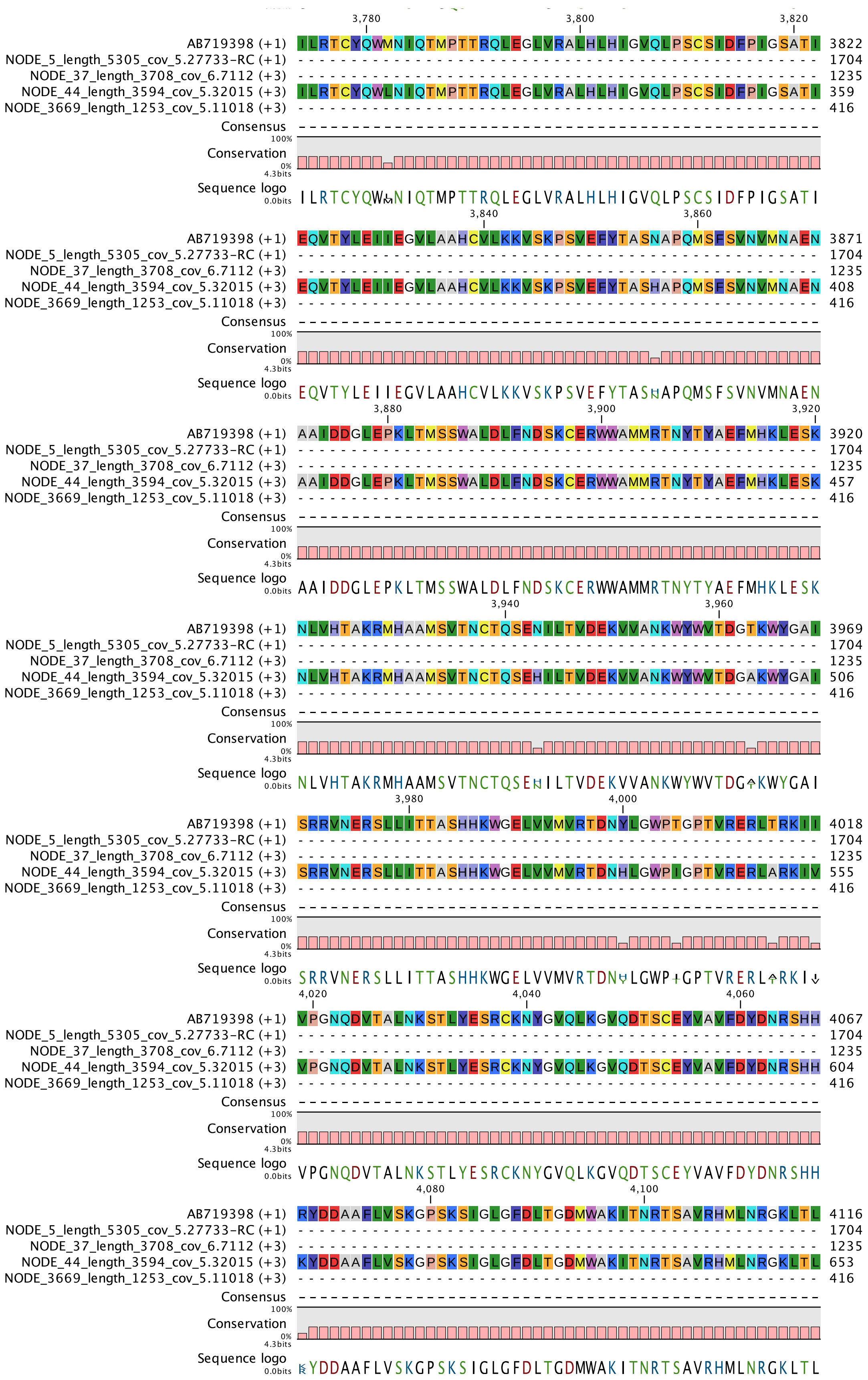

3,780 3,800 3,820
AB719398 (+1) ILRTCYQWMNIQTMPTTRQLEGLVRALHLHIGVQLPSCSIDFPIGSATI 3822
NODE_5_length_5305_cov_5.27733-RC (+1) 1704
NODE_37_length_3708_cov_6.7112 (+3) 1235
NODE_44_length_3594_cov_5.32015 (+3) ILRTCYQWLNIQTMPTTRQLEGLVRALHLHIGVQLPSCSIDFPIGSATI 359
NODE_3669_length_1253_cov_5.11018 (+3) 416
Consensus
Conservation
Sequence logo
3,840 3,860
AB719398 (+1) EQVTYLEIIEGVLAAHCVLKKVSKPSVEFYTASNAPQMSFSVNVMNAEN 3871
NODE_5_length_5305_cov_5.27733-RC (+1) 1704
NODE_37_length_3708_cov_6.7112 (+3) 1235
NODE_44_length_3594_cov_5.32015 (+3) EQVTYLEIIEGVLAAHCVLKKVSKPSVEFYTASHAPQMSFSVNVMNAEN 408
NODE_3669_length_1253_cov_5.11018 (+3) 416
Consensus
Conservation
Sequence logo
3,880 3,900 3,920
AB719398 (+1) AAIDDGLEPKLTMSSWALDLFNDSKCERWWAMMRTNYTYAEFMHKLESK 3920
NODE_5_length_5305_cov_5.27733-RC (+1) 1704
NODE_37_length_3708_cov_6.7112 (+3) 1235
NODE_44_length_3594_cov_5.32015 (+3) AAIDDGLEPKLTMSSWALDLFNDSKCERWWAMMRTNYTYAEFMHKLESK 457
NODE_3669_length_1253_cov_5.11018 (+3) 416
Consensus
Conservation
Sequence logo
3,940 3,960
AB719398 (+1) NLVHTAKRMHAAMSVTNCTQSENILTVDEKVVANKWYWVTDGTKWYGAI 3969
NODE_5_length_5305_cov_5.27733-RC (+1) 1704
NODE_37_length_3708_cov_6.7112 (+3) 1235
NODE_44_length_3594_cov_5.32015 (+3) NLVHTAKRMHAAMSVTNCTQSEHILTVDEKVVANKWYWVTDGAKWYGAI 506
NODE_3669_length_1253_cov_5.11018 (+3) 416
Consensus
Conservation
Sequence logo
3,980 4,000
AB719398 (+1) SRRVNERSLLITTASHHKWGELVVMVRTDNYLGWPTGPTVRERLTRKII 4018
NODE_5_length_5305_cov_5.27733-RC (+1) 1704
NODE_37_length_3708_cov_6.7112 (+3) 1235
NODE_44_length_3594_cov_5.32015 (+3) SRRVNERSLLITTASHHKWGELVVMVRTDNHLGWPIGPTVRERLARKIV 555
NODE_3669_length_1253_cov_5.11018 (+3) 416
Consensus
Conservation
Sequence logo
4,020 4,040 4,060
AB719398 (+1) VPGNQDVTALNKSTLYESRCKNYGVQLKGVQDTSCEYVAVFDYDNRSHH 4067
NODE_5_length_5305_cov_5.27733-RC (+1) 1704
NODE_37_length_3708_cov_6.7112 (+3) 1235
NODE_44_length_3594_cov_5.32015 (+3) VPGNQDVTALNKSTLYESRCKNYGVQLKGVQDTSCEYVAVFDYDNRSHH 604
NODE_3669_length_1253_cov_5.11018 (+3) 416
Consensus
Conservation
Sequence logo
4,080 4,100
AB719398 (+1) RYDDAAFLVSKGPSKSIGLGFDLTGDMWAKITNRTSAVRHMLNRGKLTL 4116
NODE_5_length_5305_cov_5.27733-RC (+1) 1704
NODE_37_length_3708_cov_6.7112 (+3) 1235
NODE_44_length_3594_cov_5.32015 (+3) KYDDAAFLVSKGPSKSIGLGFDLTGDMWAKITNRTSAVRHMLNRGKLTL 653
NODE_3669_length_1253_cov_5.11018 (+3) 416
Consensus
Conservation
Sequence logo

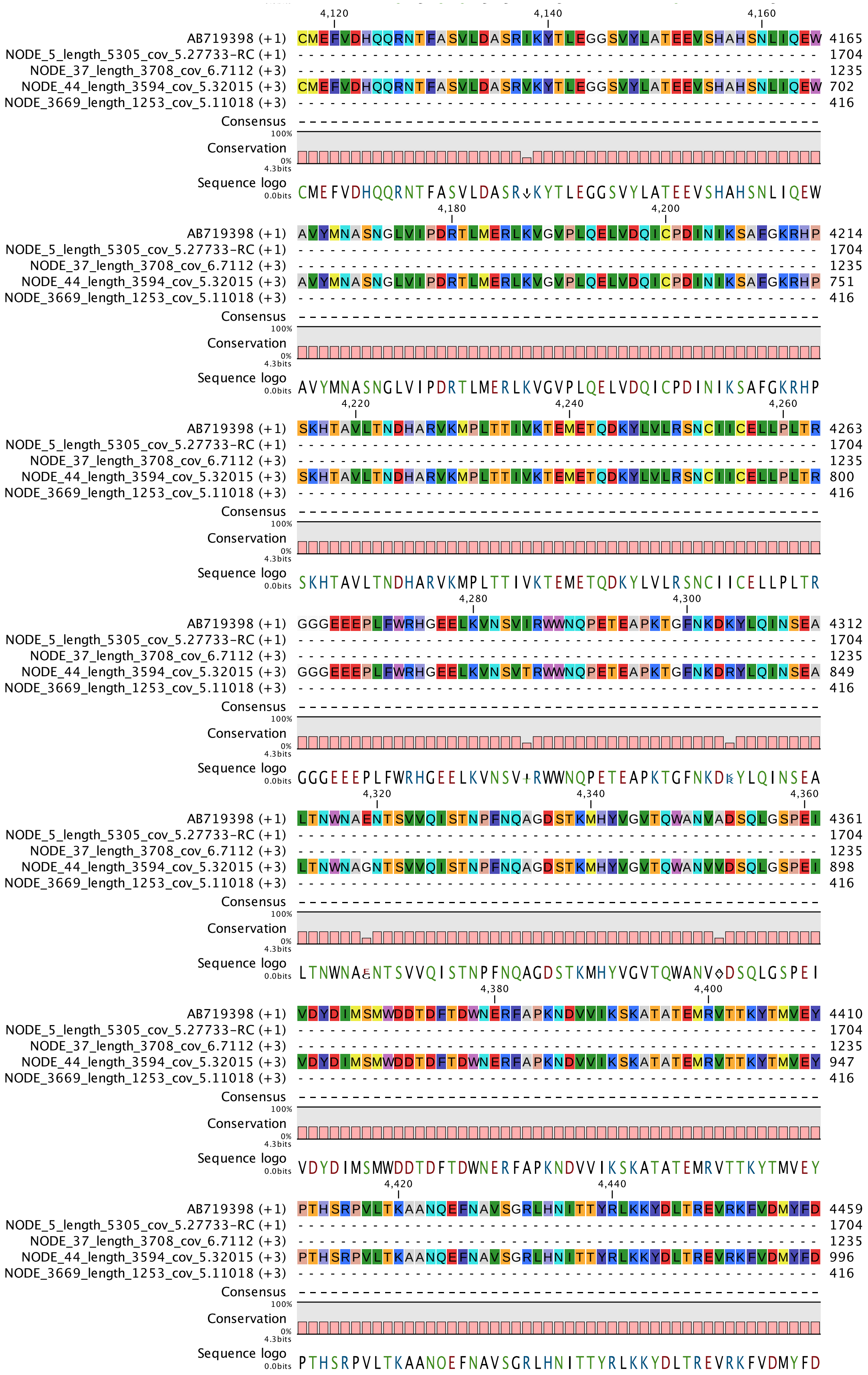
AB719398 (+1) CMEFVDHQQRNTFASVLDASRIKYTLEGGSVYLATEEVSHAHSNLIQEW 4165
NODE_5_length_5305_cov_5.27733-RC (+1) 1704
NODE_37_length_3708_cov_6.7112 (+3) 1235
NODE_44_length_3594_cov_5.32015 (+3) CMEFVDHQQRNTFASVLDASRVKYTLEGGSVYLATEEVSHAHSNLIQEW 702
NODE_3669_length_1253_cov_5.11018 (+3) 416
AB719398 (+1) AVYMNASNGLVIPDRTLMERLKVGVPLQELVDQICPDINIKSAFGKRHP 4214
NODE_44_length_3594_cov_5.32015 (+3) AVYMNASNGLVIPDRTLMERLKVGVPLQELVDQICPDINIKSAFGKRHP 751
AB719398 (+1) SKHTAVLTNDHARVKMPLTTIVKTEMETQDKYLVLRSNCIICELLPLTR 4263
NODE_44_length_3594_cov_5.32015 (+3) SKHTAVLTNDHARVKMPLTTIVKTEMETQDKYLVLRSNCIICELLPLTR 800
AB719398 (+1) GGGEEEPLFWRHGEELKVNSVIRWWNQPETEAPKTGFNKDKYLQINSEA 4312
NODE_44_length_3594_cov_5.32015 (+3) GGGEEEPLFWRHGEELKVNSVTRWWNQPETEAPKTGFNKDRYLQINSEA 849
AB719398 (+1) LTNWNAENTSVVQISTNPFNQAGDSTKMHYVGVTQWANVADSQLGSPEI 4361
NODE_44_length_3594_cov_5.32015 (+3) LTNWNAGNTSVVQISTNPFNQAGDSTKMHYVGVTQWANVVDSQLGSPEI 898
AB719398 (+1) VDYDIMSMWDDTDFTDWNERFAPKNDVVIKSKATATEMRVTTKYTMVEY 4410
NODE_44_length_3594_cov_5.32015 (+3) VDYDIMSMWDDTDFTDWNERFAPKNDVVIKSKATATEMRVTTKYTMVEY 947
AB719398 (+1) PTHSRPVLTKAANQEFNAVSGRLHNITTYRLKKYDLTREVRKFVDMYFD 4459
NODE_44_length_3594_cov_5.32015 (+3) PTHSRPVLTKAANQEFNAVSGRLHNITTYRLKKYDLTREVRKFVDMYFD 996
Consensus
Conservation
Sequence logo

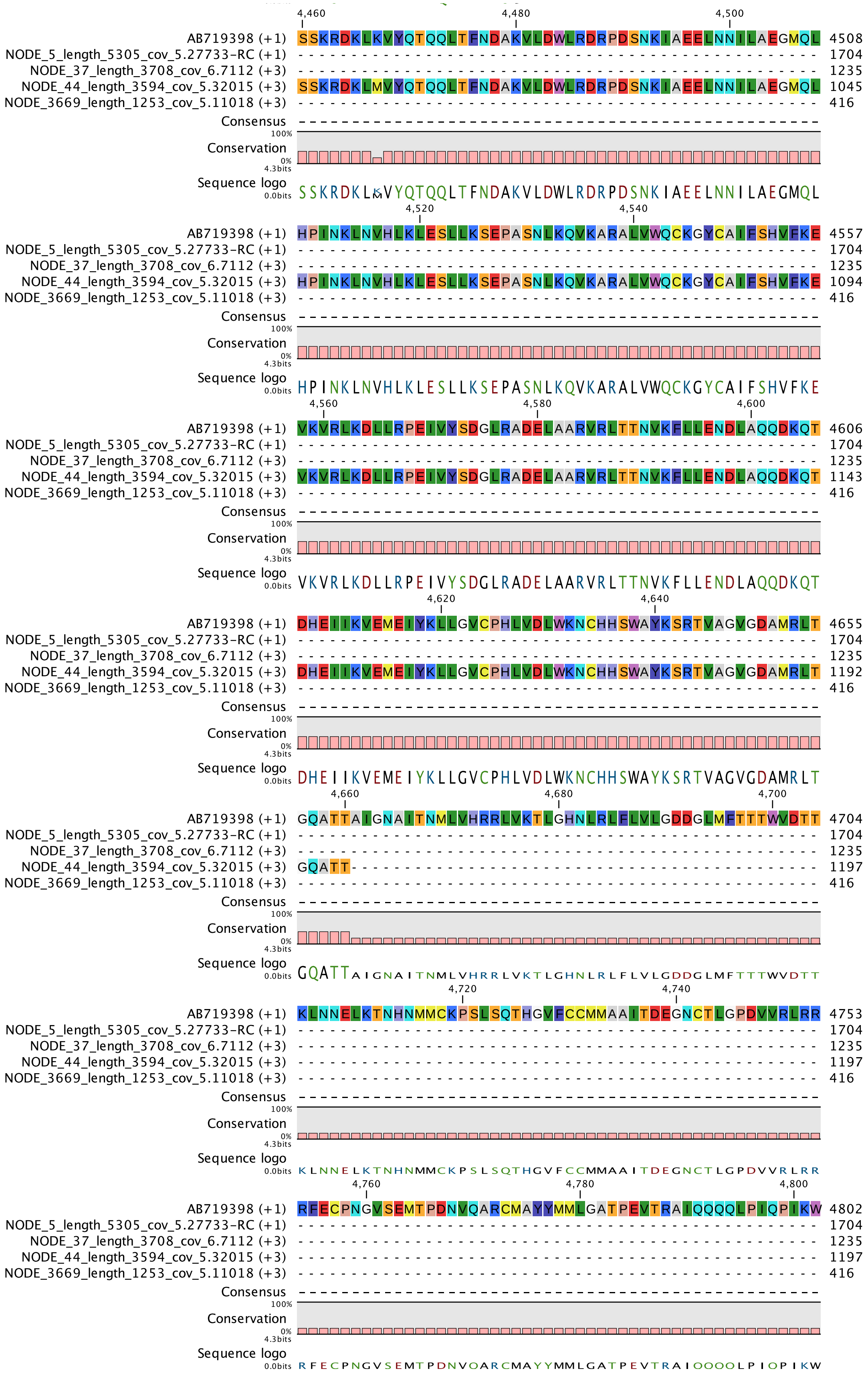
4,460 4,480 4,500
AB719398 (+1) SSKRDKLKVYQTQQLTFNDAKVLDWLRDRPDSNKIAEELNNILAEGMQL 4508
NODE_5_length_5305_cov_5.27733-RC (+1) 1704
NODE_37_length_3708_cov_6.7112 (+3) 1235
NODE_44_length_3594_cov_5.32015 (+3) SSKRDKLMVYQTQQLTFNDAKVLDWLRDRPDSNKIAEELNNILAEGMQL 1045
NODE_3669_length_1253_cov_5.11018 (+3) 416
Consensus
Conservation
Sequence logo
4,520 4,540
AB719398 (+1) HPINKLNVHLKLESLLKSEPASNLKQVKARALVWQCKGYCAIFSHVFKE 4557
NODE_5_length_5305_cov_5.27733-RC (+1) 1704
NODE_37_length_3708_cov_6.7112 (+3) 1235
NODE_44_length_3594_cov_5.32015 (+3) HPINKLNVHLKLESLLKSEPASNLKQVKARALVWQCKGYCAIFSHVFKE 1094
NODE_3669_length_1253_cov_5.11018 (+3) 416
Consensus
Conservation
Sequence logo
4,560 4,580 4,600
AB719398 (+1) VKVRLKDLLRPEIVYSDGLRADELAARVRLTTNVKFLLENDLAQQDKQT 4606
NODE_5_length_5305_cov_5.27733-RC (+1) 1704
NODE_37_length_3708_cov_6.7112 (+3) 1235
NODE_44_length_3594_cov_5.32015 (+3) VKVRLKDLLRPEIVYSDGLRADELAARVRLTTNVKFLLENDLAQQDKQT 1143
NODE_3669_length_1253_cov_5.11018 (+3) 416
Consensus
Conservation
Sequence logo
4,620 4,640
AB719398 (+1) DHEIIKVEMEIYKLLGVCPHLVDLWKNCHHSWAYKSRTVAGVGDAMRLT 4655
NODE_5_length_5305_cov_5.27733-RC (+1) 1704
NODE_37_length_3708_cov_6.7112 (+3) 1235
NODE_44_length_3594_cov_5.32015 (+3) DHEIIKVEMEIYKLLGVCPHLVDLWKNCHHSWAYKSRTVAGVGDAMRLT 1192
NODE_3669_length_1253_cov_5.11018 (+3) 416
Consensus
Conservation
Sequence logo
4,660 4,680 4,700
AB719398 (+1) GQATTAIGNAITNMLVHRRLVKTLGHNLRLFLVLGDDGLMFTTTWVDTT 4704
NODE_5_length_5305_cov_5.27733-RC (+1) 1704
NODE_37_length_3708_cov_6.7112 (+3) 1235
NODE_44_length_3594_cov_5.32015 (+3) GQATT 1197
NODE_3669_length_1253_cov_5.11018 (+3) 416
Consensus
Conservation
Sequence logo
4,720 4,740
AB719398 (+1) KLNNELKTNHNMMCKPSLSQTHGVFCCMMAAITDEGNCTLGPDVVRLRR 4753
NODE_5_length_5305_cov_5.27733-RC (+1) 1704
NODE_37_length_3708_cov_6.7112 (+3) 1235
NODE_44_length_3594_cov_5.32015 (+3) 1197
NODE_3669_length_1253_cov_5.11018 (+3) 416
Consensus
Conservation
Sequence logo
4,760 4,780 4,800
AB719398 (+1) RFECPNGVSEMTPDNVQARCMAYYMMLGATPEVTRAIQQQQLPIQPIKW 4802
NODE_5_length_5305_cov_5.27733-RC (+1) 1704
NODE_37_length_3708_cov_6.7112 (+3) 1235
NODE_44_length_3594_cov_5.32015 (+3) 1197
NODE_3669_length_1253_cov_5.11018 (+3) 416
Consensus
Conservation
Sequence logo

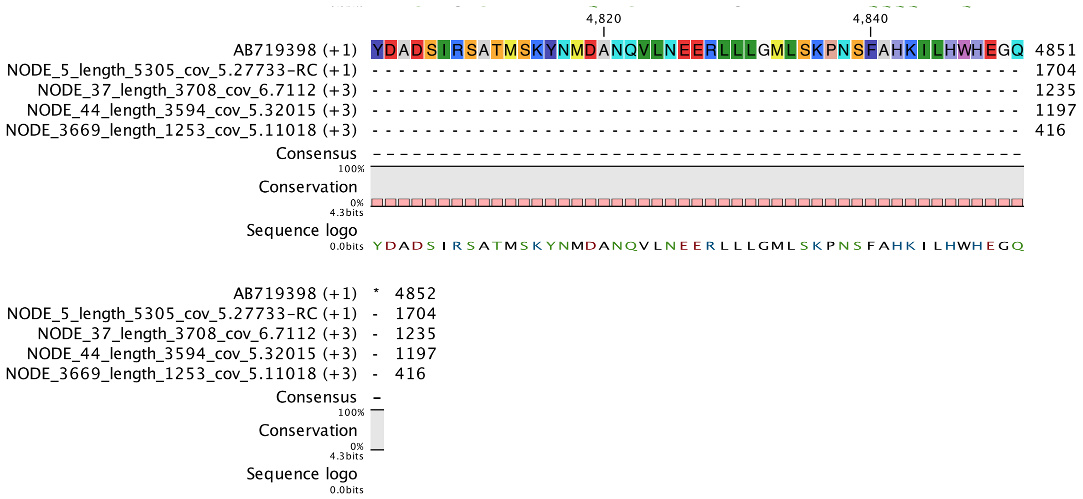

Supplemental Figure S3. Sequence alignment of the amino acids of the PvEV2 strain accession number AB719398 (Okada et al., 2013) and the various fragments of the Kenyan isolates of PvEV2. Sequences were aligned by the CLC Sequence Viewer 8.0 application.
