# Supplementary material for: Metagenomic Analysis of Plant Virus Occurrence in Common Bean (Phaseolus vulgaris) in Central Kenya
Source: Front Microbiol. 2018 Dec 7;9:2939. doi: 10.3389/fmicb.2018.02939 (PMC6293961; doi:10.3389/fmicb.2018.02939)
Supplement: Supplementary file 3 [file Data_Sheet_3.PDF]

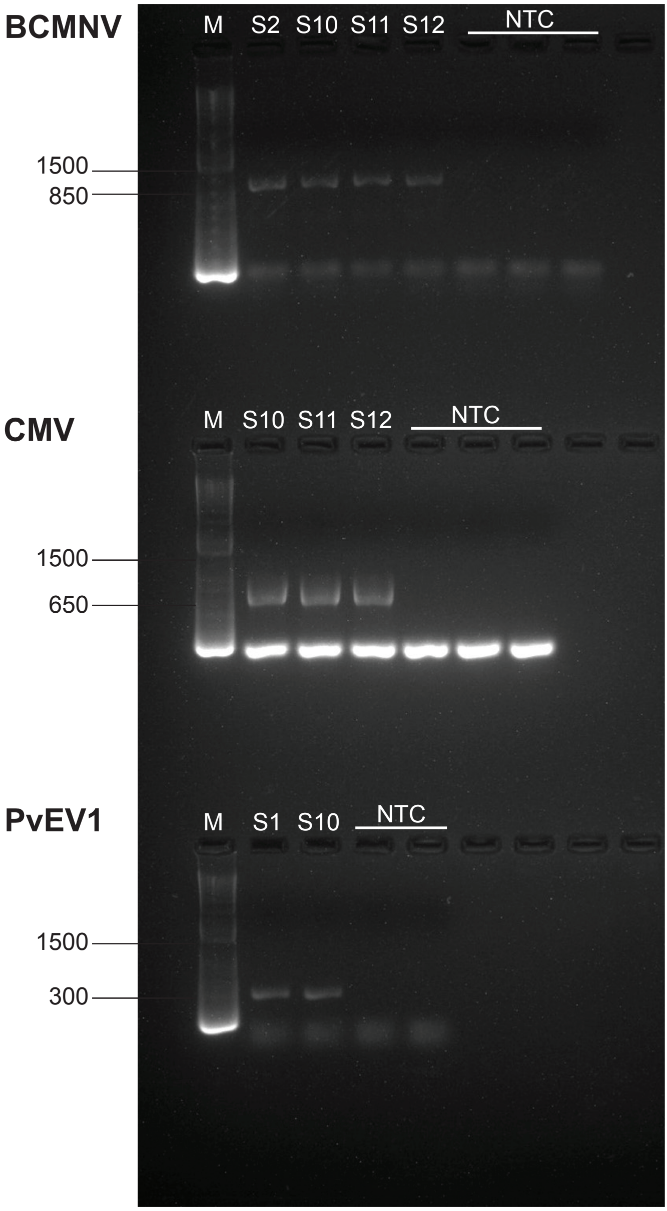

Supplemental Figure S4. Examples of the use of RT-PCR to confirm the presence of viruses detected by Illumina sequencing in sampled bean tissues. RT-PCR using primers specific for BCMNV, CMV and PvEV1 was performed on samples collected from two sites in Kenya: Kabete (Nairobi) and Kirinyaga. M, 1kb Plus DNA ladder; NTC, non-template control.
