# Supplementary material for: Metagenomic Analysis of Plant Virus Occurrence in Common Bean (Phaseolus vulgaris) in Central Kenya
Source: Front Microbiol. 2018 Dec 7;9:2939. doi: 10.3389/fmicb.2018.02939 (PMC6293961; doi:10.3389/fmicb.2018.02939)
Supplement: Supplementary file 4 [file Data_Sheet_4.PDF]

Supplemental Table S1. Index identity (ID) and index primers used in this study.

Sample ID	Index ID	Index
S1	A002	CGATGT
S2	A004	TGACCA
S3	A005	ACAGTG
S4	A006	GCCAAT
S5	A007	CAGATC
S6	A012	CTTGTA
S7	A014	AGTTCC
S8	A015	ATGTCA
S9	A013	AGTCAA
S10	A016	CCGTCC
S11	A018	GTCCGC
S12	A019	GTGAAA
