# Supplementary material for: Metagenomic Analysis of Plant Virus Occurrence in Common Bean (Phaseolus vulgaris) in Central Kenya
Source: Front Microbiol. 2018 Dec 7;9:2939. doi: 10.3389/fmicb.2018.02939 (PMC6293961; doi:10.3389/fmicb.2018.02939)
Supplement: Supplementary file 5 [file Data_Sheet_5.PDF]

Supplemental Table S2. Sample identity (ID) and virus sequences identified in each sample and their GenBank accession numbers.

Sample ID	Virus	GenBank Accession number
S1	PvEV 1	MH567335
	PvEV 2	MH567336
S2	BCMNV	MH567337
S3	BCMNV	MH169563
	PvEV 1	MH567338
	PvEV 2	MH567339
S4	BCMNV	MH169564
S5	BCMNV	MH169565
S6	BCMNV	MH169566
S7	BCMNV	MH169567
S8	BCMNV	MH169568
S9	BCMNV	MH169569
S10	BCMNV	MH567340
	PvEV1	MH567341
	CMV_RNA1	MH567342
	CMV_RNA2	MH567343
	CMV_RNA3	MH567344
S11	BCMNV	MH567345
	PvEV1	MH567346
	CMV_RNA1	MH567347
	CMV_RNA2	MH567348
	CMV_RNA3	MH567349
S12	BCMNV	MH567350
	PvEV1	MH567351
	CMV_RNA1	MH567352
	CMV_RNA2	MH567353
	CMV_RNA3	MH567354

BCMNV: Bean common mosaic necrosis virus
PvEV: *Phaseolus vulgaris* Endornavirus
CMV: Cucumber mosaic virus RNAs 1, 2, and 3
