# Supplementary figures and images for: Metagenomic Analysis of Plant Virus Occurrence in Common Bean (Phaseolus vulgaris) in Central Kenya
Source: Front Microbiol. 2018 Dec 7;9:2939. doi: 10.3389/fmicb.2018.02939 (PMC6293961; doi:10.3389/fmicb.2018.02939)

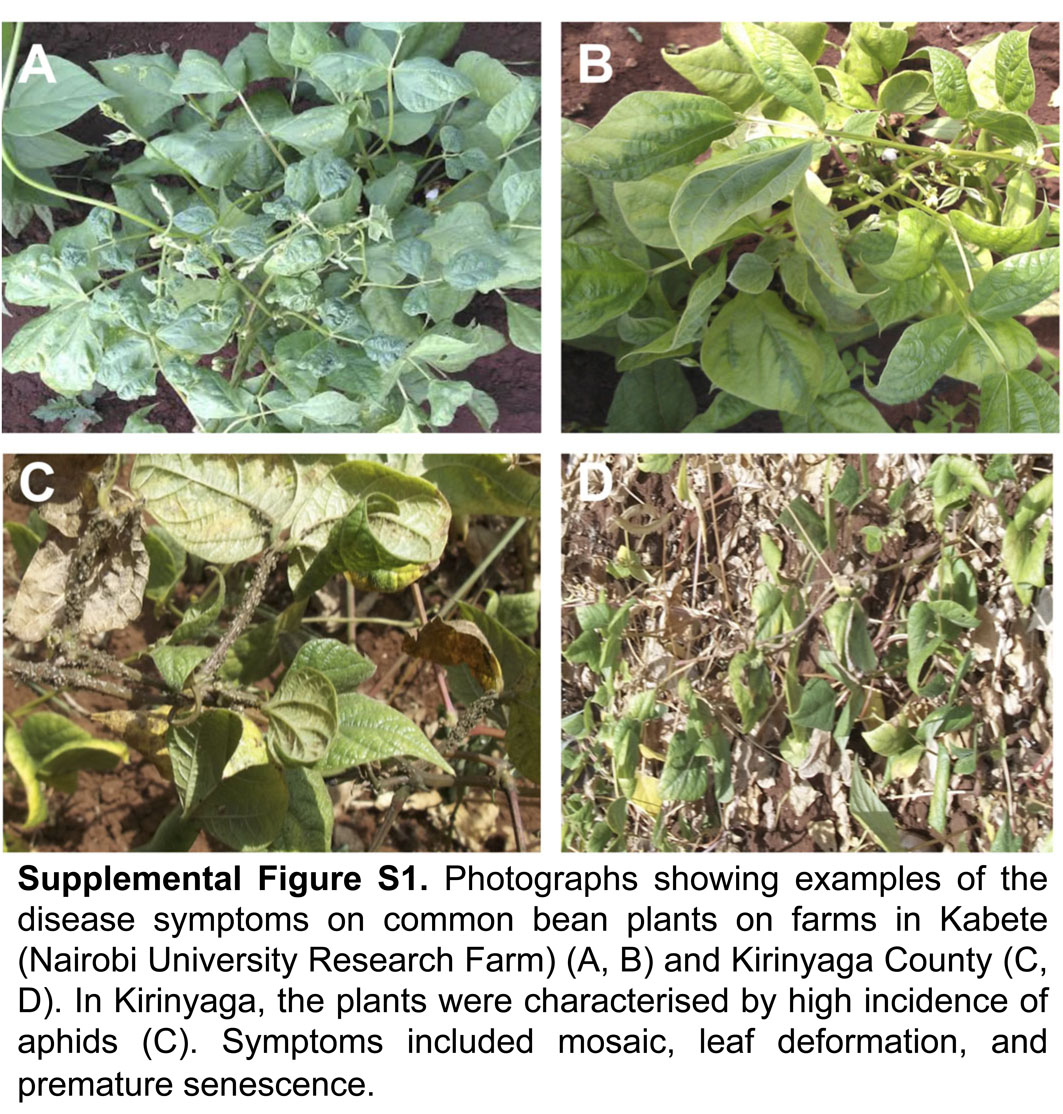

Supplement: Supplementary file 6 [file Image_1.JPEG]
